# Wearable Sensor Technologies to Assess Motor Functions in People With Multiple Sclerosis: Systematic Scoping Review and Perspective

**DOI:** 10.2196/44428

**Published:** 2023-07-27

**Authors:** Tim Woelfle, Lucie Bourguignon, Johannes Lorscheider, Ludwig Kappos, Yvonne Naegelin, Catherine Ruth Jutzeler

**Affiliations:** 1 Research Center for Clinical Neuroimmunology and Neuroscience Basel University Hospital and University of Basel Basel Switzerland; 2 Department of Neurology University Hospital Basel Basel Switzerland; 3 Department of Health Sciences and Technology ETH Zurich Zürich Switzerland

**Keywords:** multiple sclerosis, digital biomarkers, digital health technologies, digital mobility outcomes, wearables, sensors, inertial motion unit, accelerometry, actigraphy, review

## Abstract

**Background:**

Wearable sensor technologies have the potential to improve monitoring in people with multiple sclerosis (MS) and inform timely disease management decisions. Evidence of the utility of wearable sensor technologies in people with MS is accumulating but is generally limited to specific subgroups of patients, clinical or laboratory settings, and functional domains.

**Objective:**

This review aims to provide a comprehensive overview of all studies that have used wearable sensors to assess, monitor, and quantify motor function in people with MS during daily activities or in a controlled laboratory setting and to shed light on the technological advances over the past decades.

**Methods:**

We systematically reviewed studies on wearable sensors to assess the motor performance of people with MS. We scanned PubMed, Scopus, Embase, and Web of Science databases until December 31, 2022, considering search terms “multiple sclerosis” and those associated with wearable technologies and included all studies assessing motor functions. The types of results from relevant studies were systematically mapped into 9 predefined categories (association with clinical scores or other measures; test-retest reliability; group differences, 3 types; responsiveness to change or intervention; and acceptability to study participants), and the reporting quality was determined through 9 questions. We followed the PRISMA (Preferred Reporting Items for Systematic Reviews and Meta-Analyses) reporting guidelines.

**Results:**

Of the 1251 identified publications, 308 were included: 176 (57.1%) in a real-world context, 107 (34.7%) in a laboratory context, and 25 (8.1%) in a mixed context. Most publications studied physical activity (196/308, 63.6%), followed by gait (81/308, 26.3%), dexterity or tremor (38/308, 12.3%), and balance (34/308, 11%). In the laboratory setting, outcome measures included (in addition to clinical severity scores) 2- and 6-minute walking tests, timed 25-foot walking test, timed up and go, stair climbing, balance tests, and finger-to-nose test, among others. The most popular anatomical landmarks for wearable placement were the waist, wrist, and lower back. Triaxial accelerometers were most commonly used (229/308, 74.4%). A surge in the number of sensors embedded in smartphones and smartwatches has been observed. Overall, the reporting quality was good.

**Conclusions:**

Continuous monitoring with wearable sensors could optimize the management of people with MS, but some hurdles still exist to full clinical adoption of digital monitoring. Despite a possible publication bias and vast heterogeneity in the outcomes reported, our review provides an overview of the current literature on wearable sensor technologies used for people with MS and highlights shortcomings, such as the lack of harmonization, transparency in reporting methods and results, and limited data availability for the research community. These limitations need to be addressed for the growing implementation of wearable sensor technologies in clinical routine and clinical trials, which is of utmost importance for further progress in clinical research and daily management of people with MS.

**Trial Registration:**

PROSPERO CRD42021243249; https://www.crd.york.ac.uk/prospero/display_record.php?RecordID=243249

## Introduction

### Background

Multiple sclerosis (MS) is a chronic inflammatory and degenerative demyelinating disease of the central nervous system that can lead to a wide range of sensorimotor, cognitive, visual, and autonomic function symptoms [1]. As the disease progresses, these symptoms become more prominent, impede the performance of activities of daily living, and reduce the quality of life considerably [2]. Approved disease-modifying therapies reduce the risk of relapse and may slow down progression but do not cure MS [3]. The course of MS on an individual basis remains largely unpredictable with substantial clinical heterogeneity. Given the increasing number of therapeutic choices, close monitoring of the disease course and development of reliable and sensitive tools to continuously monitor MS symptoms and to detect both progression independent of relapse activity and relapse-associated worsening are of paramount importance for disease management [4]. Conventional MS outcome measures are usually assessed episodically and only provide snapshots of the disease course. Intermittent in-clinic monitoring is limited in detecting fluctuations of symptoms, particularly comorbidities and fatigue [5,6].

Recent advances in wearable sensors and digital health technologies [7] may enable individualized and quasicontinuous remote monitoring of motor functions in both real-world and controlled laboratory settings, potentially benefiting both clinical routine and clinical trials [8]. Different types of sensors (eg, accelerometers, gyroscopes, and magnetometers) are embedded into different types of wearables (eg, motion trackers, inertial measurement units, smartwatches, or smartphones). These sensors have been used extensively to assess physical activity and ambulation in people with MS [9-12]. Other promising applications of wearable sensor technologies in the area of MS include the assessment of gait [13-15], balance and postural control [16,17], and dexterity and tremor [18].

Sensor data are collected either through passive monitoring (wearing the device in normal everyday life) or through active tests (performed at defined time points) and are transformed into metrics (so-called features) by designated algorithms. For example, physical activity is often measured passively using features such as “step count per day” or “distance walked per day.” Such features are classified as digital measures, defined as objective quantifiable measures of physiology measured using digital tools [19]. Sensor technologies and their digital measures must undergo a thorough process of verification and analytical and clinical validation before they can be safely deployed as digital biomarkers, especially in the context of clinical trials [7,19-22]. Commercial activity trackers or “general purpose computing platforms” (eg, smartwatches or smartphones) can be used as long as they are carefully shown to be fit for purpose—they need not necessarily be approved medical devices [7]. Consequently, commercial wearable sensor technologies are already being used as secondary and primary outcomes in industry-sponsored clinical trials for many indications, including MS [23].

### Objectives

Considering the rapid progress of and innovations in wearable sensor technologies in the field of MS, it is crucial to collate and critically evaluate state-of-the-art assessments. Thus, the main objective of our review was to provide a comprehensive overview of the different wearable sensor technologies that have been implemented to assess motor function (ie, physical activity, gait, balance, and dexterity or tremor) in people with MS. To this end, we systematically reviewed the literature, mapped different functional domains examined and types of results reported, and performed a reporting quality assessment of the included studies. On the basis of our findings, we provide recommendations for future investigations of wearable sensor technologies as monitoring tools for motor functions in people with MS. Finally, we created an open-access web platform that allows to interactively explore the results of this review in more detail.

## Methods

The study protocol was registered with and approved by PROSPERO before the start of the study (CRD42021243249) [24]. We followed the PRISMA (Preferred Reporting Items for Systematic Reviews and Meta-Analyses) statement [25] (Multimedia Appendix 1).

### Search Strategy and Selection of Studies

We searched the PubMed, Scopus, Embase, and Web of Science databases for the first time on March 22, 2021. We performed a second search on February 19, 2023, limiting publication time from March 22, 2021, to December 31, 2022. Both searches were performed without any language restrictions. The prespecified search terms and inclusion and exclusion criteria for the selection of the studies are shown in Textbox 1. Abstract review was performed independently by 2 out of 5 investigators (TW, LB, JL, YN, and CRJ). All disagreements were discussed and resolved at a consensus meeting.

**Prespecified search terms, included article types, and study selection criteria (inclusion and exclusion criteria).**

**Databases searched up to December 31, 2022, without language restrictions**

- PubMed
- Scopus
- Embase
- Web of Science

**Search terms**

- "multiple sclerosis" AND ("smartphone" OR "smartphones" OR "smartwatch" OR "smartwatches" OR "wearable" OR "wearables" OR "biosensor" OR "biosensors" OR "digital biomarker" OR "digital biomarkers" OR "accelerometer" OR "accelerometers" OR "accelerometry" OR "gyroscope" OR "gyroscopes" OR "inertial motion unit" OR "IMU")

**Article types**

- Included articles
  - Peer-reviewed conference papers
  - Peer-reviewed journal articles
- Excluded articles
  - Case reports
  - Commentaries, perspectives, and opinion papers
  - Conference abstracts
  - Patent applications
  - Preprints
  - Reviews and meta-analyses

**Selection criteria**

- Inclusion criteria
  - Studies using mobile or wearable sensor technologies (including smartphone apps)
  - Studies assessing motor function (including physical activity, gait, balance, and dexterity or tremor)
  - Adult and pediatric multiple sclerosis populations
  - Cross-sectional, longitudinal, retrospective, prospective, and controlled studies
- Exclusion criteria
  - Studies purely using nonwearable technologies (eg, static camera based or instrumented walkways)
  - Studies not assessing motor function (eg, purely neurocognitive or ophthalmologic)
  - Studies without people with multiple sclerosis (eg, animal studies)

### Data Extraction and Synthesis

Data extraction was distributed among 4 investigators (TW, LB, YN, and CRJ) and performed independently. For each study, we extracted the characteristics of the study population (sample size, sex, age, type of MS, severity, duration, treatment of MS, comorbidities, and type of controls, if applicable), information on sensor technology (name, brand, type of sensors, number, anatomical position, and context [ie, real-world and controlled laboratory setting]), types of results, and assessment of quality. Bibliographic data were retrieved from OpenAlex (OurResearch) to construct the local citation network and coauthorship network of all selected studies [26]. The URLs for the GitHub repository containing all code and data [27], for the interactive Shiny app supporting this study [28], and for the interactive local citation network and coauthorship network of the included studies [29] have been included.

### Systematic Mapping of Types of Results

We systematically mapped and categorized the results of the studies included. The same investigators who performed the data extraction classified the studies according to the functional domains covered (Textbox 2) and the types of results reported (Table 1). In addition, the significance of statistical tests performed for each applicable result was categorized as “nonsignificant,” “some significant” (mixed results), “significant,” and “significance not tested.” Note that studies can report multiple functional domains and types of results at once and that the 9 types of results are not necessarily exhaustive, meaning that some studies potentially addressed questions not related to any of these types of results.

**Functional domains covered.**

**Physical activity**

- Usually determined by actigraphy or accelerometry (eg, minutes of moderate or vigorous physical activity per day, activity counts per day, number of steps per day, or estimated distance per day)

**Gait**

- Qualitative gait features, such as walking speed, cadence, swing or stance time, symmetry, joint angles, often determined through (multiple) inertial motion units

**Balance**

- Postural stability (eg, determined through sway in static balance tests or determined through fall detection)

**Dexterity or tremor**

- Ability of upper or lower extremities to perform coordinated movements without tremor

**Table 1:** Types of results reported.

Category	Description
Correlation or association with clinical MS^a^ severity scores	Are digital outcomes (eg, step count and speed) correlated or associated with the disease severity measured by clinical scores (eg, EDSS^b^ or PDDS^c^)?
Correlation or association with other measures	Are digital outcomes (eg, step count and speed) correlated or associated with other disease-specific outcomes (eg, 2MWT^d^, imaging outcomes, retinal nerve fiber layer thickness)?
Test-retest reliability	Do digital outcomes reliably report consistent results when tested repeatedly in stable conditions?
Group difference (MS vs healthy controls)	Can digital outcomes distinguish people with multiple sclerosis and healthy controls?
Group difference (MS vs MS)	Can digital outcomes distinguish subgroups of people with multiple sclerosis (eg, fallers vs nonfallers)?
Group difference (MS vs other diseases)	Can digital outcomes distinguish people with multiple sclerosis from participants with other pathologies (eg, Parkinson disease)?
Responsiveness to change (longitudinal)	Are digital outcomes responsive to change (eg, natural disease progression)?
Responsiveness to intervention	Are digital outcomes responsive to interventions (eg, pharmacological intervention or rehabilitation)?
Subjective participant acceptability	Are the digital outcomes subjectively meaningful and acceptable to participants?

^a^MS: multiple sclerosis.

^b^EDSS: Expanded Disability Status Scale.

^c^PDDS: Patient Determined Disease Steps.

^d^2MWT: 2-minute walking test.

### Reporting Quality Assessment

We assigned scores according to the reporting quality of the included studies based on the presence of the following: (1) a clear research objective (including an outcome); (2) inclusion and exclusion criteria; (3) population demographics, including at least age and sex; (4) assessment of MS in terms of type and severity (Expanded Disability Status Scale [EDSS] [30] or Patient Determined Disease Steps [PDDS]) [31]; (5) description of sensor technology used in terms of type, positioning, and context in which it was worn (real-world setting and laboratory setting) and recording frequency; (6) reporting of appropriate statistical analysis (eg, corrections for multiple comparisons); (7) a description of the robustness of the results (eg, ways of calculating effect sizes, reporting CIs, and SDs); (8) the availability of data and code used for analyses; and (9) discussion on the limitations of the study. These criteria were readapted from the systematic review by Qiao [32] to match the objectives of this review.

Each criterion was formulated as a question and scored as “yes,” “partially,” or “no.” For each study, the reporting quality assessment was performed by the author who extracted the data from the previous step. Difficult or ambiguous ratings were discussed within the group, and the final scores for each publication were determined. Publications satisfying all the 9 criteria with “yes” were considered “very good,” publications satisfying 6 to 8 criteria with at least “partially” were considered “good,” and all other publications were considered “substandard.”

## Results

### Characteristics of Included Studies

**Figure 1:**
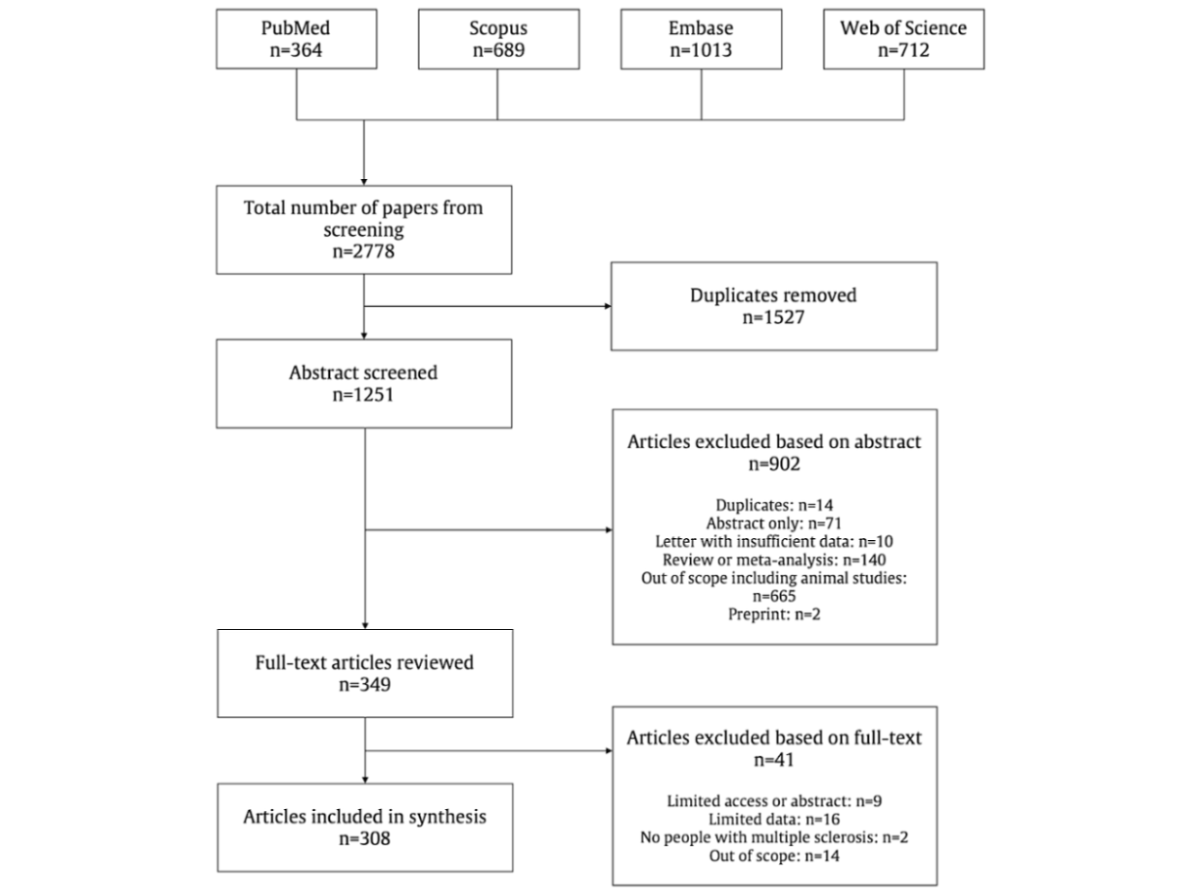
PRISMA (Preferred Reporting Items for Systematic Reviews and Meta-Analyses) flowchart.

The search of electronic databases yielded 2778 records of 1251 publications after the removal of duplicates. Abstract screening by 2 investigators identified 349 publications for full-text reviews. The interrater agreement for this selection was substantial (Cohen κ=0.65, 95% CI 0.61-0.70; 1078/1251, 86.17% agreement, 173/1251, 13.82% studies with initial disagreement). Of those, the inclusion criteria for this systematic review were met by 88.3% (308/349) of studies (Figure 1) [17,18,31,33-337]. Apart from 1 German study [33], all studies were published in English. The local citation network of all included studies can be found in Multimedia Appendix 2 and the coauthorship network can be found in Multimedia Appendix 3. The URLs for the complementary interactive web apps are included [28,29].

On average, real-world studies included 63 (IQR 30-143) people with MS, laboratory studies 29 (IQR 17-50), and mixed studies 30 (IQR 25-44). The overall range was from 3 to 1355 people with MS (Table 2). There were 8 studies with ≥500 people with MS, all performed after 2012 (Multimedia Appendix 4). The majority of the studies enrolled ≥50% female individuals with MS (median 76% for real-world studies, IQR 68%-82%, range: 0%-100%), reflecting the higher prevalence of MS among women. In terms of study population, 303 (98.4%) out of 308 studies enrolled adults with MS only, whereas 5 (1.6%) out of 308 focused on pediatric populations [34-38].

Multimedia Appendices 5-7 ([17,18,31,33-337]) refer to all publications included in our review. The primary context of sensor technology ranged from real-world settings (176/308, 57.1% of studies; Multimedia Appendix 5) to controlled laboratory settings (107/308, 34.7% of studies; Multimedia Appendix 6), or a mixture of both (25/308, 8.1% of studies; Multimedia Appendix 7). While early studies were almost exclusively performed in the real-world setting, the proportion of studies performed in a controlled laboratory or clinical setting has risen over time (Multimedia Appendix 8). In the real-world setting, mostly actigraphy and accelerometry (eg, activity counts, steps, estimated distance per day) were used as a surrogate marker for physical activity, along with self-reported measures or clinical assessments. In the laboratory setting, outcome measures included the 2-minute walking test (2MWT) [39], 6-minute walking test [338], timed 25-foot walking test (T25FW) [339], timed up and go (TUG) [340], stair climbing, balance test (Berg Scale), spasticity test [341], tremor test (Fahn tremor rating scale) [342], and finger-to-nose test [40]. The aim of the mixed setting studies was to characterize motor functions in both real-world and laboratory conditions for a group of participants.

**Table 2:** Detailed information on all included studies.

				Real-world setting (n=176)		Laboratory setting (n=107)		Mixed setting (n=25)
**Population**								
	Number of people with multiple sclerosis enrolled, median (IQR; range)		63 (30-143; 9-1355)		29 (17-50; 3-562)		30 (25-44; 10-212)	
	Proportion female (%), median (IQR; range)		76 (68-82; 27-93)^a^		69 (61-75; 0-100)^b^		66 (54-80; 25-92)^c^	
	Average age (years), median (IQR; range)		48 (45-51; 16-66)^d^		48 (43-52; 35-63)^a^		49 (47-51; 40-58)	
**Type of MS**								
	Proportion relapsing-remitting (%), median (IQR; range)		83 (68-90; 0-100)^e^		77 (50-99; 0-100)^f^		70 (55-95; 0-100)^g^	
	Proportion progressive (%), median (IQR; range)		11 (0-27; 0-100)^e^		20 (0-44; 0-100)^f^		30 (0-43; 0-100)^g^	
**Study characteristics, n (%)**								
	Total number of studies		176 (100)		107 (100)		25 (100)	
	Studies with comparator groups		70 (39.8)		78 (72.9)		12 (48)	
	Healthy controls		50 (28.4)		66 (61.7)		9 (36)	
	Multiple sclerosis controls		18 (10.2)		9 (8.4)		3 (12)	
	Other diseases^h^		2 (1.1)		3 (2.8)		0 (0)	
**Number of publications per position, n (%)**								
	Sternum		2 (1.1)		22 (20.6)		4 (16)	
	Hand		1 (0.6)		2 (1.9)		0 (0)	
	Upper back		6 (3.4)		43 (40.2)		6 (24)	
	Lower back		96 (54.5)		17 (15.9)		11 (44)	
	Waist		2 (1.1)		5 (4.7)		0 (0)	
	Upper arm		0 (0)		6 (5.6)		0 (0)	
	Lower arm		27 (15.3)		19 (17.8)		5 (20)	
	Wrist		17 (9.7)		8 (7.5)		3 (12)	
	Upper leg		17 (9.7)		13 (12.1)		3 (12)	
	Lower leg		1 (0.6)		22 (20.6)		4 (16)	
	Ankle		10 (5.7)		18 (16.8)		4 (16)	
	Foot		4 (2.3)		21 (19.6)		4 (16)	
	Other positions^i^		6 (3.4)		10 (9.3)		1 (4)	
	Not reported		31 (17.6)		4 (3.7)		2 (8)	
**Number of publications per sensor type (one publication can use multiple types of sensors** **), n (%)**								
	**Accelerometer**		165 (93.8)		101 (94.4)		23 (92)	
		Uniaxial	48 (27.3)		8 (7.5)		1 (4)	
		Biaxial	5 (2.8)		2 (1.9)		1 (4)	
		Triaxial	114 (64.8)		92 (86)		23 (92)	
	Gyroscope		18 (10.2)		65 (60.7)		8 (32)	
	Magnetometer		6 (3.4)		48 (44.9)		5 (20)	
	Touchscreen		16 (9.1)		3 (2.8)		2 (8)	
	Mechanical pedometer		10 (5.7)		1 (0.9)		0 (0)	
	Other sensor types^j^		10 (5.7)		13 (12.1)		3 (12)	

^a^Data not available in 11 studies.

^b^Data not available in 13 studies.

^c^Data not available in 4 studies.

^d^Data not available in 7 studies.

^e^Data not available in 34 studies.

^f^Data not available in 44 studies.

^g^Data not available in 6 studies.

^h^Other diseases include stroke, Parkinson disease, and rheumatoid arthritis.

^i^Other positions include bag or pocket, tip of the cane or crutches, and head.

^j^Other sensor types include electrocardiogram, GPS, surface electromyography, portable metabolic system (VO_2_), skin impedance, force sensor, barometer, and thermometer.

### Information on Sensor Technologies Used

**Figure 2:**
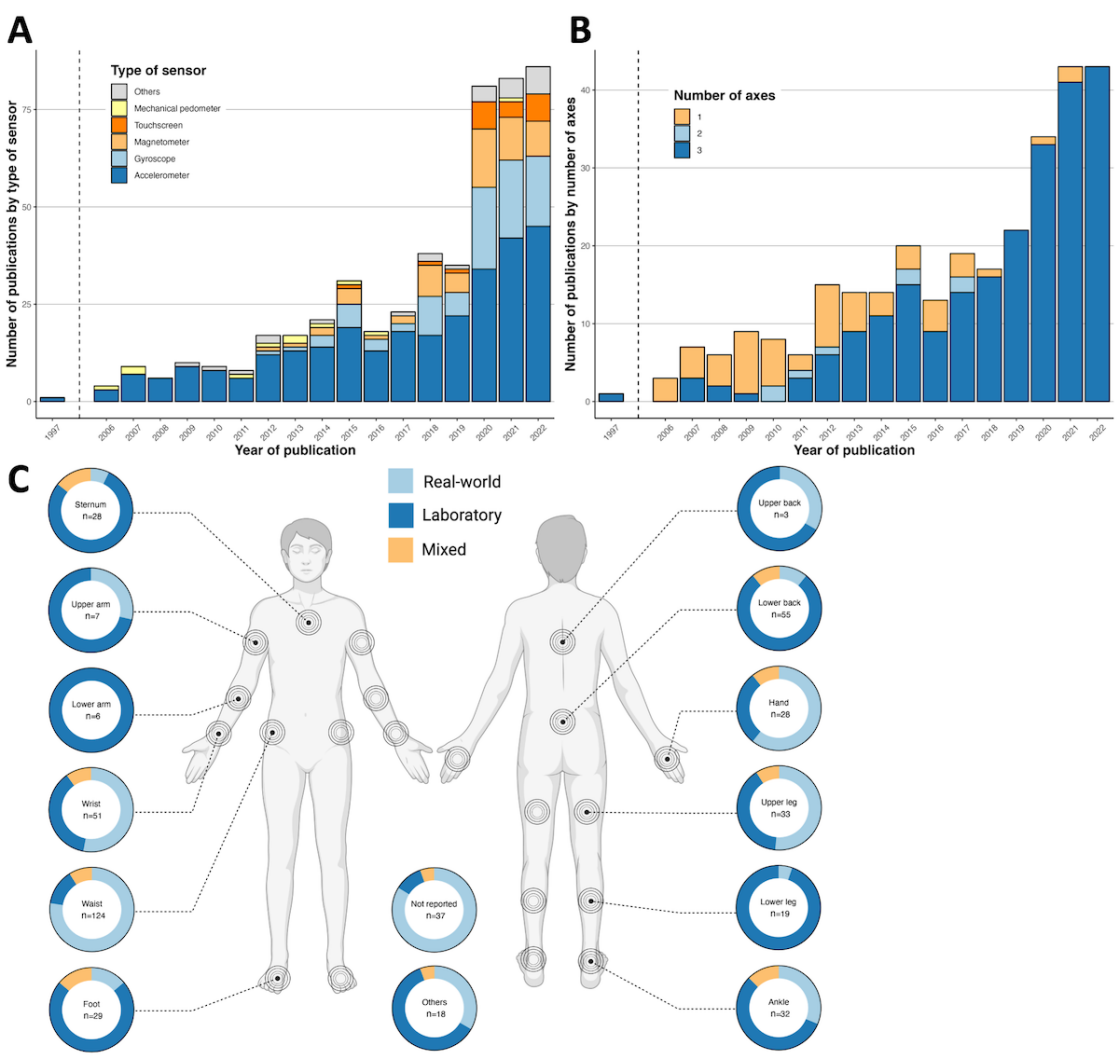
Information on wearables and sensors. (A) Number of publications published per year and per type of sensor used. Publications using multiple types of sensors count multiple times, once for each type of sensor. Others: electrocardiogram, GPS, surface electromyography, portable metabolic system (VO2), skin impedance, force sensor, barometer, and thermometer. (B) Number of publications published per year and per type of accelerometer used. Publications using multiple types of accelerometers count multiple times, once for each type of accelerometer. (C) Distribution of the sensors per physical position and context in which it was worn (real-world setting, controlled laboratory setting, and mixed setting); others: head, pocket, bag, chest, and crutches.

As shown in Figure 2A, the most commonly used sensor type is the accelerometer (alone or in combination with a gyroscope or magnetometer). Smartphone touchscreens were used in 21 publications, mostly from 2020. With technological advances over the last few decades, the number of axes in accelerometers has gradually increased. Although in the early 2000s, most accelerometers relied on one axis, currently triaxial sensors are state-of-the-art (Figure 2B).

The most popular anatomical landmarks for wearable placement in the real-world setting were the waist (96/176, 54.5% publications), wrist (27/176, 15.3%), and hand and upper leg (17/176 each, 9.7%). Multiple mounting positions were typically chosen in the laboratory and mixed settings (Figure 2C). The majority of the real-world studies equipped their participants with only 1 wearable (146/176, 83%), while 59.8% (64/107) of laboratory setting studies mounted multiple wearables (Multimedia Appendix 9). The number of wearables mounted on the upper and lower extremities has vastly increased over time (Multimedia Appendix 10). Multiple wearables are advantageous for assessing the role and interplay of different body parts during physical activity. A controlled laboratory setting simplifies the use of multiple wearables. However, in a real-world setting, multiple wearables are less practical or less feasible and potentially reduce the compliance of the participants. Refer to the Shiny app [28] for more detailed information on the wearables and their sensors.

### Systematic Mapping of Types of Results

#### Overview

The functional domain physical activity was most commonly examined (196/308, 63.6%), followed by gait (81/308, 26.3%), dexterity or tremor (38/308, 12.3%), and balance (34/308, 11%). Most publications only covered 1 functional domain at a time, but 22 publications covered 2 domains, 6 publications covered 3 domains [17,40-44], and 2 publications covered all 4 domains [45,46] simultaneously. Starting in 2015, the proportion of publications examining functional domains other than physical activity has increased markedly (Multimedia Appendix 11).

#### Association With Clinical MS Severity Scores

**Figure 3:**
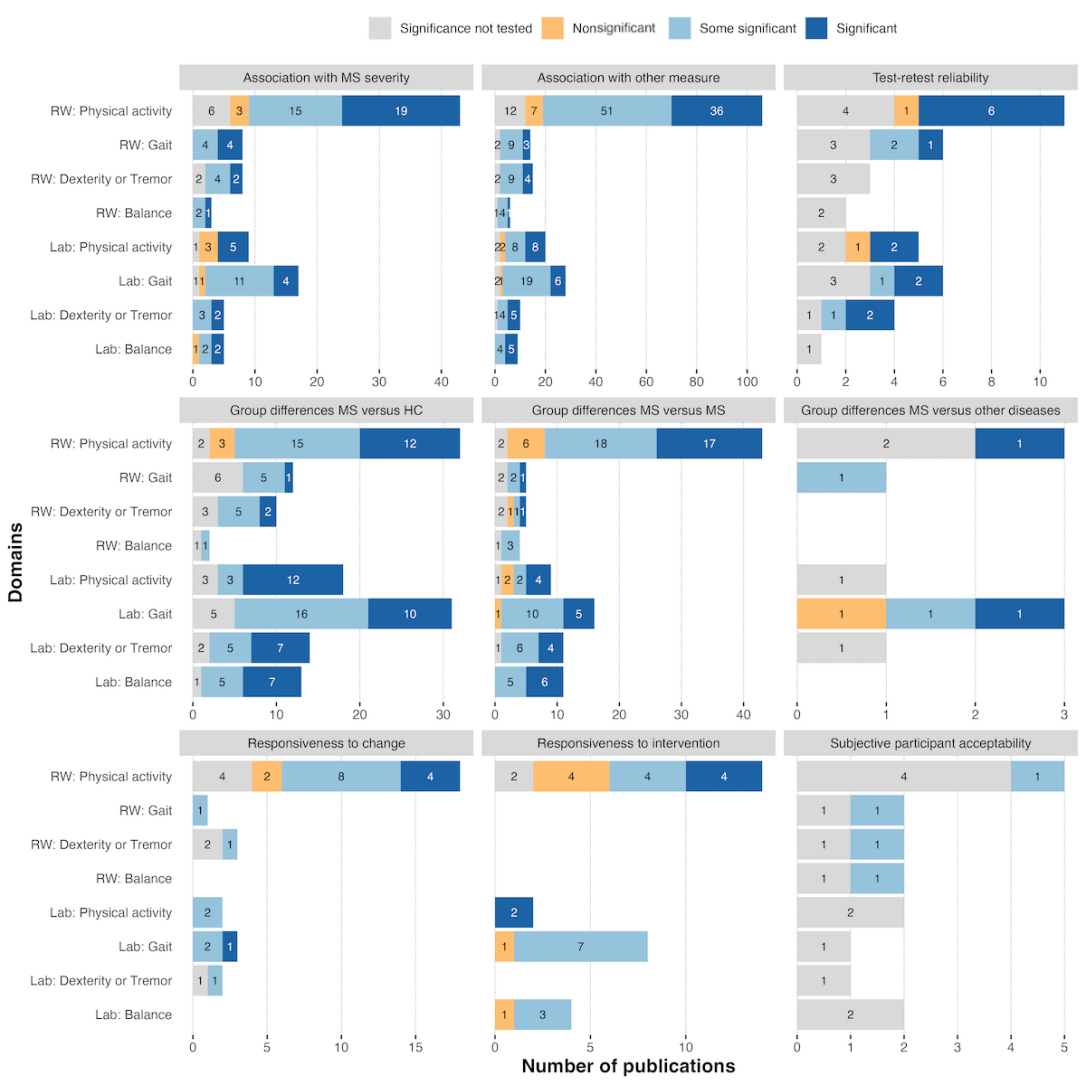
Results reported by domain and context studied. (A) Association with clinical severity score (Expanded Disability Status Scale or Patient Determined Disease Steps); (B) association with other measure; (C) test-retest reliability; (D) group differences (multiple sclerosis [MS] vs healthy controls [HC]); (E) group differences (MS vs MS); (F) group differences (MS vs other diseases), including Parkinson disease, stroke, and rheumatoid arthritis; (G) responsiveness to change; (H) responsiveness to intervention (controlled study); and (I) subjective participant acceptability. RW: real-world setting.

A total of 81 studies described the relationship between standard clinical tests of disease severity and wearable-derived features of motor function in people with MS (Figure 3A). Commonly used MS impairment and disability metrics include EDSS and PDDS. For instance, Fjeldstad et al [47] demonstrated that accelerometers can objectively quantify physical activity levels in people with MS across varying levels of disability as measured by the EDSS [47]. The amount of physical activity captured by accelerometry is inversely proportional to the degree of physical disability. Similar correlations were found between the PDDS score and different accelerometric features (eg, mean and maximum step counts per day) [48]. A majority of the studies found a significant association between clinical MS severity scores and features derived from wearable devices.

#### Association With Other Measures

Wearable-derived features are frequently correlated or associated with the outcomes of specific clinical measures, such as the 2MWT, 6-minute walking test, T25FW, and TUG for mobility or the 9-Hole Peg Test for dexterity [49-54]. Other measures can also include clinical questionnaires or, for technical validation, nonwearable gold standard measurements of motor function, such as instrumented walkways or optical motion tracking. A total of 180 studies reported this type of correlation or association between wearable-derived features and other outcome measures (Figure 3B). Many of these correlations or associations were found to be significant.

#### Test-Retest Reliability

We identified 30 studies that assessed the test-retest reliability of sensor technologies (Figure 3C). The intraclass correlation coefficient is a common measure of test-retest reliability. Most studies have found sensor technologies to produce reliable measures at different time points. Typically, the assessments were 1 day [55], 1 week [56], or even 6 months apart [57].

#### Group Differences

Wearable sensor technologies were compared between people with MS and (matched) healthy controls in 110 studies (Figure 3D) [58,59], between subpopulations of people with MS based on symptoms or disease progression in 90 studies (eg, tremor vs nontremor participants and different levels of activity; Figure 3E) [47,60-62], or distinguish MS from other neurological diseases in 9 studies (eg, Parkinson disease; Figure 3F). The suitability of wearable sensor technologies for detecting group differences was demonstrated in a majority of the studies. Identifying subgroups or different disease phenotypes is of great clinical relevance, as it may allow the tailoring of interventional strategies to the specific characteristics of different patient groups, as opposed to the one-size-fits-all strategy.

#### Responsiveness to Change

A total of 26 studies investigated the usefulness of sensor technologies in detecting and tracking changes over time (Figure 3G). Responsiveness of the sensors was defined as the ability to detect a significant change between pairs of assessments separated in time (eg, days, weeks, or months between assessments). A total of 17 of these 26 (68%) publications reported at least some significant results [54,63-78].

#### Responsiveness to Intervention

A total of 22 studies used sensor technologies within the framework of clinical trials to provide an objective measure of the impact of an intervention on physical activity (Figure 3H) [79,80]. For example, the FAMPKIN study showed that prolonged-release fampridine significantly improved walking speed, endurance, and everyday physical activity in a subset of people with MS (ie, responders) [80]. The latter was measured using an accelerometer attached to the ankle for 14 consecutive days during each of the 2 double-blind treatment phases. Another double-blind placebo-controlled crossover study investigated the effect of extended-release dalfampridine on physical activity [79]. Accelerometer outcomes (number of steps per day and total ambulatory time) were comparable between the dalfampridine and placebo groups. In contrast, the results derived from the self-reported Physical Activity and Disability Survey–Revised [343] indicated a therapeutic effect of dalfampridine on physical activity [79].

#### Subjective Participant Acceptability

We found only 9 studies investigating subjective meaningfulness and acceptability of wearables among people with MS through questionnaires (Figure 3I) [45,46,81-87]. They generally reported favorable opinions, and the majority of people with MS would have liked to continue using the wearables.

### Reporting Quality Assessment

The results of the reporting quality assessment are presented in Multimedia Appendix 12 [17,18,31,33-337]. Reporting quality was classified as very good in 3 of 308 (1%) publications [88-90], as good for the vast majority of publications (298/308, 96.8%), and as substandard for 7 of 308 (2.3%) publications. Most of the publications provided sufficient details on the study’s objective, demographics, disease characteristics, and the strength of the results (eg, ways of calculating effect sizes or reporting CIs or SD). In contrast, only a few studies provided the data or the code used for data preparation and analysis (16/308, 5.2%) [40-42,52,59,88-98]. A remarkable number of studies did not report any limitations (24/308, 7.8%). The most frequently mentioned limitations were a small sample size and narrow inclusion criteria, which did not allow the generalization of the findings across all people with MS.

## Discussion

### Principal Findings

Effective management of people with MS requires frequent and objective measurement of the patient’s condition during normal daily activity. However, conventional MS outcome measures provide only a momentary snapshot of the disease course and have several limitations, including high interrater variability as well as recall and desirability bias [344-346]. A promising option to shift from traditional clinical assessments to objective and continuous disease monitoring in people with MS resides is the use of wearable sensor technologies. These technologies have the potential to transform health care for people with MS and are thus increasingly used in clinical studies to assess and track physical activity and other MS-related motor outcomes with time-series analyses of unprecedented temporal resolution. The aim of this review is to provide a comprehensive overview of the currently used wearable sensor technologies and shed light on technological advances over the last few decades. Moreover, we assembled the existing evidence on the suitability and reliability of sensor technologies in the context of clinical routine (associations with clinical measures and differentiation of patient subgroups) and clinical trials (responsiveness to treatments).

Similar to other medical fields [347], wearable sensor technologies used to monitor disease progression and assess the propensity of people with MS to engage in physical activity have undergone a rapid transformation in the past few decades. Early applications of sensor technologies involved mechanical pedometers and monoaxial accelerometers, transitioned to multiaxial accelerometers or gyroscopes, and finally to phone-based monitoring. Although this technology has also progressed from real-world to laboratory and mixed settings over the last 2 decades, the majority of studies were still conducted in real-world and mixed settings.

Comparing sensor technologies with clinical scales (eg, EDSS and PDDS) and performance measures (eg, 2MWT, TUG, and T25FW) in a controlled laboratory and clinical setting is an important strategy for gathering information about the underlying biomechanics and neurophysiological aspects of the specific tasks. Often, these laboratory studies make use of multiple wearables in multiple positions to assess the role and interplay between different body parts and laterality during gait tasks [53,99-101], postural sway [102-106], tremor [102-107], and gait-related fatigue [108-110]. This is in contrast to real-world studies, which usually use 1 or 2 wearables only, presumably because of usability and adherence. It remains to be seen whether findings from multiwearable laboratory studies can be replicated in single wearable real-world studies in the future.

Similar to traditional clinical assessments, studies in a laboratory setting are somewhat limited in terms of their observation window (minutes to hours) and the environment (generally safe, no obstacles). Continuous monitoring of physical activity under real-world conditions, on the other hand, allows taking into consideration environmentally induced tradeoffs, which inadvertently result in behavior modifications (eg, prioritizing safety over efficiency in terms of walking pattern) [348,349]. Analysis of continuous monitoring must consider the possibility of long-term practice effects, which have been described for dexterity tests but not mobility tests [41]. To leverage the benefits of both real-world and laboratory settings, a growing number of studies have assessed MS-related motor outcomes in the clinic combined with continuous physical activity monitoring in free-living conditions.

This review identified 3 major applications of sensor technology in people with MS: identifying subgroups of people with MS, monitoring disease progression, and tracking responses to interventions. Since the early 2000s, several studies have investigated whether digital outcomes are associated with clinical scores (eg, disease severity) or other disease-specific outcomes (eg, imaging outcomes). Findings from these studies indicate that digital outcomes are correlated with clinical and disease-specific outcomes, suggesting that they could serve as potential proxies for certain aspects of the disease (eg, severity). Validation and reliability studies aim at implementing sensor technologies in the clinical routine for diagnosis and prognosis. Digital monitoring of people with MS was also found to aid in the identification of patient subgroups based on differences in physical activity. Further studies are needed to demonstrate whether digital monitoring can detect subtle changes that might be indicative of disease progression in people with MS in general or in subgroups (eg, fallers vs nonfallers). Since 2015, there has been an increasing trend in studies focusing on using wearable and mobile devices for predicting or quantifying the response to an intervention. However, findings regarding the utility of sensors in the framework of clinical trials were inconclusive. Finally, only a very small number of studies have investigated whether digital outcomes are subjectively meaningful and acceptable for people with MS. Although the results of these studies are overwhelmingly positive, future studies are needed to investigate people with MS perspectives on wearable devices, including concerns about data privacy and safety.

### Perspective and Recommendations

There are many barriers to the full clinical adoption of digital monitoring, including reproducibility of results, lack of external validation, and cost of digital devices. Our review demonstrated that there is substantial heterogeneity among the wearable sensor technologies used for the assessment of people with MS, not only regarding the number of devices but also in relation to the methodologies used in studying them. A large proportion of the devices, their potential benefits, and utility are still far from implementation in clinical routine, partly because of the lack of guidelines on how to use them. Further research is needed to accelerate the development of technology in the field of MS and to address unmet needs. This section provides recommendations on how to harmonize experimental designs and report studies using wearable sensor technologies to assess motor outcomes in people with MS (Table 3). This harmonization is necessary to compare different sensor technologies, facilitate reproducibility of studies, and establish benchmark technologies and data sets for validation of future studies and technologies.

**Table 3:** Recommendation for future investigations using sensor technologies in people with multiple sclerosis.

Recommendation	Remarks	Examples
Make code publicly available or usable	To reproduce results or use previous findings in a comparative study, it is pivotal to have access to the raw code or a binary variant thereof that was used to perform the experiments. Authors are encouraged to share their code, for example via platforms, such as GitHub, Inc (Microsoft Corp), or their binaries using container technologies like Docker.	GitHub [40,41,52,93,350] and Docker [351]
Make data available	In compliance with international data protection laws, data sources should be made accessible to bonafide researchers as far as possible. Numerous data repositories exist, which researchers can use to make their data (or parts thereof) accessible in accordance with data protection laws.	EUDAT^a^ [352], Zenodo [353], Dryad [354], IEEE^b^ DataPort [355], university repository [88,91], and journal supplement [92]
Provide sufficient details to reproduce study	A prerequisite of being able to replicate the results of any study is having access to the following details: study setting (eg, observational study, clinical trial, and secondary analysis), study population (eg, inclusion and exclusion criteria, demographics, and disease characteristics), sensor technology (eg, type and configuration of sensors, number of axes, mounting position, and recording frequency).	Detailed study protocol and statistical analysis plan
Use licenses for codes	Licenses protect the creators and the users of code by defining how and for what purposes the code can be reused. Several open source licenses exist, making it possible to satisfy the constraints of most authors, including companies that want to protect their intellectual property.	Apache license [40], BSD^c^ licenses, and GPL^d^ [52,93]
Use external validation for machine learning models	External validation is integral to determine the generalizability of machine learning models.	Publicly available data sets

^a^EUDAT: European Data Infrastructure.

^b^IEEE: Institute of Electrical and Electronics Engineers.

^c^BSD: Berkeley Software Distribution.

^d^GPL: General Public License.

Reproducibility is considered an essential cornerstone of scientific and technological progress [356]. Thus, it is important that studies provide sufficient details on the study setting (eg, observational study, clinical trial, and secondary analysis), study population (eg, inclusion and exclusion criteria, demographics, and disease characteristics), sensor technology (eg, type and configuration of sensors, number of axes, mounting position, and recording frequency), software used to extract raw data from the sensors, and algorithms used to define features and to perform the analysis. In light of this, authors are encouraged to make their codes publicly available on suitable platforms such as GitHub [357]. The development, adaptation, and extension of code are pivotal in promoting technological progress and assisting with the implementation of wearable sensor technologies in the clinical routine. Ideally, code sharing within reasonable bounds should become the default for publications in the field of MS and beyond.

Along with sharing codes, the authors would ideally make their data available. Although there are legitimate constraints with sharing data, authors should follow the FAIR principles and make their raw data (or metadata thereof) findable, accessible, interoperable, and reusable [358]. The FAIR guidelines provide guidance to authors on how to share their data while conserving the privacy of the patients and adhere to data safety and protection regulations (eg, Health Insurance Portability and Accountability Act of 1996 [359] and General Data Protection Regulations [360]). Sharing algorithmic details and raw sensor data allows for external validation and comparability of algorithms; thus, it is necessary to advance the field from the current state of independent feasibility studies to the next level of potential clinical use. Of the 308 included publications, only 16 have shared codes and data publicly [40-42,52,59,88-98]. Future studies could leverage shared data as an external validation set. Finally, we noticed that some studies reused data from previously published studies. Authors should declare the reuse of the study or patient populations by preregistering studies (eg, using the clinical trial platform [361]).

Guidelines for the use of wearable sensor technologies in clinical trials, their validation, and the reporting of their data have recently been developed by precompetitive collaborations between academia and industry [21,22], as well as regulatory agencies, such as Food and Drug Administration [7] and the European Medicines Agency [19,20]. However, these guidelines are not yet well known, and journals should recommend or even require their adaptation to disseminate these best practices. Furthermore, to the best of our knowledge, no guidelines exist specifically for the use of wearable sensor technologies in clinical routine, even though some clinical trial recommendations are applicable.

### Limitations of This Review

A limitation of this review is that the literature search was restricted to articles listed in PubMed, MEDLINE, Scopus, Embase, or Web of Science databases. Considering the pace at which the research in this area is moving forward, it is likely that the findings of the publications described in this review will be quickly complemented by further research. The literature search also excluded gray literature (eg, preprints, reports, and conference proceedings), whose importance is unknown to the topic, and thus might have introduced another source of search bias. There is also a probability of publication bias, which is likely to result from studies with more positive results being preferentially submitted and accepted for publication. Moreover, the heterogeneity in the outcome reporting and specifics of the sensor technologies used (eg, missing information on recording frequency, mounting position, and algorithms) did not allow us to perform a meta-analysis to aggregate the overall trend in performance among the sensor technologies. Another limitation is the reuse of study data, which has been reported in only a few publications. In some cases, a reuse of study data was suspected but was not specifically declared by the authors. Finally, a considerable number of studies (particularly in the laboratory setting group) did not provide any information on the disease course of the included participants. The lack of disease-specific information hampers the interpretation of study results and hinders comparisons across studies.

### Conclusions

Wearable sensor technologies have gained popularity in the field of MS over the last few decades, which is reflected by the large number of studies using them to objectively assess motor functions in people with MS in different settings (real-world and controlled laboratory settings) and clinical trials. Accelerometers, often in combination with gyroscopes and magnetometers, were the most commonly used sensors in the reviewed studies. In recent years, a surge in smartphone and smartwatch apps has been observed. A common denominator across the studies was that sensor technologies have the potential to reliably detect subtle changes in physical activity and other motor outcomes overlooked by conventional assessment methods, such as questionnaires and self-reported instruments. Thus, wearable sensor technologies constitute an intriguing tool to complement conventional clinical tests and self-reported outcomes. To adopt wearable sensor technologies in clinical routine and clinical trials, it is of utmost importance to establish guidelines for the use of sensor technologies and perform validation trials.

## Appendix

Multimedia Appendix 1 PRISMA (Preferred Reporting Items for Systematic Reviews and Meta-Analyses) checklist.

Multimedia Appendix 2 Local citation network of 308 included studies. Each node represents 1 study and the rows are ordered by publication year. Arrows between nodes indicate citations and each node’s size is proportional to its degree, that is, the sum of local incoming and outgoing citations [28].

Multimedia Appendix 3 Coauthorship network. Each node represents 1 author with at least 3 included studies, and the size of each node is proportional to the author’s number of included studies. Links between nodes indicate coauthorship, and each link’s width is proportional to the number of coauthored selected studies [28].

Multimedia Appendix 4 Distribution of the number of people with multiple sclerosis included in publications stratified by year of publication.

Multimedia Appendix 5 Detailed information on studies focusing on wearables in a real-world context. Compare Shiny app for the interactive version [28].

Multimedia Appendix 6 Detailed information on studies focusing on wearables in a laboratory context. Compare Shiny app for the interactive version [28].

Multimedia Appendix 7 Detailed information on studies focusing on wearables in a mixed context (ie, a combination of laboratory and real-world contexts). Compare Shiny app for the interactive version [28].

Multimedia Appendix 8 Number of publications per context over time.

Multimedia Appendix 9 Number of publications per number of wearables used and context studied.

Multimedia Appendix 10 Number of publications per wearable position over time. Publications may be counted multiple times.

Multimedia Appendix 11 Number of publications per domain studied. Publications may be counted multiple times.

Multimedia Appendix 12 Reporting quality assessment of included studies.

## Abbreviations

2MWT: 2-minute walking test
EDSS: Expanded Disability Status Scale
MS: multiple sclerosis
PDDS: Patient Determined Disease Steps
PRISMA: Preferred Reporting Items for Systematic Reviews and Meta-Analyses
T25FW: timed 25-foot walking test
TUG: timed up and go

## Data Availability

The GitHub repository including all code and data [27], the interactive Shiny app for exploring the results [28], and the interactive local citation network and coauthorship network of the included studies [29] are openly available.
