# Supplementary figures and images for: Wearable Sensor Technologies to Assess Motor Functions in People With Multiple Sclerosis: Systematic Scoping Review and Perspective
Source: J Med Internet Res. 2023 Jul 27;25:e44428. doi: 10.2196/44428 (PMC10415952; doi:10.2196/44428)

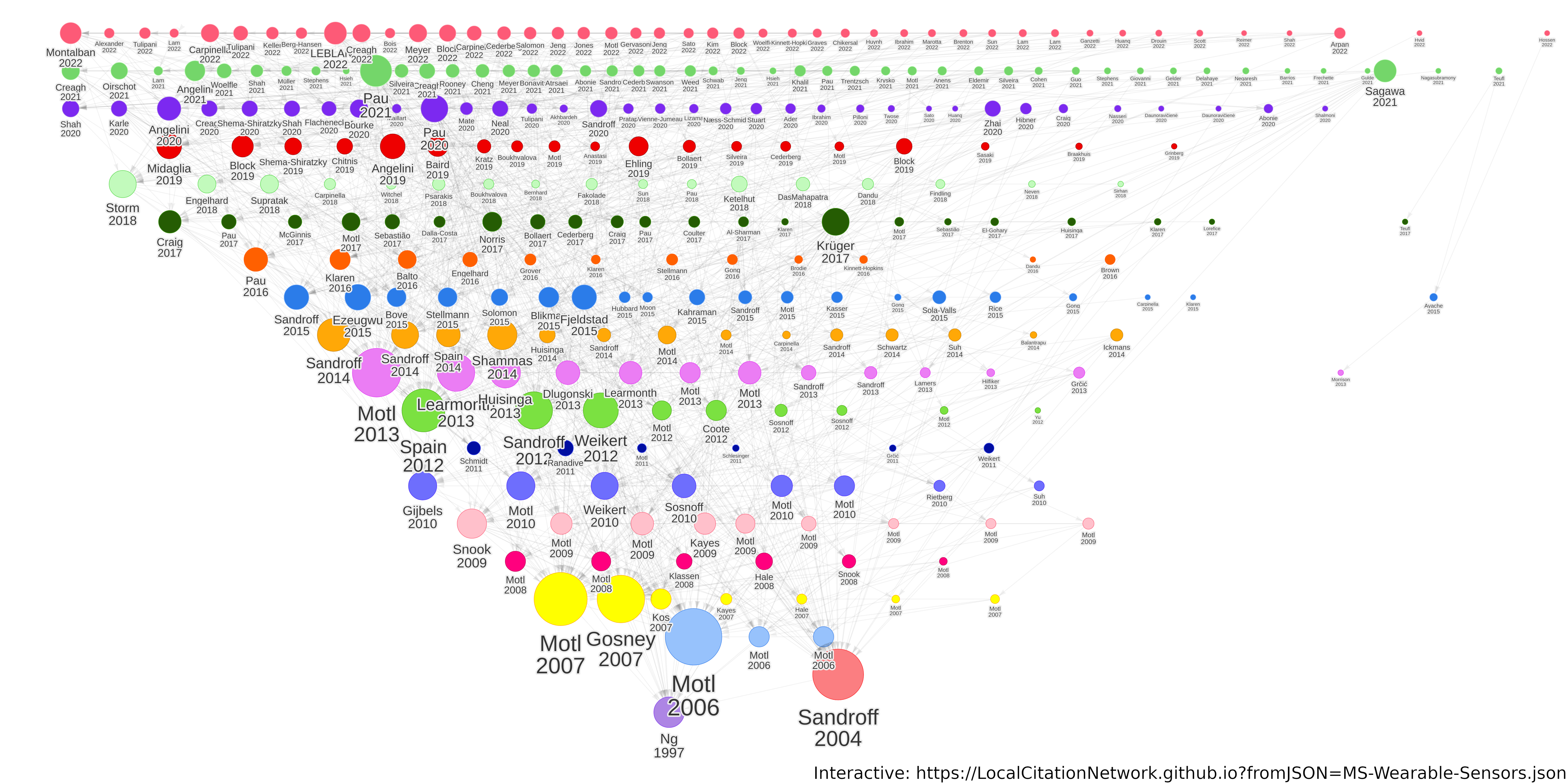

Supplement: Multimedia Appendix 2 [file jmir_v25i1e44428_app2.png]

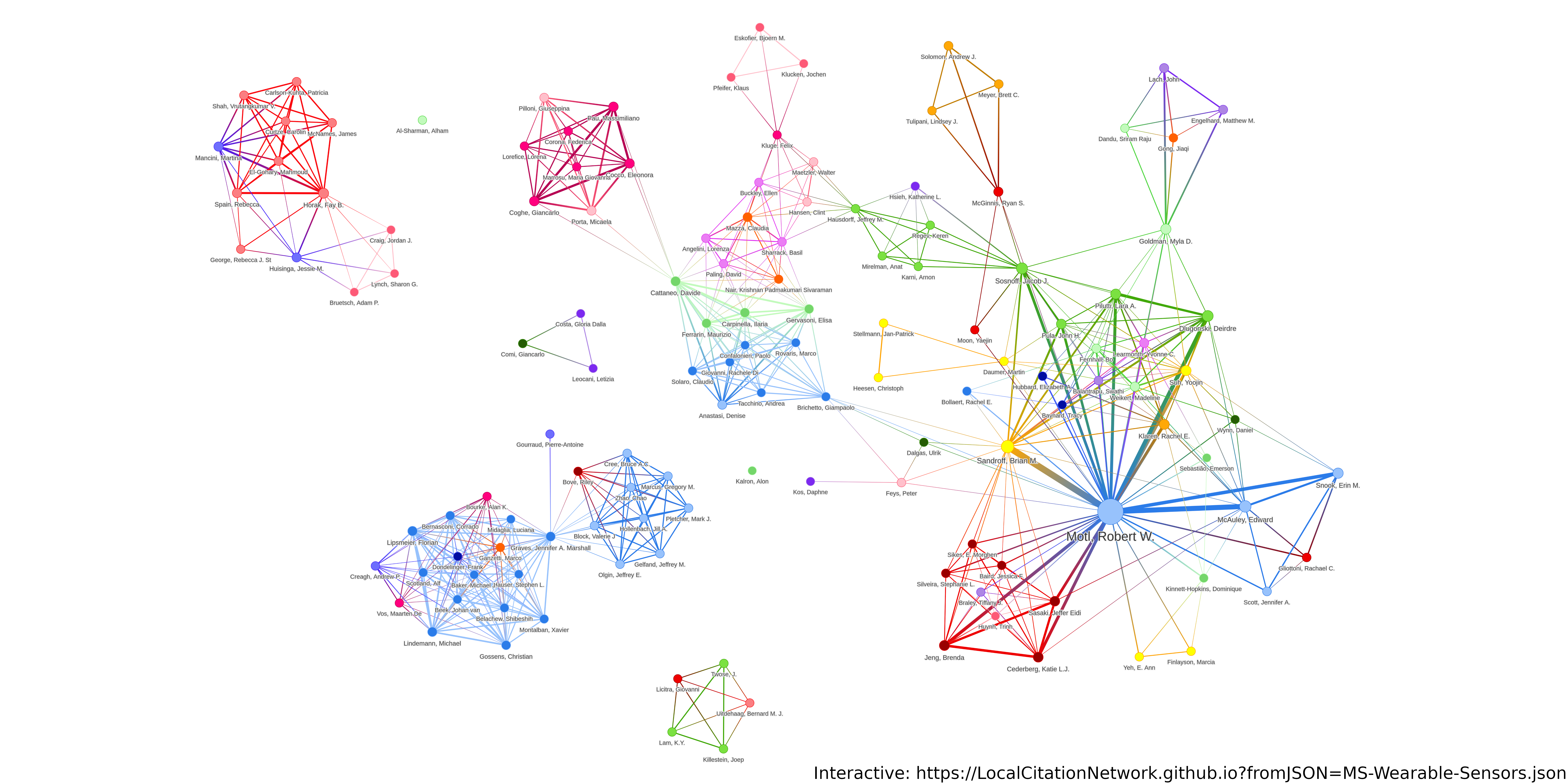

Supplement: Multimedia Appendix 3 [file jmir_v25i1e44428_app3.png]

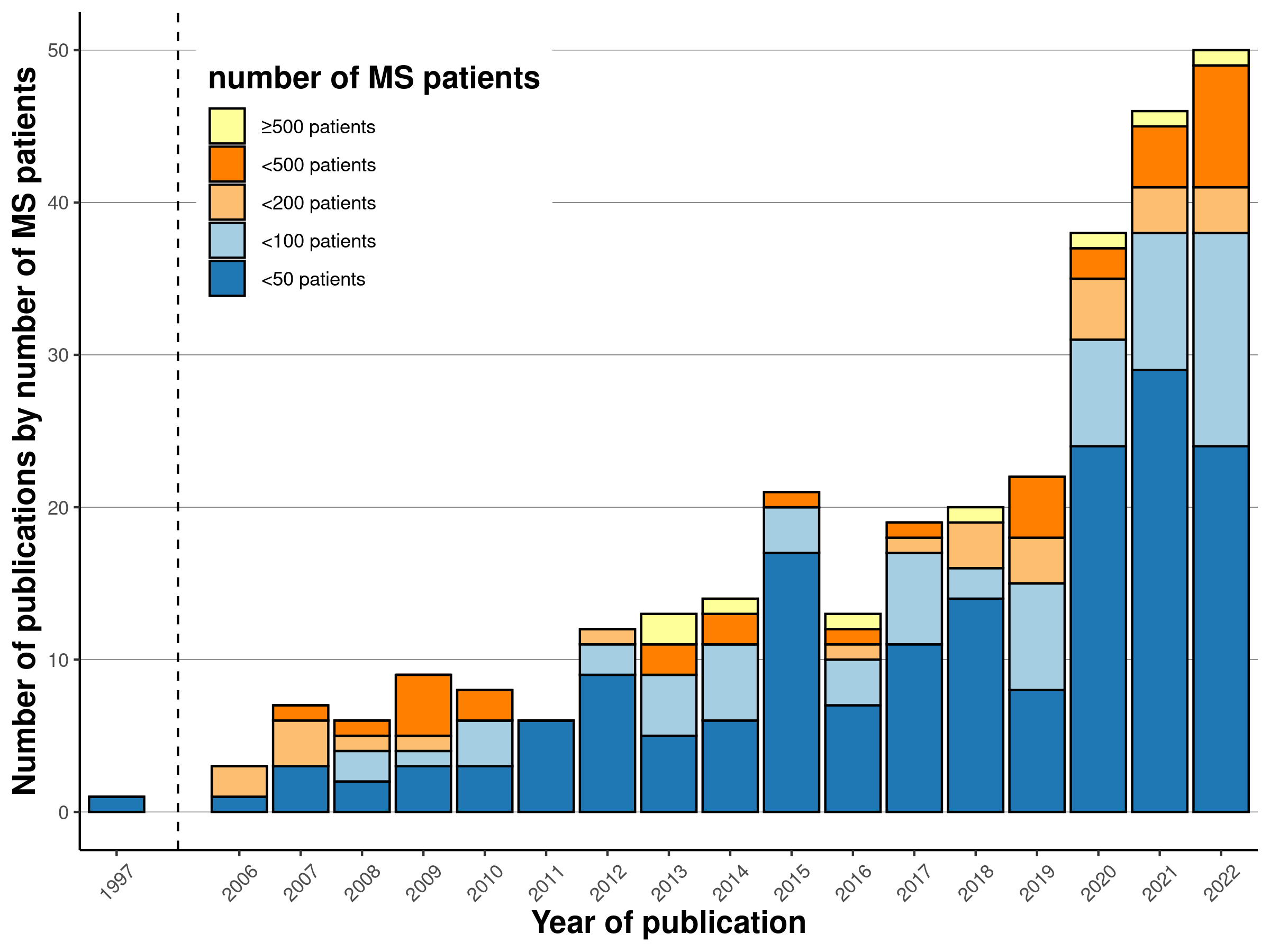

Supplement: Multimedia Appendix 4 [file jmir_v25i1e44428_app4.png]

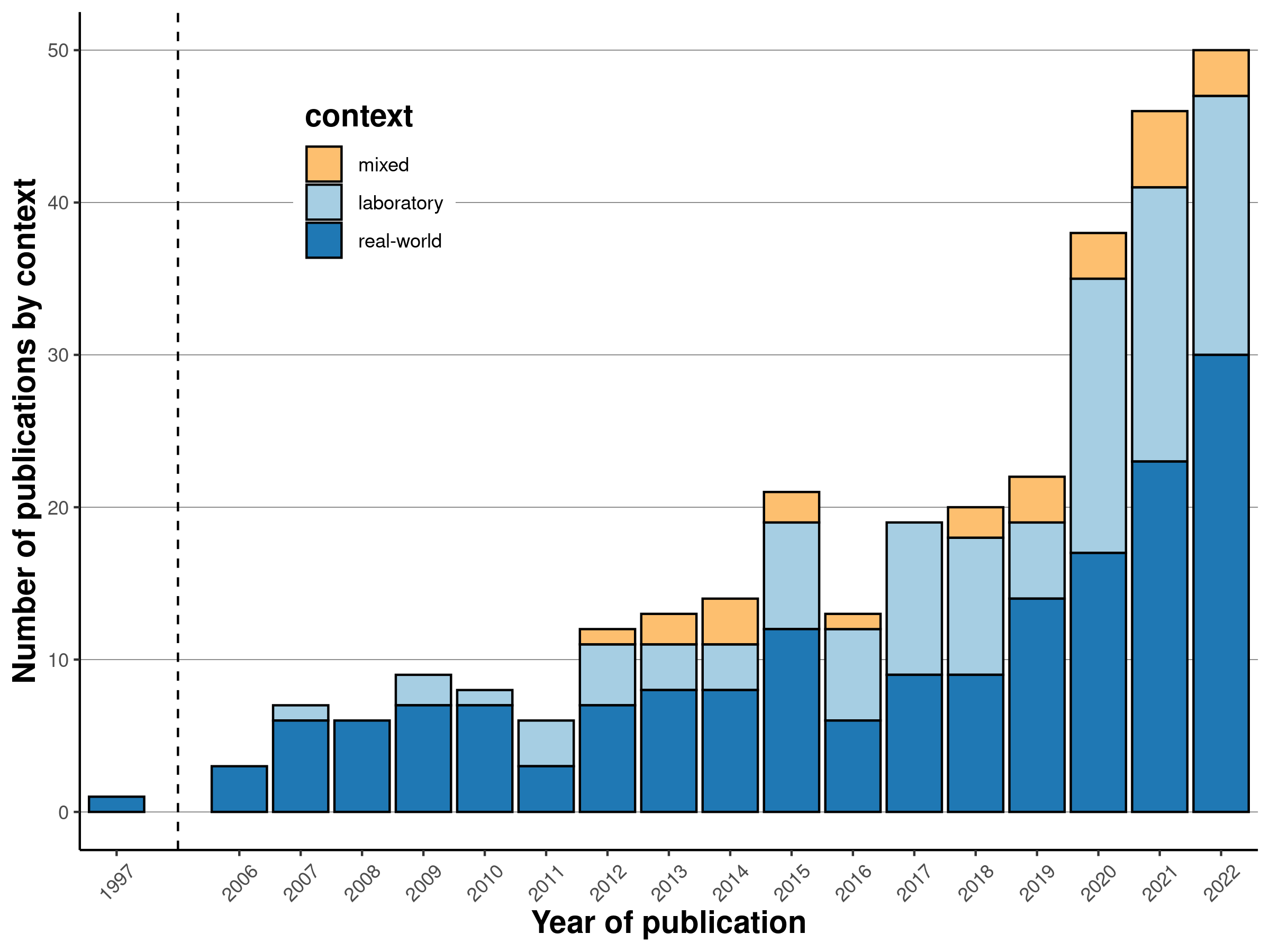

Supplement: Multimedia Appendix 8 [file jmir_v25i1e44428_app8.png]

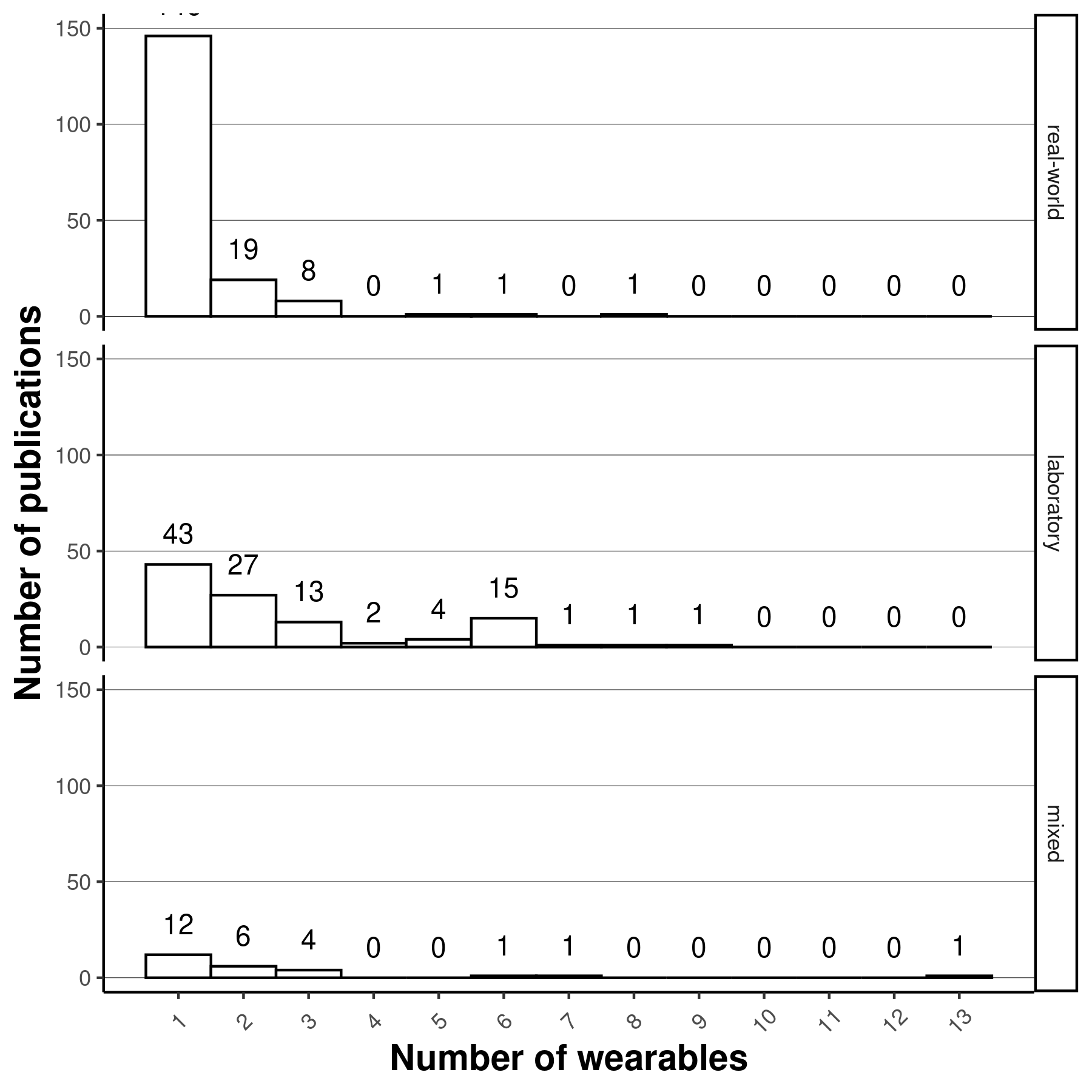

Supplement: Multimedia Appendix 9 [file jmir_v25i1e44428_app9.png]

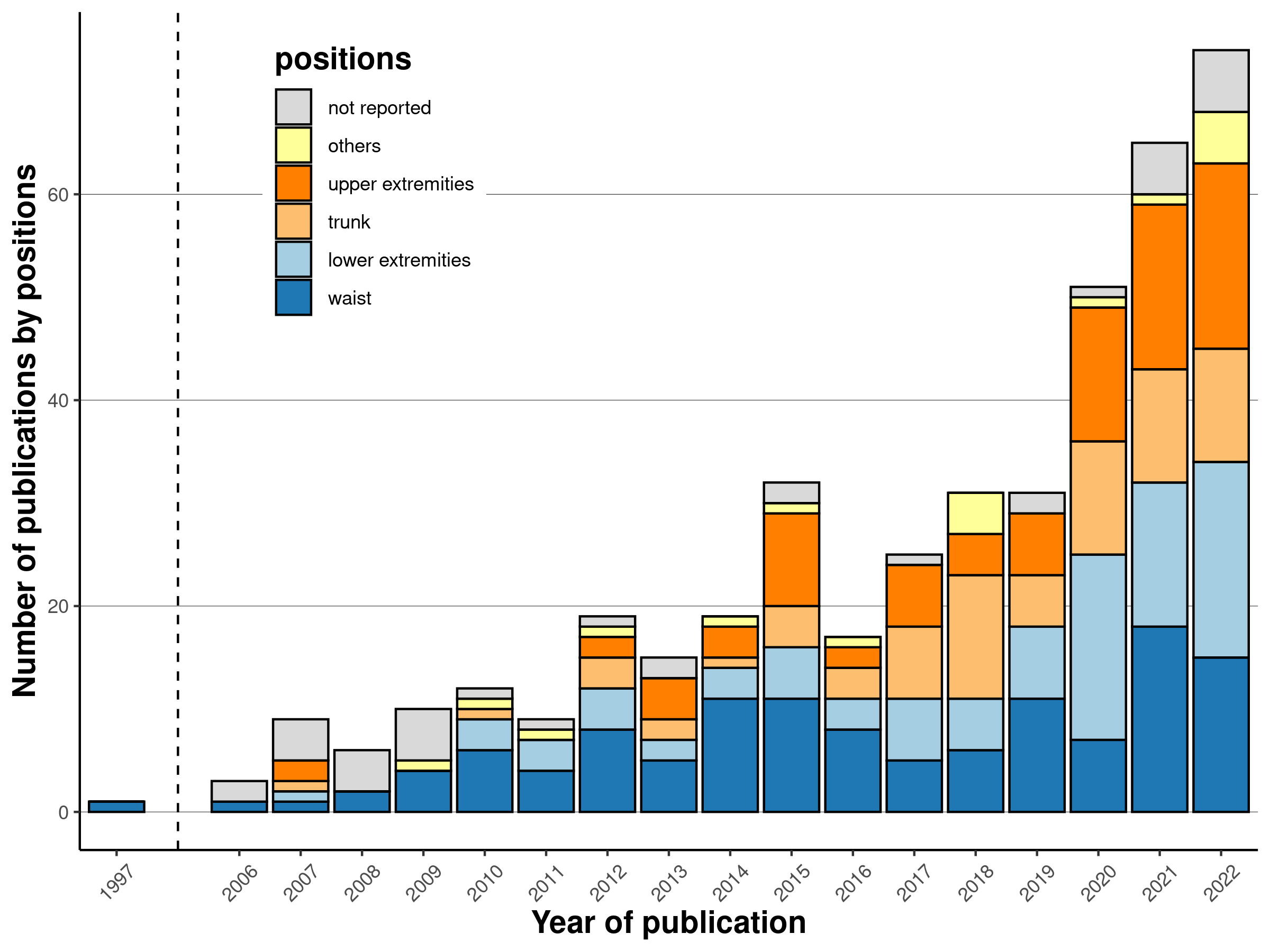

Supplement: Multimedia Appendix 10 [file jmir_v25i1e44428_app10.png]

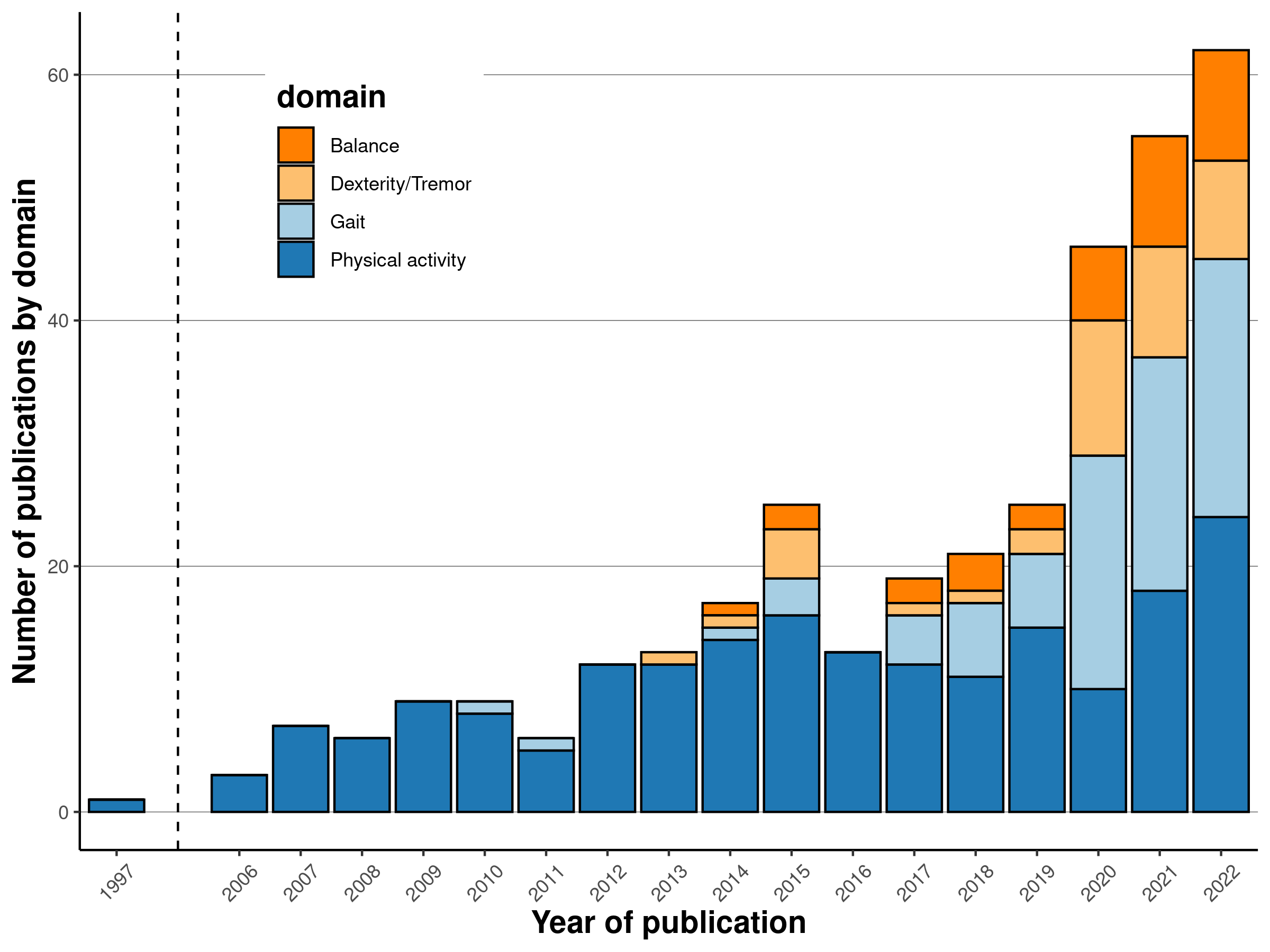

Supplement: Multimedia Appendix 11 [file jmir_v25i1e44428_app11.png]
