# Supplementary material for: Wearable Sensor Technologies to Assess Motor Functions in People With Multiple Sclerosis: Systematic Scoping Review and Perspective
Source: J Med Internet Res. 2023 Jul 27;25:e44428. doi: 10.2196/44428 (PMC10415952; doi:10.2196/44428)
Supplement: Multimedia Appendix 5 [file jmir_v25i1e44428_app5.docx]

**Multimedia Appendix 5. Detailed information on studies focusing on wearables in a real-world context. Time is expressed in years. Mean (SD) or median [IQR]. MS: multiple sclerosis, HC: healthy controls, RR: relapsing-remitting, PP: primary progressive, SP: secondary progressive, PDDS: patient determined disease steps, EDSS: expanded disability status scale, ns: non-significant, s: significant, ss: some significant, nt: not tested.**

Compare interactive web app: <https://lbourguignon.shinyapps.io/MS-Review/>

| **First author, year**  DOI | **MS Population of interest**  Sample size (% female)  Age  Type of MS | **Severity**  **Duration of disease** | **Comparator population type**  Sample size (% female)  Age | **Wearables**  Type of sensors  Number of axes Number of wearables  Positions | **Functional Domain**  Types or results reported (significance) |
| --- | --- | --- | --- | --- | --- |
| **Ng et al, 1997** [^10.1097/00005768-199704000-00014^](https://doi.org/10.1097/00005768-199704000-00014) | n=17 (65% female) age: 46 (6)  Type: RR: n=10, P: n=8 | **Severity:** EDSS: 3 (median), range: 1.5-6 | **healthy (sedentary)** n=15 (60% female) age: 44 (7) | **TriTrac R3D** accelerometer 3 axes 1 wearable(s) Position: waist | **Physical activity** Association with MS severity (ns) Association with other measure (ns) Group differences MS vs HC (ss) |
| **Motl et al, 2006** [^10.1080/09638280600551476^](https://doi.org/10.1080/09638280600551476) | n=30 (93% female) age: 42.3 (9.5), range: 27-61  Type: RR: n=26, PP: n=2, SP: n=2 | **Severity:** EDSS: 2.5 (median), range: 0-5 **Disease duration:** 6.2 (4.3) | none | **Yamax SW-200** mechanical pedometer 1 wearable(s) Position: waist  **ActiGraph 7164** accelerometer 1 axis 1 wearable(s) Position: waist | **Physical activity** Association with other measure (s) |
| **Motl et al, 2006** [^10.1002/NUR.20161^](https://doi.org/10.1002/NUR.20161) | n=196 (88% female) age: 46.1 (9.8)  Type: RR: n=174, PP: n=3, SP: n=19 | **Severity:** inclusion criteria: ambulatory with minimal assistance **Disease duration:** 9 (7.1) | none | **ActiGraph 7164** accelerometer 1 axis 1 wearable(s) Position: not reported | **Physical activity** Association with other measure (s) |
| **Motl et al, 2006** [^10.1207/S15324796ABM3202_13^](https://doi.org/10.1207/S15324796ABM3202_13) | n=196 (88% female) age: 46.1 (9.8)  Type: RR: n=174, PP: n=3, SP: n=19 | **Severity:** inclusion criteria: ambulatory with minimal assistance **Disease duration:** 9.0 (7.1) | none | **ActiGraph 7164** accelerometer 1 axis 1 wearable(s) Position: not reported | **Physical activity** Association with other measure (s) |
| **Gosney et al, 2007** [^10.1097/01.FCH.0000264411.20766.0C^](https://doi.org/10.1097/01.FCH.0000264411.20766.0C) | n=196 (88% female) age: 46.1 (9.8)  Type: RR: n=174, P: n=22 | **Disease duration:** 9.0 (7.1) | none | **Yamax SW-200** mechanical pedometer 1 axis 1 wearable(s) Position: not reported  **ActiGraph 7164** accelerometer 1 axis 1 wearable(s) Position: not reported | **Physical activity** Association with other measure (ns) |
| **Kayes et al, 2007** [^10.1177/0269215507075516^](https://doi.org/10.1177/0269215507075516) | n=30 (77% female) age: 54 (median), range: 27-76  Type: not reported: n=30 |  | none | **Actical** accelerometer 3 axes 1 wearable(s) (non-wheelchair user), 2 (wheelchair users) Position: waist, wrist (wheelchair users only) | **Physical activity** Test-retest reliability (ns) |
| **Kos et al, 2007** [^10.1080/07420520701282364^](https://doi.org/10.1080/07420520701282364) | n=19 (47% female) age: 47.2 (12.1)  Type: not reported: n=19 | **Severity:** EDSS: 5.5 [5-6] | **healthy** n=10 (60% female) age: 39.6 (12.3) | **ActiGraph** accelerometer 3 axes 2 wearable(s) Position: wrist, ankle | **Physical activity** Association with other measure (s) Group differences MS vs HC (ss) |
| **Motl et al, 2007** [^10.1037/0090-5550.52.2.143^](https://doi.org/10.1037/0090-5550.52.2.143) | n=292 (84% female) age: 48.0 (10.3), range: 20-69  Type: RR: n=246, PP: n=12, SP: n=34 | **Severity:** inclusion criteria: being able to walk **Disease duration:** 10.3 (7.9), range: 1-35 | none | **ActiGraph 7164** accelerometer 1 axis 1 wearable(s) Position: not reported | **Physical activity** Association with MS severity (ss) Association with other measure (ss) |
| **Motl et al, 2007** [^10.1037/0090-5550.52.4.463^](https://doi.org/10.1037/0090-5550.52.4.463) | n=133 (78% female) age: 51.1 (11.4)  Type: RR: n=86, PP: n=6, SP: n=41 | **Disease duration:** 12.0 (9.0) | none | **ActiGraph 7164** accelerometer 1 axis 1 wearable(s) Position: not reported | **Physical activity** Association with other measure (s) |
| **Motl et al, 2007** [^10.1123/APAQ.24.3.245^](https://doi.org/10.1123/APAQ.24.3.245) | n=193 (88% female) age: 46.2 (9.7)  Type: RR: n=171, PP: n=3, SP: n=19 | **Severity:** inclusion criteria: ambulatory with minimal assistance **Disease duration:** 9.0 (7.1) | none | **Yamax SW-200** mechanical pedometer 1 axis 1 wearable(s) Position: not reported  **ActiGraph 7164** accelerometer 1 axis 1 wearable(s) Position: not reported | **Physical activity** Test-retest reliability (ns) |
| **Hale et al, 2008** [^10.1016/J.APMR.2008.02.027^](https://doi.org/10.1016/J.APMR.2008.02.027) | n=11 (73% female) age: 63.7 (15.5), range: 28-91  Type: not reported: n=11 | **Severity:** Rivermead Mobility Index: 12.4 (2.2), range: 8-15 | **healthy (sedentary)** n=9 (89% female) age: 51 (18.1), range: 28-76 | **TriTrac RT3** accelerometer 3 axes 1 wearable(s) Position: waist | **Physical activity** Test-retest reliability (s) Group differences MS vs HC (ns) Group differences MS vs other diseases (ns) Subjective participant acceptability (ns) |
| **Klassen et al, 2007** [^10.1177/0269215507082740^](https://doi.org/10.1177/0269215507082740) | n=30 (63% female) age: inactive: 47.7 (8.6) mod active: 50.6 (4.5) active: 44.4 (7.8)  Type: not reported: n=30 | **Severity:** EDSS: range: 1-6 | **healthy** n=9 (89% female) age: 41.6 (4.4), range: 37-51 | **TriTrac RT3** accelerometer 3 axes 1 wearable(s) Position: waist | **Physical activity** Association with MS severity (s) Association with other measure (s) Group differences MS vs HC (ss) Group differences MS vs MS (ss) |
| **Motl et al, 2008** [^10.1007/S12160-008-9049-4^](https://doi.org/10.1007/S12160-008-9049-4) | n=292 (84% female) age: 48.0 (10.3)  Type: RR: n=246, PP: n=12, SP: n=34 | **Severity:** inclusion criteria: being ambulatory with minimal assistance **Disease duration:** 10.3 (7.9) | none | **ActiGraph 7164** accelerometer 1 axis 1 wearable(s) Position: not reported | **Physical activity** Association with other measure (s) |
| **Motl et al, 2008** [^10.1016/J.JNS.2007.11.003^](https://doi.org/10.1016/J.JNS.2007.11.003) | n=133 (78% female) age: 51.1 (11.1)  Type: RR: n=85, PP: n=6, SP: n=52 | **Severity:** EDSS: 4.9 (2.0) **Disease duration:** 12.0 (9.0) | none | **ActiGraph 7164** accelerometer 1 axis 1 wearable(s) Position: not reported | **Physical activity** Association with other measure (ns) |
| **Motl et al, 2008** [^10.1097/NMD.0B013E318177351B^](https://doi.org/10.1097/NMD.0B013E318177351B) | n=80 (81% female) age: 49.0 (11.4)  Type: RR: n=65, PP: n=1, SP: n=14 | **Severity:** EDSS: 4 (mean), range: 1-6.5 **Disease duration:** 10.1 (7.9) | none | **ActiGraph 7164** accelerometer 1 axis 1 wearable(s) Position: not reported | **Physical activity** Association with MS severity (s) Association with other measure (ss) |
| **Snook et al, 2008** [^10.1016/J.JPAINSYMMAN.2007.09.007^](https://doi.org/10.1016/J.JPAINSYMMAN.2007.09.007) | n=80 (81% female) age: 49.0 (11.4)  Type: RR: n=62, PP: n=1, SP: n=15, benign: n=2 | **Severity:** EDSS: 3.9 (1.8) **Disease duration:** 10.1 (7.9) | none | **ActiGraph 7164** accelerometer 1 axis 1 wearable(s) Position: not reported | **Physical activity** Association with other measure (s) |
| **Motl et al, 2009** [^10.1037/A0015770^](https://doi.org/10.1037/A0015770) | n=292 (84% female) age: 48.0 (10.3), range: 20-69  Type: RR: n=246, PP: n=12, SP: n=34 | **Severity:** PDDS: 3.0 (median), range: 0-6 **Disease duration:** 10.3 (7.9), range: 1-35 | none | **ActiGraph 7164** accelerometer 1 axis 1 wearable(s) Position: waist | **Physical activity** Association with other measure (s) Responsiveness to change (ss) |
| **Motl et al, 2009** [^10.1037/A0015985^](https://doi.org/10.1037/A0015985) | n=292 (84% female) age: 48.0 (10.3), range: 20-69  Type: RR: n=246, PP: n=12, SP: n=34 | **Severity:** PDDS: 3.0 (median), range: 0-6 **Disease duration:** 10.3 (7.9), range: 1-35 | none | **ActiGraph 7164** accelerometer 1 axis 1 wearable(s) Position: not reported | **Physical activity** Responsiveness to change (ss) |
| **Motl et al, 2009** [^10.1016/J.JPAINSYMMAN.2008.08.004^](https://doi.org/10.1016/J.JPAINSYMMAN.2008.08.004) | n=292 (84% female) age: 48.0 (10.3)  Type: RR: n=239, PP: n=12, SP: n=34, benign: n=7 | **Disease duration:** 10.3 (7.9) | none | **ActiGraph 7164** accelerometer 1 axis 1 wearable(s) Position: not reported | **Physical activity** Association with other measure (s) |
| **Motl et al, 2009** [^10.1097/MRR.0B013E328325A5ED^](https://doi.org/10.1097/MRR.0B013E328325A5ED) | n=16 (88% female) age: 43.4 (8.7)  Type: RR: n=16 |  | none | **ActiGraph 7164** accelerometer 1 axis 1 wearable(s) Position: not reported | **Physical activity** Association with other measure (ss) Responsiveness to change (ns) |
| **Motl et al, 2009** [^10.1080/13548500802241902^](https://doi.org/10.1080/13548500802241902) | n=292 (84% female) age: 48.0 (10.3), range: 20-69  Type: RR: n=246, PP: n=12, SP: n=34 | **Disease duration:** 10.3 (7.9), range: 1-35 | none | **ActiGraph 7164** accelerometer 1 axis 1 wearable(s) Position: not reported | **Physical activity** Association with MS severity (s) Association with other measure (ss) |
| **Motl et al, 2009** [^10.1016/J.JNS.2009.06.015^](https://doi.org/10.1016/J.JNS.2009.06.015) | n=133 age: NA  Type: RR: n=82, P: n=51 | **Severity:** EDSS: 5.5 (median), range: 1-8.5 **Disease duration:** 12 (9) | none | **ActiGraph 7164** accelerometer 1 axis 1 wearable(s) Position: not reported | **Physical activity** Association with other measure (ns) |
| **Snook et al, 2009** [^10.1177/0269215508101757^](https://doi.org/10.1177/0269215508101757) | n=74 (82% female) age: 49.0 (11.6)  Type: RR: n=58, PP: n=1, SP: n=13, benign: n=2 | **Severity:** EDSS: 3.9 (1.8) **Disease duration:** 10.1 (8.0) | none | **ActiGraph 7164** accelerometer 1 axis 1 wearable(s) Position: waist | **Physical activity** Association with other measure (ss) Group differences MS vs MS (s) |
| **Gijbels et al, 2010** [^10.1177/1352458510361357^](https://doi.org/10.1177/1352458510361357) | n=50 (66% female) age: 49 (10), range: 25-66  Type: RR: n=23, PP: n=14, SP: n=13 | **Severity:** EDSS: 4.5 (1.2), range: 1.5-6.5 | none | **SAM 2D** accelerometer 2 axes 1 wearable(s) Position: ankle | **Physical activity** Association with MS severity (ss) Association with other measure (ss) |
| **Motl et al, 2010** [^10.1016/J.APMR.2010.08.011^](https://doi.org/10.1016/J.APMR.2010.08.011) | n=26 (85% female) age: 43.1 (11.9)  Type: RR: n=26 | **Severity:** PDDS: 1.8 (1.6) **Disease duration:** 11.6 (8.4) | none | **ActiGraph 7164** accelerometer 1 axis 1 wearable(s) Position: waist  **Cosmed K4b2** others (VO2) 1 wearable(s) Position: others (head) | **Physical activity** Association with MS severity (s) Association with other measure (s) |
| **Motl et al, 2010** [^10.1097/NMD.0B013E3181D14131^](https://doi.org/10.1097/NMD.0B013E3181D14131) | n=269 (83% female) age: 45.9 (9.6)  Type: RR: n=269 | **Severity:** PDDS: 2 (median), range: 0-6 **Disease duration:** 13.3 (9.2) | none | **ActiGraph 7164** accelerometer 1 axis 1 wearable(s) Position: waist | **Physical activity** Association with MS severity (ns) Association with other measure (ns) |
| **Rietberg et al, 2010** [^10.1016/J.APMR.2010.07.018^](https://doi.org/10.1016/J.APMR.2010.07.018) | n=43 (70% female) age: 48.7 (7.0)  Type: RR: n=26, PP: n=7, SP: n=10 | **Severity:** EDSS: 3.5 [2.5], range: 1-6 **Disease duration:** 14.3 (9.2), range: 2-51 | none | **Vitaport Step Watch** accelerometer 2 axes 5 wearable(s) Position: sternum, waist, upper leg | **Physical activity** Test-retest reliability (s) |
| **Sosnoff et al, 2010** [^10.1177/1352458510373111^](https://doi.org/10.1177/1352458510373111) | n=70 (80% female) age: Age by self-reported disability status: mild: 47.4 (10.1), moderate: 49.4 (13.1), severe: 53.2 (10.3)  Type: RR: n=56, PP: n=1, SP: n=13 | **Severity:** SR-EDSS: mild: 2.1 (0.77), moderate: 5.05 (0.53), severe: 6.02 (0.12) **Disease duration:** Self-reported disability status: mild: 10.3 (8.4), moderate: 10.7 (9.6), severe: 11.5 (5.1) | none | **ActiGraph 7164** accelerometer 1 axis 1 wearable(s) Position: waist | **Physical activity** Association with other measure (s) Group differences MS vs MS (ss) |
| **Suh et al, 2010** [^10.1016/J.DHJO.2009.09.002^](https://doi.org/10.1016/J.DHJO.2009.09.002) | n=96 (78% female) age: 42.8 (10.2), range: 20-64  Type: RR: n=91, PP: n=2, SP: n=3 | **Disease duration:** 3.0 (1.5), range: 0.5-5 | none | **ActiGraph 7164** accelerometer 1 axis 1 wearable(s) Position: not reported | **Physical activity** Association with MS severity (s) Association with other measure (ss) |
| **Weikert et al, 2010** [^10.1016/J.JNS.2009.12.021^](https://doi.org/10.1016/J.JNS.2009.12.021) | n=269 (83% female) age: 45.9 (9.6)  Type: RR: n=269 | **Severity:** PDDS: 2 (median), range: 0-6 **Disease duration:** 13.3 (9.2) | none | **ActiGraph 7164** accelerometer 1 axis 1 wearable(s) Position: waist | **Physical activity** Association with MS severity (ns) Association with other measure (ns) |
| **Grčić et al, 2011** [^10.12659/MSM.882130^](https://doi.org/10.12659/MSM.882130) | n=49 (80% female) age: 35 (median), range: 18-56  Type: RR: n=49 | **Severity:** EDSS: 3.0 (median), range: 1.5-6.0 **Disease duration:** 8 (median), range: 1.3-27 | none | **StepWatch Activity Monitor** accelerometer 3 axes 1 wearable(s) Position: ankle | **Physical activity** Association with MS severity (ns) Responsiveness to change (ss) |
| **Motl et al, 2011** [^10.1080/08964289.2011.636769^](https://doi.org/10.1080/08964289.2011.636769) | n=18 (89% female) age: 45.1 (9.5)  Type: RR: n=18 | **Severity:** PDDS: 1.0 (median), range: 0-4 **Disease duration:** 7.3 (5.0) | none | **ActiGraph 7164** accelerometer 1 axis 1 wearable(s) Position: waist  **Yamax SW-201** mechanical pedometer 1 wearable(s) Position: not reported | **Physical activity** Responsiveness to change (ss) Responsiveness to intervention (ss) |
| **Schlesinger et al, 2011** [^10.1055/S-0031-1271750^](https://doi.org/10.1055/S-0031-1271750) | n=16 (50% female) age: 47.56 (7.78), range: 39-70  Type: not reported: n=16 | **Severity:** EDSS: 3.56 (1.05), 3.5 (median), range: 2-5.5 | **healthy** n=26 (42% female) age: 37.3 (12.9) | **Actibelt** accelerometer 3 axes 1 wearable(s) Position: waist | **Physical activity** Association with MS severity (ns) Group differences MS vs HC (s) |
| **Motl et al, 2012** [^10.1037/A0025965^](https://doi.org/10.1037/A0025965) | n=18 (83% female) age: 45.4 (11.2)  Type: RR: n=18 | **Disease duration:** 7.7 (5.5) | **MS patients** n=20 (80% female) age: 48.3 (9.8) Type: RR: n=18, not reported: n=2 Severity of MS: not reported Disease duration: 9.9 (8.9) | **Yamax SW-200** mechanical pedometer 1 axis 1 wearable(s) Position: waist  **ActiGraph 7164** accelerometer 1 axis 1 wearable(s) Position: waist  **Omron HJ-720ITC** accelerometer 1 axis 1 wearable(s) Position: waist | **Physical activity** Responsiveness to intervention (s) |
| **Ranadive et al, 2012** [^10.1249/MSS.0B013E31822D7997^](https://doi.org/10.1249/MSS.0B013E31822D7997) | n=33 (82% female) age: 47.0 (1.83)  Type: RR: n=29, PP: n=1, SP: n=3 | **Severity:** PDSS: 2 (median), range: 0-6 **Disease duration:** 9.2 (6.7) | **healthy** n=33 (82% female) age: 47.0 (1.97) | **ActiGraph 7164** accelerometer 1 axis 1 wearable(s) Position: not reported | **Physical activity** Association with other measure (ss) Group differences MS vs HC (s) |
| **Sandroff et al, 2012** [^10.1111/J.1600-0404.2011.01634.X^](https://doi.org/10.1111/J.1600-0404.2011.01634.X) | n=77 (86% female) age: 47.3 (9.7)  Type: RR: n=66, not reported: n=11 | **Severity:** PDSS: 1 (median), range: 0-6 **Disease duration:** 10.1 (7.3) | **healthy** n=66 (85% female) age: 47.0 (10.5) | **ActiGraph 7164** accelerometer 1 axis 1 wearable(s) Position: waist | **Physical activity** Association with other measure (ss) Group differences MS vs HC (s) |
| **Sosnoff et al, 2012** [^10.1155/2012/315620^](https://doi.org/10.1155/2012/315620) | n=37 (76% female) age: 53.4 (10.0)  Type: not reported: n=37 | **Severity:** EDSS: 5.0 [4] **Disease duration:** 14.6 (10.6) | **MS patients (non-fallers)** n=38 (82% female) age: 50.1 (13.9) Severity: EDSS: 3.0 [2.0] Disease duration: 11.0 (9.6) | **ActiGraph GT3X** accelerometer 3 axes 1 wearable(s) Position: waist | **Physical activity** Association with MS severity (s) Association with other measure (ns) Group differences MS vs MS (s) |
| **Sosnoff et al, 2012** [^10.1682/JRRD.2011.11.0218^](https://doi.org/10.1682/JRRD.2011.11.0218) | n=22 (77% female) age: 46.9 (11.7), range 23-64  Type: not reported: n=22 | **Severity:** EDSS: 3.0 [2.5] range: 1-6 **Disease duration:** 11.0 (6.9), range: 1-23 | none | **ActiGraph GT3X** accelerometer 3 axes 1 wearable(s) Position: waist  **IDEEA system** accelerometer 1 axis 5 wearable(s) Position: sternum, waist, upper leg, foot | **Physical activity** Association with MS severity (s) Association with other measure (ss) |
| **Weikert et al, 2012** [^10.1016/J.MEDENGPHY.2011.09.005^](https://doi.org/10.1016/J.MEDENGPHY.2011.09.005) | n=33 (82% female) age: 47.5 (10.6)  Type: RR: n=28, not reported: n=5 | **Severity:** PDDS: 2 (median), range: 0-6 **Disease duration:** 9.2 (6.7) | **healthy** n=33 (82% female) age: 47.7 (11.3) | **ActiGraph 7164** accelerometer 1 axis 1 wearable(s) Position: waist | **Physical activity** Association with other measure (ss) Group differences MS vs HC (ns) |
| **Yu et al, 2012** [^10.1088/0967-3334/33/12/2033^](https://doi.org/10.1088/0967-3334/33/12/2033) | n=17 age: range: 20-65 [inclusion criteria]  Type: not reported: n=17 | **Severity:** inclusion criteria: EDSS<5.5 | **healthy** n=9 (sex-matched to MS patients) age: not reported | **FAMOS prototype** accelerometer, others (ECG, sEMG, temperature) 1 axis 8 wearable(s) Position: upper back, upper leg, lower leg, ankle | **Physical activity** Group differences MS vs HC (ss) |
| **Dlugonski et al, 2013** [^10.1016/J.APMR.2012.12.014^](https://doi.org/10.1016/J.APMR.2012.12.014) | n=645 (85% female) age: 46.3 (10.6)  Type: RR: n=575, P: n=63, not reported: n=7 | **Severity:** PDDS<=2: n=255 PDDS=>3: n=194 **Disease duration:** <10 years: n=395,  10 years: n=250 | none | **Yamax SW-200** mechanical pedometer 1 axis 1 wearable(s) Position: not reported  **ActiGraph 7164** accelerometer 1 axis 1 wearable(s) Position: not reported | **Physical activity** Association with MS severity (s) |
| **Grčić et al, 2013** [^10.1007/S13760-013-0187-5^](https://doi.org/10.1007/S13760-013-0187-5) | n=82 (74% female) age: 37.8 (9.9)  Type: not reported: n=82 | **Severity:** EDSS: 2.5 (median), range: 0-6.5 **Disease duration:** 9.0 (6.3) | none | **StepWatch Activity Monitor** accelerometer 3 axes 1 wearable(s) Position: ankle | **Physical activity** Association with MS severity (s) Association with other measure (s) |
| **Learmonth et al, 2013** [^10.1177/1352458513483890^](https://doi.org/10.1177/1352458513483890) | n=82 (76% female) age: 49.2 (9.0)  Type: RR: n=65, PP: n=7, SP: n=10 | **Severity:** SR-EDSS: 3.5 (2.0), 3.5 [3.5], range: 0-6.5, PDDS: 2.6 (1.8), 3 [3], range: 0-6 **Disease duration:** 11.8 (8.2), range: 0.5-32 | none | **ActiGraph GT3X** accelerometer 3 axes 1 wearable(s) Position: waist | **Physical activity** Test-retest reliability (s) |
| **Learmonth et al, 2013** [^10.1186/1471-2377-13-37^](https://doi.org/10.1186/1471-2377-13-37) | n=96 (80% female) age: 53.5 (14), range: 30-78  Type: RR: n=79, not reported: n=17 | **Severity:** EDDS: 4.5 [3.0], range: 2-6.5, PDDS: 3.0 [3.0], range: 0-6 **Disease duration:** 9 (12), range: 1-43 | none | **ActiGraph GT3X** accelerometer 3 axes 1 wearable(s) Position: waist | **Physical activity** Association with MS severity (s) |
| **Motl et al, 2013** [^10.2522/PTJ.20120479^](https://doi.org/10.2522/PTJ.20120479) | n=269 (83% female) age: 45.9 (9.6)  Type: RR: n=269 | **Severity:** PDDS: 2 [3] **Disease duration:** 8.8 (7.0) | none | **ActiGraph 7164** accelerometer 1 axis 1 wearable(s) Position: waist | **Physical activity** Responsiveness to change (s) |
| **Motl et al, 2013** [^10.1111/ANE.12036^](https://doi.org/10.1111/ANE.12036) | n=256 (81% female) age: 49.5 (10.2)  Type: RR: n=215, PP: n=13, SP: n=24, not reported: n=4 | **Severity:** EDSS: 4.0 [3.5], range: 0-6.5 **Disease duration:** 11.3 (8.7) | none | **ActiGraph GT3X** accelerometer 3 axes 1 wearable(s) Position: wrist | **Physical activity** Association with MS severity (s) Association with other measure (s) |
| **Motl et al, 2013** [^10.1371/JOURNAL.PONE.0073247^](https://doi.org/10.1371/JOURNAL.PONE.0073247) | n=786 (85% female) age: 47.3 (10.5)  Type: RR: n=705; not reported: n=81 | **Severity:** PDDS: 2.0 [2.0] **Disease duration:** 9.9 (8.0) | **healthy** n=157 (91% female) age: 43.4 (9.8) | **Yamax SW-200** mechanical pedometer 1 axis 1 wearable(s) Position: not reported  **ActiGraph 7164** accelerometer 1 axis 1 wearable(s) Position: not reported | **Physical activity** Association with MS severity (s) Group differences MS vs HC (s) |
| **Sandroff et al, 2013** [^10.1016/J.MHPA.2013.08.001^](https://doi.org/10.1016/J.MHPA.2013.08.001) | n=82 (76% female) age: 49.0 (9.1)  Type: RR: n=65, PP: n=3, SP: n=10, not reported: n=4 | **Severity:** SR-EDSS: 3.5 (median), range: 0-6.5 **Disease duration:** 11.8 (8.2) | none | **ActiGraph GT3X** accelerometer 3 axes 1 wearable(s) Position: waist | **Physical activity** Association with other measure (s) |
| **Balantrapu et al, 2014** [^10.1155/2014/649390^](https://doi.org/10.1155/2014/649390) | n=44 (73% female) age: 52.8 (7.6)  Type: RR: n=36, not reported: n=8 | **Severity:** EDSS: 6 (median), range: 2.5-6.5 **Disease duration:** 10.3 (8.4), range: 0-39 | **MS patients (without spasticity)** n=40 (90% female) age: 47.7 (11.1) Type: RR: n=37 Severity: EDDS: 3.5 [2.0-6.5] Disease duration: 11.5 (9.7) | **ActiGraph GT3X** accelerometer 3 axes 1 wearable(s) Position: waist | **Physical activity, Dexterity/Tremor** Group differences MS vs MS (s) |
| **Ickmans et al, 2014** [^10.1016/J.CLINEURO.2014.04.021^](https://doi.org/10.1016/J.CLINEURO.2014.04.021) | n=19 (68% female) age: 39.74 (10.67)  Type: not reported: n=19 | **Severity:** EDSS: 1.64 (1.02) **Disease duration:** 83.52 (68.50) months | **healthy** n=32 (69% female) age: 39.3 (13.9) | **Actical** accelerometer 3 axes 1 wearable(s) Position: wrist | **Physical activity** Group differences MS vs HC (s) |
| **Motl et al, 2014** [^10.1080/08964289.2013.821966^](https://doi.org/10.1080/08964289.2013.821966) | n=567 (84% female) age: 47.0 (10.0), range: 19-84  Type: RR: n=519, PP: n=12, SP: n=36 | **Severity:** PDDS: 2.0 (median), range: 0-6 **Disease duration:** 9.6 (7.5), range: 1-37 | none | **ActiGraph 7164** accelerometer 1 axis 1 wearable(s) Position: waist | **Physical activity** Test-retest reliability (s) |
| **Sandroff et al, 2014** [^10.1016/J.MSARD.2013.06.014^](https://doi.org/10.1016/J.MSARD.2013.06.014) | n=84 (79% female) age: 51.8 (11.3)  Type: RR: n=69, PP: n=4, SP: n=7, not reported: n=4 | **Severity:** EDDS: 4.5 (median), range: 2.0-6.5 **Disease duration:** 10.8 (9.3) | none | **ActiGraph GT3X** accelerometer 3 axes 1 wearable(s) Position: waist | **Physical activity** Association with other measure (s) |
| **Sandroff et al, 2014** [^10.1371/JOURNAL.PONE.0093511^](https://doi.org/10.1371/JOURNAL.PONE.0093511) | n=63 (76% female) age: 50.68 (9.22)  Type: RR: n=50, not reported: n=13 | **Severity:** EDSS: 4.0 (median), range: 1.0-6.5 **Disease duration:** 12.83 (8.50) | none | **ActiGraph GT3X** accelerometer 3 axes 1 wearable(s) Position: waist  **StepWatch Activity Monitor** mechanical pedometer 1 wearable(s) Position: ankle | **Physical activity** Group differences MS vs MS (s) |
| **Schwartzt et al, 2014** [^10.1016/J.JNS.2014.10.021^](https://doi.org/10.1016/J.JNS.2014.10.021) | n=269 (83% female) age: 45.85 (9.73)  Type: RR: n=269 | **Severity:** PDDS: 2 [3], MSWS: 36.0 (28.2) **Disease duration:** 8.8 (7.0) | none | **ActiGraph 7164** accelerometer 1 axis 1 wearable(s) Position: waist | **Physical activity** Association with other measure (ns) Responsiveness to change (ns) |
| **Shammas et al, 2014** [^10.1186/1475-925X-13-10^](https://doi.org/10.1186/1475-925X-13-10) | n=11 (64% female) age: 41 (9.3)  Type: RR: n=8, PP: n=1, SP: n=2 | **Severity:** Group EDSS 1-2.5: EDSS: 1.75 (0.82), Group EDSS 3-5: EDSS: 4.40 (0.89) **Disease duration:** 2.18 (10.67) | none | **Move II activity sensor** accelerometer 3 axes 2 wearable(s) Position: waist, ankle | **Physical activity** Responsiveness to change (s) |
| **Suh et al, 2014** [^10.1007/S12529-013-9382-2^](https://doi.org/10.1007/S12529-013-9382-2) | n=68 (82% female) age: 49.1 (8.8)  Type: RR: n=68 | **Severity:** PDDS: 2 [0-3] **Disease duration:** 12.1 (7.9) | none | **ActiGraph 7164** accelerometer 1 axis 1 wearable(s) Position: waist | **Physical activity** Association with other measure (s) |
| **Blikman et al, 2015** [^10.1016/J.APMR.2014.08.023^](https://doi.org/10.1016/J.APMR.2014.08.023) | n=23 (78% female) age: 45.7 (10.2), range: 24-66  Type: RR: n=20, SP: n=3 | **Severity:** EDSS : 2.0 [3.0] **Disease duration:** 9.3 (7.1), range: 0-21 | **healthy (nonfatigued)** n=23 (78% female) age: 45.7(10.2) | **ActiGraph GT3X** accelerometer 3 axes 1 wearable(s) Position: waist | **Physical activity** Group differences MS vs HC (ss) |
| **Bove et al, 2015** [^10.1212/NXI.0000000000000162^](https://doi.org/10.1212/NXI.0000000000000162) | n=22 (73% female) age: 34.3 (11.0)  Type: RR: n=15, PP: n=9, SP: n=4, NMO: n=1, not reported: n=2 | **Severity:** EDSS: 2.5 (2.4) **Disease duration:** 8.0 (6.0) | **healthy (cohabitants)** n=17 (53% female) age: 39.9 (10.5) | **HTC Sensation 4G (smartphone)** Custom app touchscreen 1 wearable(s) Position: hand | **Dexterity/Tremor** Association with other measure (ss) Group differences MS vs HC (ss) Responsiveness to change (ss) |
| **Fjeldstad et al, 2015** [^10.7224/1537-2073.2014-037^](https://doi.org/10.7224/1537-2073.2014-037) | n=13 (69% female) age: 47.6 (3.0)  Type: RR: n=13 | **Severity:** EDSS: 2.5 (0.5) **Disease duration:** 7.5 (1.0) | **healthy** n=12 (42% female) age: 45.5 (5.4) | **ActiGraph GT1M** accelerometer 2 axes 1 wearable(s) Position: waist | **Physical activity** Association with MS severity (s) Group differences MS vs HC (ns) |
| **Ezeugwu et al, 2015** [^10.1016/J.PMEDR.2015.03.007^](https://doi.org/10.1016/J.PMEDR.2015.03.007) | n=439 (85% female) age: 47.3 (10.0)  Type: RR: n=395, PP: n=9, SP: n=27, benign: n=6, not reported: n=2 | **Severity:** PDDS<=2: n=245, PDDS>=3 : n=194 **Disease duration:** <= 10 years: n=279,  10 years: n=159 | none | **ActiGraph 7164** accelerometer 1 axis 1 wearable(s) Position: waist | **Physical activity** Group differences MS vs MS (ss) |
| **Hubbard et al, 2015** [^10.1139/APNM-2014-0271^](https://doi.org/10.1139/APNM-2014-0271) | n=82 (76% female) age: 49.0 (9.1)  Type: RR: n=65, not reported: n=17 | **Severity:** SR-EDSS: 3.4 (2.3), 3.5 [4.5] **Disease duration:** 11.8 (mean) | none | **ActiGraph GT3X** accelerometer 3 axes 1 wearable(s) Position: waist | **Physical activity** Association with MS severity (ss) Association with other measure (ss) Group differences MS vs MS (ns) |
| **Kahraman et al, 2015** [^10.1016/J.CLINEURO.2015.07.018^](https://doi.org/10.1016/J.CLINEURO.2015.07.018) | n=52 (67% female) age: 36 (8)  Type: RR: n=43, CIS: n=9 | **Severity:** EDSS: 1.5 (1.0) **Disease duration:** 5 (4.0) | none | **Caltrac** accelerometer 3 axes 1 wearable(s) Position: waist | **Physical activity** Group differences MS vs MS (s) |
| **Klaren et al, 2015** [^10.1155/2015/482536^](https://doi.org/10.1155/2015/482536) | n=39 (77% female) age: 48.7 (9.6)  Type: RR: n=30, P: n=9 | **Severity:** EDSS: 4.5 [2.5] **Disease duration:** 10.3 (8.5) | none | **ActiGraph GT3X** accelerometer 3 axes 1 wearable(s) Position: not reported | **Physical activity** Association with other measure (ss) |
| **Motl et al, 2015** [^10.7224/1537-2073.2014-016^](https://doi.org/10.7224/1537-2073.2014-016) | n=41 (73% female) age: 48.4 (9.1)  Type: RR: n=31, P: n=10 | **Severity:** PDDS mild: n=22, PDDS moderate: n=19 **Disease duration:** 10.6 (7.1) | **MS patients (waitlist control)** n=41 (78% female) age: 49.5 (9.2) Type: RR: n=43, PP: n=7, SP: n=7 Severity: PDDS mild: n=17, PDDS moderate: n=24 Disease duration: 13.0 (9.1) | **Yamax SW-401** mechanical pedometer 1 wearable(s) Position: waist | **Physical activity** Group differences MS vs MS (s) Responsiveness to intervention (s) |
| **Rice et al, 2015** [^10.1016/J.APMR.2015.06.011^](https://doi.org/10.1016/J.APMR.2015.06.011) | n=9 (67% female) age: 53.3 (11.1)  Type: RR: n=3, PP: n=2, SP: n=4 | **Severity:** inclusion criteria: full-time wheelchair user **Disease duration:** 13.2 (8.9) | **MS patients** n=5 (80% female) age: 54 (0.4) Type: RR: n=2, PP: n=1, SP: n=2 Severity: inclusion criteria: full-time wheelchair user Disease duration: 17.6 (8.5) | **ActiGraph GT3X** accelerometer 3 axes 1 wearable(s) Position: wrist | **Physical activity** Group differences MS vs MS (ss) Responsiveness to intervention (ns) |
| **Sandroff et al, 2015** [^10.1097/NPT.0000000000000087^](https://doi.org/10.1097/NPT.0000000000000087) | n=31 (87% female) age: 43.4 (7.7)  Type: RR: n=29, SP: n=2 | **Severity:** PDDS: 2 (median), range: 0-5 **Disease duration:** 8.6 (6.3) | **healthy** n=31 (87% female) age: 42.4 (7.5) | **ActiGraph GT3X** accelerometer 3 axes 1 wearable(s) Position: waist | **Physical activity** Association with other measure (s) Group differences MS vs HC (ss) |
| **Sandroff et al, 2015** [^10.1016/J.GAITPOST.2014.10.011^](https://doi.org/10.1016/J.GAITPOST.2014.10.011) | n=96 (80% female) age: 52.7 (11.1)  Type: RR: n=79, P: n=13, not reported: n=4 | **Severity:** EDSS: 4.5 (median), range: 2-6.5 **Disease duration:** 11.8 (10.0) | none | **ActiGraph GT3X** accelerometer 3 axes 1 wearable(s) Position: waist | **Physical activity** Association with other measure (s) Group differences MS vs MS (s) |
| **Sola-Valls et al, 2015** [^10.1007/S00415-015-7764-X^](https://doi.org/10.1007/S00415-015-7764-X) | n=23 (52% female) age: 46.7 (10.0)  Type: RR: n=15, SP: n=8 | **Severity:** EDSS: 3.5 [1.5-6.5] **Disease duration:** 14.2 (9.9) | none | **ActiGraph GT3X** accelerometer 3 axes 1 wearable(s) Position: waist | **Physical activity** Association with MS severity (s) Association with other measure (s) Group differences MS vs MS (s) |
| **Grover et al, 2016** [^10.1016/J.JPEDS.2016.08.104^](https://doi.org/10.1016/J.JPEDS.2016.08.104) | n=27 (67% female) age: 16.0 (4.0)  Type: not reported: n=27 | **Severity:** EDSS: 1.5 [0.5] **Disease duration:** 2.0 (2.0) | **mixed** healthy: n=37 (68% female), age: 15.0 (3.0); monophasic acquired demyelinating syndromes: n=41 (46%), age: 14.0 (4.0), severity: EDSS: 1.0 [2.0], disease duration: 4.0 (7.0) | **ActiGraph 7164** accelerometer 1 axis 1 wearable(s) Position: waist | **Physical activity** Association with other measure (s) Group differences MS vs HC (s) Group differences MS vs other diseases (s) |
| **Kinnett-Hopkins et al, 2016** [^10.1016/J.MSARD.2016.08.010^](https://doi.org/10.1016/J.MSARD.2016.08.010) | n=27 (67% female) age: 15.73 (3.2)  Type: not reported: n=27 | **Severity:** EDSS: 1.5 (0.5) **Disease duration:** 2.03 (2) | **healthy** n=45 (67% female) age: 14.76 (3.8) | **ActiGraph 7164** accelerometer 1 axis 1 wearable(s) Position: waist | **Physical activity** Association with other measure (ss) Group differences MS vs HC (ss) |
| **Klaren et al, 2016** [^10.1123/APAQ.2015-0007^](https://doi.org/10.1123/APAQ.2015-0007) | n=422 (85% female) age: 47.3 (10)  Type: RR: n=380; not reported: n=42 | **Severity:** PDDS: 2.0 [4.0] **Disease duration:** 9.6 (7.4) | none | **ActiGraph 7164** accelerometer 1 axis 1 wearable(s) Position: waist | **Physical activity** Test-retest reliability (s) |
| **Klaren et al, 2016** [^10.14336/AD.2015.1025^](https://doi.org/10.14336/AD.2015.1025) | n=963 (84% female) age: age: Young: 33.1 (4.7), Middle-aged: 49.6 (5.4), Older: 63.0 (3.6)  Type: RR: n=885, not reported: n=78 | **Severity:** EDSS: Young: 1.0 [3.0] Middle-aged: 2.0 [2.0] Older: 3.0 [3.0] **Disease duration:** Young: 6.3 (5.0) Middle-aged: 10.8 (7.2) Older: 16.5 (9.2) | none | **ActiGraph 7164** accelerometer 1 axis 1 wearable(s) Position: waist | **Physical activity** Group differences MS vs MS (s) |
| **Brown et al, 2016** [^10.7224/1537-2073.2015-035^](https://doi.org/10.7224/1537-2073.2015-035) | n=22 (86% female) age: 55.0 (2.5)  Type: RR: n=13, PP: n=4, SP: n=5 | **Severity:** EDSS: 5.1 (0.3) **Disease duration:** 13.3 (1.7) | **MS patients (placebo first and then treatment)** n=21 (52% female) age: 54 (0.4) Type: RR: n=13, PP: n=2, SP: n=6 Severity: EDSS: 5.3 (0.2) Disease duration: 13.5 (1.5) | **StepWatch 3** accelerometer 3 axes 1 wearable(s) Position: ankle | **Physical activity** Group differences MS vs MS (ns) Responsiveness to intervention (ns) |
| **Zoerner et al, 2016** [^10.1177/1352458515622695^](https://doi.org/10.1177/1352458515622695) | n=55 (62% female) age: 48.6 (9.8)  Type: RR: n=29, PP: n=5, SP: n=21 | **Severity:** EDSS: 4.9 (1.3), range: 2.5-6.5 **Disease duration:** 11.9 (7.4) | none | **Actiwatch 2** accelerometer 3 axes 1 wearable(s) Position: ankle | **Physical activity** Responsiveness to intervention (ss) |
| **Aburub et al, 2017** [^10.1016/J.MSARD.2016.12.010^](https://doi.org/10.1016/J.MSARD.2016.12.010) | n=60 age: 35.62 (9.14)  Type: RR: n=60 | **Severity:** EDSS: 2.47 (1.63), range: 0-5 **Disease duration:** 7.29 (4.37) | none | **ActiGraph Actisleep** accelerometer 3 axes 1 wearable(s) Position: wrist  **ActiGraph 7164** accelerometer 1 axis 1 wearable(s) Position: waist | **Physical activity** Association with MS severity (s) Association with other measure (s) |
| **Dalla-Costa et al, 2017** [^10.1016/J.JNS.2017.10.043^](https://doi.org/10.1016/J.JNS.2017.10.043) | n=73 (51% female) age: 47.6 (7.9)  Type: RR: n=53, P: n=20 | **Disease duration:** 14.4 (7.3) | none | **Garmin Forerunner 230** others (GPS) 3 axes 1 wearable(s) Position: wrist | **Physical activity** Association with other measure (ns) |
| **Klaren et al, 2017** [^10.2217/NMT-2016-0036^](https://doi.org/10.2217/NMT-2016-0036) | n=36 (81% female) age: 49.1 (9.1)  Type: RR: n=28, not reported: n=8 | **Severity:** EDSS: 4.5 [2.5] **Disease duration:** 10.1 (8.6) | none | **ActiGraph GT3X+** accelerometer 3 axes 1 wearable(s) Position: waist | **Physical activity** Association with other measure (ns) |
| **Klaren et al, 2017** [^10.1123/JPAH.2016-0335^](https://doi.org/10.1123/JPAH.2016-0335) | n=269 (83% female) age: 45.9 (9.8)  Type: RR: n=269 | **Severity:** PDDS: 2.0 [3.0] **Disease duration:** 8.8 (7.1) | none | **ActiGraph 7164** accelerometer 1 axis 1 wearable(s) Position: waist | **Physical activity** Group differences MS vs MS (ns) Responsiveness to change (ns) |
| **Krueger et al, 2017** [^10.1186/S12883-016-0783-0^](https://doi.org/10.1186/S12883-016-0783-0) | n=26 (69% female) age: 50.9 (5.2)  Type: RR: n=18, PP: n=1, SP: n=7 | **Severity:** EDSS: 4.0 (median), range: 1.5-6.0 | **healthy** n=30 (67% female) age: 49.7 (8.3) | **SenseWear Model mini** accelerometer 2 axes 1 wearable(s) Position: upper arm | **Physical activity** Association with MS severity (ss) Association with other measure (s) Group differences MS vs HC (ss) |
| **Motl et al, 2017** [^10.1016/J.JNS.2016.11.070^](https://doi.org/10.1016/J.JNS.2016.11.070) | n=49 (86% female) age: 50.1 (8.3)  Type: not reported: n=49 | **Severity:** EDSS: 4.75 [2.0], PDDS: 4.0 [2.0] | none | **ActiGraph 7164** accelerometer 1 axis 1 wearable(s) Position: waist | **Physical activity** Association with MS severity (s) Association with other measure (s) |
| **Norris et al, 2017** [^10.1016/J.GAITPOST.2017.02.005^](https://doi.org/10.1016/J.GAITPOST.2017.02.005) | n=26 age: 44.5 (11.9)  Type: RR: n=21, PP: n=2, SP: n=1, benign: n=2, not reported: n=1 | **Severity:** inclusion criteria: PDDS <=3 **Disease duration:** 6.5 (6.2) | none | **SenseWear Armband** accelerometer 2 axes 1 wearable(s) Position: wrist | **Physical activity** Test-retest reliability (ns) |
| **Sebastião et al, 2017** [^10.1097/PHM.0000000000000581^](https://doi.org/10.1097/PHM.0000000000000581) | n=47 age: 53.1 (11.4)  Type: RR: n=44, PP: n=1, SP: n=2 | **Severity:** EDSS: 4 [3.0] **Disease duration:** 13.1 (9.6) | none | **ActiGraph GT3X** accelerometer 3 axes 1 wearable(s) Position: not reported | **Physical activity** Group differences MS vs MS (s) |
| **Sebastião et al, 2017** [^10.3233/NRE-161401^](https://doi.org/10.3233/NRE-161401) | n=21 (90% female) age: 55.5 (9.1)  Type: RR: n=18, not reported: n=3 | **Severity:** EDSS: 6.0 [2.0] **Disease duration:** 14.7 (10.5) | **MS patients (normal fall risk)** n=26 (88% female) age: 51.2 (12.9) Type: RR: n=26 Severity: EDSS: 3.0 [1.5] Disease duration: 13.5 (1.5) | **ActiGraph GT3X** accelerometer 3 axes 1 wearable(s) Position: waist | **Physical activity** Group differences MS vs MS (s) |
| **Bernhard et al, 2018** [^10.1186/S12883-018-1111-7^](https://doi.org/10.1186/S12883-018-1111-7) | n=23 age: NA  Type: not reported: n=23 |  | none | **Rehawatch** accelerometer, gyroscope, magnetometer 3 axes 3 wearable(s) Position: lower back, ankle | **Gait** Group differences MS vs other diseases (ss) |
| **Cederberg, 2018** [^10.1123/JAPA.2016-0358^](https://doi.org/10.1123/JAPA.2016-0358) | n=40 (75% female) age: 60 (5.0)  Type: RR: n=28, PP: n=1, SP: n=3, not reported: n=8 | **Severity:** EDSS: 4.5 [2.5] **Disease duration:** 18 (14) | none | **ActiGraph GT3X+** accelerometer 3 axes 1 wearable(s) Position: waist | **Physical activity** Association with other measure (ss) |
| **Dasmahapatra et al, 2018** [^10.1159/000488040^](https://doi.org/10.1159/000488040) | n=114 (75% female) age: 52 (9)  Type: RR: n=90, PP: n=10, SP: n=14 | **Severity:** MSRS: 32 (16) **Disease duration:** 16 (10) | none | **Fitbit One** accelerometer 3 axes 1 wearable(s) Position: others (belt, pocket or bra) | **Physical activity** Association with other measure (ss) |
| **Engelhard et al, 2018** [^10.1016/J.GAITPOST.2017.10.015^](https://doi.org/10.1016/J.GAITPOST.2017.10.015) | n=88 (83% female) age: MS severity: mild: 41.43 (9.94), moderate: 47.19 (7.85), severe: 46 (8.66)  Type: RR: n=73, PP: n=2, SP: n=8, PR: n=5 | **Severity:** EDSS: mild: range: 0-2.5, moderate: range: 3-4, severe: range: 4.5-6.5 **Disease duration:** MS severity: mild: 13.17 (6.87), moderate: 18.29 (8.3), severe: 18.55 (5.07) | **healthy** n=38 (71% female) age: 35.1 (12.4) | **ActiGraph GT3X** accelerometer 3 axes 1 wearable(s) Position: waist  **Polar S610i** others (ECG) 1 wearable(s) Position: others (chest) | **Physical activity** Association with MS severity (ss) Association with other measure (ss) Group differences MS vs HC (s) Group differences MS vs MS (ss) |
| **Fakolade et al, 2018** [^10.3138/PTC.2017-36.EP^](https://doi.org/10.3138/PTC.2017-36.EP) | n=14 (71% female) age: 52.0 (11.7)  Type: RR: n=6, PP: n=3, SP: n=2, not reported: n=3 | **Disease duration:** 13.2 (8.2) | **healthy (caregivers)** n=14 (29% female) age: 54.1 (13.5) | **Actical** accelerometer 3 axes 1 wearable(s) Position: waist | **Physical activity** Group differences MS vs HC (ns) |
| **Ketelhut et al, 2018** [^10.1080/09638288.2017.1336647^](https://doi.org/10.1080/09638288.2017.1336647) | n=34 (76% female) age: 53.8 (12.4)  Type: RR: n=29, SP: n=2, not reported: n=3 | **Disease duration:** 13.7 (8.6) | none | **ActiGraph GT3X** accelerometer 3 axes 1 wearable(s) Position: waist | **Physical activity** Association with other measure (ss) |
| **Motl et al, 2018** [^10.1037/REP0000162^](https://doi.org/10.1037/REP0000162) | n=684 (81% female) age: 47.1 (10.2)  Type: RR: n=598, P: n=71, not reported: n=15 | **Severity:** PDDS: 2.0 [3.0] **Disease duration:** 9.8 (7.5) | none | **ActiGraph 7164** accelerometer 1 axis 1 wearable(s) Position: waist | **Physical activity** Association with other measure (ss) |
| **Neven et al, 2018** [^10.1177/0361198118772952^](https://doi.org/10.1177/0361198118772952) | n=108 (61% female) age: NA  Type: not reported: n=108 |  | none | **GPS Logger** others (GPS) 3 axes 1 wearable(s) Position: others (pocket or handbag) | **Physical activity** Group differences MS vs MS (ns) |
| **Supratak et al, 2018** [^10.3389/FNEUR.2018.00561^](https://doi.org/10.3389/FNEUR.2018.00561) | n=32 (53% female) age: 39.9 (8.6)  Type: not reported: n=32 | **Severity:** EDSS: 2.5 (mean), range: 1-6 | **healthy** n=22 (50% female) age: 39.8 (8.9) | **Axivity AX3** accelerometer 3 axes 1 wearable(s) Position: lower back | **Physical activity** Association with other measure (ss) |
| **Baird et al, 2019** [^10.1016/J.MSARD.2019.07.003^](https://doi.org/10.1016/J.MSARD.2019.07.003) | n=124 (75% female) age: age: young: 33.3 (5.0), middle-aged 48.5 (5.8), older 65.8 (4.5)  Type: RR: n=107, not reported: n=17 | **Disease duration:** young: 6.7 (5.0), middle-aged: 12.4 (5.8), older: 19.9 (8.7) | none | **ActiGraph GT3X+** accelerometer 3 axes 1 wearable(s) Position: waist | **Physical activity** Group differences MS vs MS (ss) |
| **Block et al, 2019** [^10.1001/JAMANETWORKOPEN.2019.0570^](https://doi.org/10.1001/JAMANETWORKOPEN.2019.0570) | n=95 (62% female) age: 49.6 (13.6)  Type: not reported: n=95 | **Severity:** EDSS: 4.0 (median), range: 0-6.5 | none | **Fitbit Flex** accelerometer 3 axes 1 wearable(s) Position: wrist | **Physical activity** Association with MS severity (s) Group differences MS vs MS (ss) Responsiveness to change (ss) |
| **Block et al, 2019** [^10.1177/2055217319888660^](https://doi.org/10.1177/2055217319888660) | n=31 (55% female) age: 53.4 (11.7)  Type: RR: n=16, P: n=15 | **Severity:** EDSS: 4.0 (mean), range: 0-6.5 **Disease duration:** 17.5 [8.5-23] | none | **ActiGraph GT3X** accelerometer 3 axes 1 wearable(s) Position: waist  **Fitbit Flex** accelerometer 3 axes 1 wearable(s) Position: wrist  **Fitbit Flex2** accelerometer 3 axes 1 wearable(s) Position: wrist | **Physical activity** Association with other measure (ss) |
| **Bollaert et al, 2019** [^10.1519/JPT.0000000000000163^](https://doi.org/10.1519/JPT.0000000000000163) | n=40 (62% female) age: 65.3 (4.3)  Type: RR: n=27, not reported: n=13 | **Severity:** EDSS: 4.0 [2.0] **Disease duration:** 21.5 (8.6) | **healthy** n=40 (63% female) age: 66.5 (6.7) | **ActiGraph GT3X+** accelerometer 3 axes 1 wearable(s) Position: not reported | **Physical activity** Group differences MS vs HC (ss) |
| **Braakhuis et al, 2019** [^10.1186/S12984-019-0573-1^](https://doi.org/10.1186/S12984-019-0573-1) | n=212 (74% female) age: 47.9 (10.4)  Type: RR: n=155, PP: n=22, SP: n=21, not reported: n=14 | **Severity:** EDSS: 2.5 [1.5] **Disease duration:** 6.4 (7.5) | none | **ActiGraph GT3X+** accelerometer 3 axes 1 wearable(s) Position: waist | **Physical activity** Group differences MS vs MS (s) |
| **Cederberg et al, 2019** [^10.1016/J.JNS.2019.116531^](https://doi.org/10.1016/J.JNS.2019.116531) | n=253 (81% female) age: 59.4 (10)  Type: RR: n=170, PP: n=29, SP: n=51, benign: n=3 | **Disease duration:** 20.3 (9.7) | none | **ActiGraph GT3X+** accelerometer 3 axes 1 wearable(s) Position: waist | **Physical activity** Association with other measure (ss) |
| **Kratz et al, 2019** [^10.1093/ABM/KAY018^](https://doi.org/10.1093/ABM/KAY018) | n=107 (69% female) age: 45.16 (11.73)  Type: RR: n=78, P: n=29 | **Disease duration:** 9.49 (8.36) | none | **PRO-Diary** accelerometer 3 axes 1 wearable(s) Position: wrist | **Physical activity** Association with other measure (ss) |
| **Midaglia et al, 2019** [^10.2196/14863^](https://doi.org/10.2196/14863) | n=76 (70% female) age: 39.5 (7.9)  Type: RR: n=69, PP: n=3, SP: n=4 | **Severity:** EDSS: 2.4 (1.4) **Disease duration:** 11.3 (7.0) | **healthy** n=25 (28% female) age: 34.9 (9.3) | **Samsung Galaxy S7 (smartphone)** Floodlight app accelerometer, gyroscope, touchscreen 3 axes 1 wearable(s) Position: hand, upper leg or waist  **Motorola 360 Sport** accelerometer, gyroscope 3 axes 1 wearable(s) Position: wrist | **Physical activity, Gait, Balance, Dexterity/Tremor** Subjective participant acceptability (ss) |
| **Motl et al, 2019** [^10.1016/J.DHJO.2019.05.002^](https://doi.org/10.1016/J.DHJO.2019.05.002) | n=275 (81% female) age: 57 (10.1)  Type: RR: n=181, not reported: n=94 | **Disease duration:** 20.4 (9.7) | none | **ActiGraph GT3X+** accelerometer 3 axes 1 wearable(s) Position: waist | **Physical activity** Association with other measure (ss) |
| **Motl et al, 2019** [^10.1037/REP0000280^](https://doi.org/10.1037/REP0000280) | n=295 (76% female) age: 59.7 (10.1)  Type: RR: n=181, not reported: n=114 | **Disease duration:** 20.4 (9.7) | none | **ActiGraph GT3X+** accelerometer 3 axes 1 wearable(s) Position: waist | **Physical activity** Association with other measure (ss) |
| **Bollaert et al, 2019** [^10.7224/1537-2073.2018-001^](https://doi.org/10.7224/1537-2073.2018-001) | n=40 (62% female) age: 65.3 (4.3)  Type: RR: n=27, not reported: n=13 | **Severity:** EDSS: 4.0 [2.0] **Disease duration:** 21.5 (8.6) | none | **ActiGraph GT3X+** accelerometer 3 axes 1 wearable(s) Position: waist | **Physical activity** Association with other measure (s) |
| **Rooney et al, 2019** [^10.1080/09638288.2019.1634768^](https://doi.org/10.1080/09638288.2019.1634768) | n=91 (68% female) age: 48.1 (11.9)  Type: RR: n=63, PP: n=14, SP: n=14 | **Severity:** EDSS: 2.9 (1.5), range: 0-6.5 **Disease duration:** 8.8 (7.2) | none | **Axivity AX3** accelerometer 3 axes 1 wearable(s) Position: upper leg | **Physical activity** Association with other measure (s) |
| **Sasaki et al, 2019** [^10.1080/02640414.2018.1554614^](https://doi.org/10.1080/02640414.2018.1554614) | n=63 (76% female) age: 56.6 (7.3)  Type: RR: n=50, SP: n=7, P: n=3, not reported: n=3 |  | none | **ActiGraph GT3X** accelerometer 3 axes 1 wearable(s) Position: waist | **Physical activity** Association with other measure (s) |
| **Silveira et al, 2019** [^10.1016/J.CONCTC.2019.100366^](https://doi.org/10.1016/J.CONCTC.2019.100366) | n=22 (91% female) age: 52.2 (10.5)  Type: RR: n=19, not reported: n=3 | **Severity:** PDDS: 1.5 [4.0], EDSS: 3.5 [2.0] **Disease duration:** 13.9 (10.3) | none | **ActiGraph GT3X+** accelerometer 3 axes 1 wearable(s) Position: waist | **Physical activity** Responsiveness to change (ss) |
| **Bourke et al, 2020** [^10.3390/S20205906^](https://doi.org/10.3390/S20205906) | n=76 (70% female) age: 39.5 (7.9)  Type: RR: n=69, PP: n=3, SP: n=4 | **Severity:** EDSS: 2.4 (1.4) **Disease duration:** 11.3 (7.0) | **healthy** n=25 (28% female) age: 34.9 (9.3) | **Samsung Galaxy S7 (smartphone)** Floodlight app accelerometer, gyroscope 3 axes 1 wearable(s) Position: upper leg or waist | **Gait** Test-retest reliability (ss) Group differences MS vs HC (ns) |
| **Creagh et al, 2020** [^10.1088/1361-6579/AB8771^](https://doi.org/10.1088/1361-6579/AB8771) | n=71 (69% female) age: 40 (8)  Type: RR: n=64, PP: n=3, SP: n=4 | **Severity:** EDSS: normal 9PHT: 2.1 (1.26), abnormal 9HPT: 3.3 (1.4) | **healthy** n=22 (32% female) age: not reported | **Samsung Galaxy S7 (smartphone)** Floodlight app touchscreen 1 wearable(s) Position: hand | **Dexterity/Tremor** Association with other measure (ss) Group differences MS vs HC (ns) Group differences MS vs MS (ns) |
| **Hibner et al, 2020** [^10.1016/J.MSARD.2020.101941^](https://doi.org/10.1016/J.MSARD.2020.101941) | n=50 (76% female) age: 46 (12)  Type: RR: n=50 | **Severity:** EDSS: 3.3 [2.5-4] | none | **ActiGraph 7164** accelerometer 1 axis 1 wearable(s) Position: waist | **Physical activity** Association with other measure (s) |
| **Mate et al, 2020** [^10.7224/1537-2073.2019-047^](https://doi.org/10.7224/1537-2073.2019-047) | n=125 (78% female) age: 45.4 (10.0)  Type: not reported: n=125 | **Disease duration:** 6.1 (3.4) | none | **activPAL3** accelerometer 3 axes 1 wearable(s) Position: upper leg | **Physical activity** Association with other measure (ns) |
| **Lam et al, 2020** [^10.1177/1352458520968797^](https://doi.org/10.1177/1352458520968797) | n=85 (75% female) age: 46.4 (10.1)  Type: RR: n=51, PP: n=9, SP: n=25 | **Severity:** EDSS: 3.5 [2.5-4.0] **Disease duration:** 11.3 [ 5.1-17.7] | **healthy** n=18 (56% female) age: 45.2 (13.5) | **Bring-your-own-smartphone** Neurokeys app touchscreen 1 wearable(s) Position: hand | **Dexterity/Tremor** Association with MS severity (ss) Association with other measure (ss) Test-retest reliability (ns) Group differences MS vs HC (ss) Group differences MS vs MS (ns) |
| **Mosquera-lopez et al, 2020** [^10.1109/JBHI.2020.3041035^](https://doi.org/10.1109/JBHI.2020.3041035) | n=25 (68% female) age: 54 (mean), range: 33-76  Type: RR: n=11, PP: n=6, SP: n=8 | **Severity:** EDSS: range: 4.0-6.0 | none | **MotioWear** accelerometer 3 axes 1 wearable(s) Position: waist | **Gait, Balance** (other type of result) |
| **Naess-Schmidt et al, 2020** [^10.1080/2331205X.2020.1713280^](https://doi.org/10.1080/2331205X.2020.1713280) | n=28 (79% female) age: 56.3 (8.8)  Type: not reported: n=28 | **Disease duration:** 12.4 (7.4) | **mixed** stroke: n=22 (55%, female), age: 65.1 (10.8); PD: n=34 (29% female), age: 67.9 (7.8); RA: n=15 (100% female), age: 57.3 (17.0) | **Axivity AX3** accelerometer 3 axes 1 wearable(s) Position: upper leg | **Physical activity** Association with other measure (s) Group differences MS vs other diseases (ns) |
| **Nasseri et al, 2020** [^10.7717/PEERJ.9303^](https://doi.org/10.7717/PEERJ.9303) | n=18 (50% female) age: 49.6 (8.5)  Type: P: n=18 | **Severity:** EDSS: 3.5 (median), range: 2.5-6.0 **Disease duration:** 13.1 (5.6) | **MS patients** n=20 (64% female) age: 52.5 (7.3) Type: P: n=20 Severity: EDSS: 3.5 [3.0-6.0] Disease duration: 20.1 (13.0) | **ActiGraph GT3X+** accelerometer 3 axes 1 wearable(s) Position: not reported | **Physical activity** Group differences MS vs MS (ns) Responsiveness to change (s) Responsiveness to intervention (ns) |
| **Neal et al, 2020** [^10.1177/1545968320916159^](https://doi.org/10.1177/1545968320916159) | n=174 (81% female) age: 58.7 (10.4)  Type: RR: n=115, P: n=59 | **Severity:** PDDS: 4.0 [2.0] **Disease duration:** 20.1 (10.2) | **MS patients (non-fatigued)** n=78 (96% female) age: 60.6 (8.9) Type: RR: n=57, P: n=21 Severity: PDDS: 1.0 [0.0-4.0] Disease duration: 20.7 (8.5) | **ActiGraph GT3X+** accelerometer 3 axes 1 wearable(s) Position: waist | **Physical activity** Association with other measure (s) Group differences MS vs MS (s) |
| **Pau et al, 2020** [^10.3390/IJERPH17238848^](https://doi.org/10.3390/IJERPH17238848) | n=45 (51% female) age: sex: women: 49.4 (9.0), men: 51.2 (11.8)  Type: RR: n=30, PP: n=8, SP: n=7 | **Severity:** EDSS: 3.6 (1.8) **Disease duration:** Sex: women: 17.6 (10.2), men: 18.4 (13.4) | **healthy** n=41 (51% female) age: women: 46.7 (14.6), men: 49.6 (14.4) | **ActiGraph GT3X** accelerometer 3 axes 1 wearable(s) Position: wrist | **Physical activity** Group differences MS vs HC (ss) Group differences MS vs MS (ss) |
| **Pratap et al, 2020** [^10.2196/22108^](https://doi.org/10.2196/22108) | n=495 (47% female) age: MS diagnosis: self-referred: 45.2 (11.6), clinic-referred: 48.93 (11.20)  Type: RR: n=423, PP: n=40, SP: n=30, not reported: n=2 | **Severity:** PDDS: normal: n=153, mild disability: n=109, moderate disability: n=142, gait disability: n=2 **Disease duration:** MS diagnosis: self-referred: 11.14 (8.84), clinic-referred: 14.29 (8.89) | **healthy** n=134 (64% female) age: 39.3 (11.4) | **Bring-your-own-smartphone** elevateMS app accelerometer, gyroscope, touchscreen 3 axes 1 wearable(s) Position: hand | **Gait, Balance, Dexterity/Tremor** Association with MS severity (s) Association with other measure (s) Group differences MS vs HC (ns) Group differences MS vs MS (ns) |
| **Sandroff et al, 2020** [^10.1017/S1355617720000284^](https://doi.org/10.1017/S1355617720000284) | n=385 (78% female) age: 49.9 (11.6)  Type: RR: n=299, P: n=45, not reported: n=41 | **Severity:** PDDS: 2.0 [4.0] **Disease duration:** 12.3 (9.4) | none | **ActiGraph GT3X+** accelerometer 3 axes 1 wearable(s) Position: waist | **Physical activity** Association with other measure (s) |
| **Schwab et al, 2020** [^10.1109/JBHI.2020.3021143^](https://doi.org/10.1109/JBHI.2020.3021143) | n=774 age: NA  Type: not reported: n=774 |  | **healthy** n=372 (female ratio not reported) age: not reported | **Bring-your-own-smartphone** Floodlight Open app accelerometer, gyroscope, touchscreen 3 axes 1 wearable(s) Position: pocket, hand | **Gait, Dexterity/Tremor** Group differences MS vs HC (ns) |
| **Stuart et al, 2020** [^10.1177/2055217320975185^](https://doi.org/10.1177/2055217320975185) | n=56 (54% female) age: 53.6 (8.0)  Type: PP: n=32, SP: n=24 | **Severity:** EDSS: 5.7 (1.3) **Disease duration:** 12.2 (8.6) | none | **SenseWear Armband** accelerometer, others (thermometer, skin impedance) 3 axes 1 wearable(s) Position: upper arm | **Physical activity** Association with MS severity (s) Association with other measure (ss) Responsiveness to change (s) |
| **Twose et al, 2020** [^10.1063/5.0022031^](https://doi.org/10.1063/5.0022031) | n=34 age: range: 18-65 [inclusion criteria]  Type: not reported: n=34 | **Severity:** inclusion criteria: EDSS<7.5 | **healthy** n=24 (female ratio not reported) age: not reported | **Bring-your-own-smartphone** Neurokeys app touchscreen 1 wearable(s) Position: hand | **Dexterity/Tremor** Association with MS severity (ns) Association with other measure (ns) Responsiveness to change (ns) |
| **Shah et al, 2020** [^10.1007/S00415-020-09696-5^](https://doi.org/10.1007/S00415-020-09696-5) | n=13 age: 48.69 (11.10)  Type: not reported: n=13 | **Severity:** PREDSS: range: 3.5=6.0 | **healthy** n=21 (female not reported) age: 46.4 (11.1) | **APDM Opal IMU** accelerometer, gyroscope, magnetometer 3 axes 3 wearable(s) Position: lower back, foot | **Gait** Association with MS severity (s) Association with other measure (s) Group differences MS vs HC (ss) |
| **Zhai et al, 2020** [^10.3389/FNEUR.2020.00688^](https://doi.org/10.3389/FNEUR.2020.00688) | n=67 (63% female) age: 42.9 (10.9)  Type: RR: n=34, P: n=33 | **Severity:** EDSS: 3.0 (median), range: 1.0-6.0 **Disease duration:** 8.5 (8.1) | **healthy** n=70 (67% female) age: 41.5 (12.8) | **Samsung Galaxy S4 (smartphone)** Custom app accelerometer, gyroscope, magnetometer 3 axes 1 wearable(s) Position: habitual phone position  **ActiGraph GT3X+** accelerometer 3 axes 1 wearable(s) Position: wrist | **Physical activity** Association with MS severity (ss) Association with other measure (ss) Group differences MS vs HC (s) Group differences MS vs MS (ns) |
| **Abbadessa et al, 2021** [^10.3390/JCM10061160^](https://doi.org/10.3390/JCM10061160) | n=25 (48% female) age: 40.08 (8.87)  Type: RR: n=25 | **Severity:** EDSS: 4.45 (1.39) **Disease duration:** 9.3 (6.6) | none | **Samsung Gear S2 (smartwatch)** accelerometer 3 axes 1 wearable(s) Position: wrist | **Physical activity** Association with MS severity (ss) Association with other measure (ss) |
| **Abonie et al, 2021** [^10.3390/IJERPH18010017^](https://doi.org/10.3390/IJERPH18010017) | n=11 (27% female) age: 57.9 (8.0)  Type: RR: n=6, PP: n=1, SP: n=4 | **Severity:** PDDS: 2.0 [2.0] **Disease duration:** 12 [24] | **MS patients (no tailored information group)** n=10 (30% female) age: 60.9 (9.5) Type: RR: n=4, PP: n=1, SP: n=5 Severity: PDDS: 3.5 [2.0] Disease duration: 9.5 [19.5] | **ActiGraph GT3X+** accelerometer 3 axes 1 wearable(s) Position: waist | **Physical activity** Group differences MS vs MS (ss) Responsiveness to change (ns) Responsiveness to intervention (s) |
| **Abonie et al, 2021** [^10.1177/02692155211024135^](https://doi.org/10.1177/02692155211024135) | n=21 (29% female) age: 59.33 (8.67)  Type: RR: n=11, PP: n=9, SP: n=1 | **Severity:** PDDS: 3.10 (1.26) **Disease duration:** 14.57 (11.84) | none | **ActiGraph GT3X+** accelerometer 3 axes 1 wearable(s) Position: waist | **Physical activity** Association with other measure (ns) |
| **Anens et al, 2021** [^10.1080/09593985.2021.1996498^](https://doi.org/10.1080/09593985.2021.1996498) | n=30 (70% female) age: 49.2 (14.0)  Type: RR: n=19, SP: n=6, benign: n=4, not reported: n=1 | **Severity:** EDSS: 2.0 (median), IQR: 1.9 | none | **StepsCount** accelerometer 1 axis 1 wearable(s) Position: waist  **Yamax SW200** mechanical pedometer 1 wearable(s) Position: waist  **ActiGraph GT9X** accelerometer 3 axes 1 wearable(s) Position: waist | **Physical activity** Association with other measure (ss) |
| **Barrios et al, 2021** [^10.1145/3478098^](https://doi.org/10.1145/3478098) | n=35 (57% female) age: 36.77 (8.93)  Type: not reported: n=35 | **Severity:** EDSS: 2.31 (1.7) | none | **Bring-your-own-smartphone** Custom app touchscreen 1 wearable(s) Position: hand | **Dexterity/Tremor** Association with other measure (NA) Group differences MS vs MS (NA) |
| **Cederberg et al, 2021** [^10.1016/j.dhjo.2021.101133^](https://doi.org/10.1016/j.dhjo.2021.101133) | n=290 (82% female) age: 52.2 (12.1)  Type: RR: n=262, PP: n=6, SP: n=13, benign: n=5, not reported: n=4 | **Severity:** PDDS: 1 (median), IQR: 2 **Disease duration:** 15.0 (9.2) | none | **ActiGraph GT3X+** accelerometer 3 axes 1 wearable(s) Position: waist | **Physical activity** Association with other measure (ss) |
| **Cheng et al, 2021** [^10.1016/J.GAITPOST.2020.11.025^](https://doi.org/10.1016/J.GAITPOST.2020.11.025) | n=76 (70% female) age: 39.5 (7.9)  Type: RR: n=69, PP: n=3, SP: n=4 | **Severity:** EDSS: 2.4 (1.4) **Disease duration:** 11.3 (7.0) | **healthy** n=25 (28% female) age: 34.9 (9.3) | **Samsung Galaxy S7 (smartphone)** Floodlight app accelerometer, gyroscope 3 axes 1 wearable(s) Position: upper leg or waist | **Gait** Association with MS severity (s) Association with other measure (s) Test-retest reliability (s) Group differences MS vs HC (ns) |
| **Creagh et al, 2021** [^10.1109/JBHI.2020.2998187^](https://doi.org/10.1109/JBHI.2020.2998187) | n=73 (68% female) age: MS severity: moderate: 40.5 (6.9), mild: 39.3 (8.3)  Type: not reported: n=73 | **Severity:** EDSS: moderate: 4.2 (0.7), mild: 1.7 (0.8) | **healthy** n=24 (75% female) age: 35.6 (8.9) | **Samsung Galaxy S7 (smartphone)** Floodlight app accelerometer, gyroscope 3 axes 1 wearable(s) Position: upper leg or waist  **Motorola 360 Sport** accelerometer, gyroscope 3 axes 1 wearable(s) Position: wrist | **Gait** Association with MS severity (ss) Association with other measure (ss) Test-retest reliability (ss) Group differences MS vs HC (s) Group differences MS vs MS (s) |
| **Creagh et al, 2021** [^10.1038/s41598-021-92776-x^](https://doi.org/10.1038/s41598-021-92776-x) | n=73 (68% female) age: MS severity: moderate: 40.5 (6.9), mild: 39.3 (8.3)  Type: RR: n=66, PP: n=3, SP: n=4 | **Severity:** EDSS: moderate: 4.2 (0.7), mild: 1.7 (0.8) | **healthy** n=24 (75% female) age: 35.6 (8.9) | **Samsung Galaxy S7 (smartphone)** Floodlight app accelerometer, gyroscope 3 axes 1 wearable(s) Position: upper leg or waist | **Gait** Group differences MS vs HC (ns) Group differences MS vs MS (ns) |
| **Eldemir et al, 2021** [^10.1080/21641846.2021.1923995^](https://doi.org/10.1080/21641846.2021.1923995) | n=40 (82% female) age: 35.8 (9.9)  Type: RR: n=40 | **Severity:** EDDS: 1 (median), range: 0-2 **Disease duration:** 5.0 (median), IQR: 2.25-9.0 | **healthy** n=30 (86.7% female) age: 36.9 (12.3) | **ActiGraph GT3X+** accelerometer, 3 axes 1 wearable(s) Position: waist | **Physical activity** Association with other measure (ss) Group differences MS vs HC (ss) |
| **Guo et al, 2021** [^10.1145/3494970^](https://doi.org/10.1145/3494970) | n=30 (93% female) age: 45.5 (10.4)  Type: not reported: n=30 | **Disease duration:** 11.0 (8.0) | none | **Activinsights GENEActiv** accelerometer 3 axes 1 wearable(s) Position: wrist | **Physical activity** Association with MS severity (ns) Association with other measure (ns) Subjective participant acceptability (ns) |
| **Hildebrand et al, 2021** [^10.1016/j.msard.2021.103270^](https://doi.org/10.1016/j.msard.2021.103270) | n=25 (68% female) age: 53.8 (13.4)  Type: RR: n=11, PP: n=6, SP: n=8 | **Severity:** EDSS: 6.0 (median), IQR: 5.5 – 6.0 | none | **MotioSens** accelerometer, others (GPS) 3 axes 1 wearable(s) Position: waist | **Gait, Balance** Association with other measure (ns) |
| **Jeng et al, 2021** [^10.1080/09638288.2019.1614683^](https://doi.org/10.1080/09638288.2019.1614683) | n=233 (81% female) age: 59.1 (9.8)  Type: RR: n=171, P: n=62 | **Severity:** PDDS: 3 [1-5] **Disease duration:** 19.8 (9.7) | none | **ActiGraph GT3X+** accelerometer 3 axes 1 wearable(s) Position: waist | **Physical activity** Association with MS severity (s) Group differences MS vs MS (s) |
| **Khalil et al, 2021** [^10.3233/NRE-210188^](https://doi.org/10.3233/NRE-210188) | n=50 (78% female) age: 36.70 (10.04)  Type: RR: n=49, not reported: n=1 | **Severity:** EDSS: 2.62 (1.27) **Disease duration:** 8.64 (6.50) | none | **ActiGraph** accelerometer 3 axes 1 wearable(s) Position: waist | **Physical activity** Association with other measure (ss) |
| **Motl et al, 2021** [^10.1177/20552173211057514^](https://doi.org/10.1177/20552173211057514) | n=31 (81% female) age: 63.0 (5.8)  Type: RR: n=29, not reported: n=2 | **Severity:** EDSS: 4.0 (median), IQR: 1.5 **Disease duration:** 18.3 (6.1) | **healthy** n=29 (83% female) age: 63.9 (5.4) | **ActiGraph GT3X+** accelerometer 3 axes 1 wearable(s) Position: waist | **Physical activity** Association with other measure (s) Group differences MS vs HC (s) |
| **Negaresh et al, 2021** [^10.1111/jon.12869^](https://doi.org/10.1111/jon.12869) | n=52 (67% female) age: 37.3 (9.6)  Type: RR: n=41, P: n=11 | **Severity:** EDSS: 2.1 (1.1) **Disease duration:** 4.2 (3.1) | none | **ActiGraph GT1M** accelerometer 1 axis 1 wearable(s) Position: waist | **Physical activity** Association with other measure (ss) |
| **Pau et al, 2021** [^10.1016/j.msard.2021.103081^](https://doi.org/10.1016/j.msard.2021.103081) | n=28 (71% female) age: 50.6 (9.3)  Type: RR: n=19, PP: n=4, SP: n=5 | **Severity:** EDSS: 4.5 (1.9) | **healthy** n=28 (71% female) age: 48.7 (9.5) | **ActiGraph GT3X** accelerometer 3 axes 2 wearable(s) Position: wrist | **Dexterity/Tremor** Association with MS severity (s) Association with other measure (s) Group differences MS vs HC (ss) |
| **Sagawa et al, 2021** [^10.3390/s21113617^](https://doi.org/10.3390/s21113617) | n=41 (71% female) age: 51.3 (12.7)  Type: RR: n=11, PP: n=13, SP: n=17 | **Severity:** EDSS: 5.1 (1.1) **Disease duration:** 13.9 (10.5) | **healthy** n=16 (44% female) age: 48.0 (7.6) | **ActiGraph wGT3X** accelerometer 3 axes 1 wearable(s) Position: waist | **Physical activity** Association with MS severity (ss) Association with other measure (ss) Group differences MS vs HC (ss) |
| **Shah et al, 2021** [^10.1016/J.GAITPOST.2020.11.024^](https://doi.org/10.1016/J.GAITPOST.2020.11.024) | n=15 age: 49 (10.25)  Type: RR: n=15 | **Severity:** inclusion criteria: EDSS<=6.0 | **healthy** n=16 (female ratio not reported) age: 44.7 (10.7) | **APDM instrumented socks prototype** accelerometer, gyroscope 3 axes 2 wearable(s) Position: foot  **APDM Opal IMU** accelerometer, gyroscope, magnetometer 3 axes 1 wearable(s) Position: lower back | **Gait** Group differences MS vs HC (ns) |
| **Silveira et al, 2021** [^10.1016/j.dhjo.2021.101163^](https://doi.org/10.1016/j.dhjo.2021.101163) | n=205 (75% female) age: 49.4 (13.2)  Type: RR: n=183, P: n=16 | **Severity:** PDDS: 1.0 (median), 3.0 (IQR) **Disease duration:** 12.8 (9.4) | none | **ActiGraph GT3X+** accelerometer 3 axes 1 wearable(s) Position: waist | **Physical activity** Association with other measure (ss) Group differences MS vs MS (ns) |
| **Silveira et al, 2021** [^10.1016/j.dhjo.2020.100966^](https://doi.org/10.1016/j.dhjo.2020.100966) | n=122 (78% female) age: 66.0 (11.3)  Type: RR: n=98, PP: n=17, SP: n=7 | **Severity:** PDDS: 1.0 (median), IQR: 3.0 **Disease duration:** 18.8 (14.9) | **MS patients (non-users of fitness trackers)** n=318 (77.4% female) age: 66.1 (6.1) | **Bring-your-own-smartwatch** accelerometer 3 axes 1 wearable(s) Position: wrist  **Bring-your-own-smartphone** accelerometer 3 axes 1 wearable(s) Position: not reported | **Physical activity** Association with other measure (ns) Group differences MS vs MS (s) |
| **Stephens et al, 2021** [^10.1177/1352458520974360^](https://doi.org/10.1177/1352458520974360) | n=19 (84% female) age: 16.5 (1.1)  Type: not reported: n=19 | **Severity:** EDSS: 1.25 (mean), IQR: 0.5 **Disease duration:** 1.7 (2.0), range: 0.1-7 | **healthy** n=21 (81% female) age: 16.0 (1.4) | **ActiGraph GT3X** accelerometer 3 axes 1 wearable(s) Position: not reported | **Physical activity** Association with MS severity (ss) Association with other measure (ns) Group differences MS vs HC (ns) |
| **Woelfle et al, 2021** [^10.2196/30394^](https://doi.org/10.2196/30394) | n=262 (70% female) age: 50.2 (median), IQR: 42.0-58.0  Type: not reported: n=262 |  | none | **Bring-your-own-smartphone** Floodlight Open app accelerometer, gyroscope, touchscreen 3 axes 1 wearable(s) Position: hand, not reported | **Gait, Balance, Dexterity/Tremor** Test-retest reliability (NA) |
| **Arpan et al, 2022** [^10.3390/s22165940^](https://doi.org/10.3390/s22165940) | n=13 (77% female) age: 49.1 (3.5)  Type: not reported: n=13 | **Severity:** EDSS 4.2 (0.18) **Disease duration:** 16.8 (2.9) | **MS patients (non-fallers)** n=13 (85% female) age: 49.2 (2.4) EDSS: 4.3 (0.23) Disease duration: 13.8 (2) | **APDM Opal IMU** accelerometer, gyroscope, magnetometer 3 axes 3 wearable(s) Position: lower back, foot (instrumented socks) | **Gait, Balance** Association with other measure (ss) Group differences MS vs MS (ss) |
| **Block et al, 2022** [^10.1007/s00415-021-10743-y^](https://doi.org/10.1007/s00415-021-10743-y) | n=94 (62% female) age: Fallers: 51.4 (12.5) Non-fallers: 48.2 (15.0)  Type: not reported: n=94 | **Severity:** EDSS: Fallers: 5.5 (median), range: 0.0–6.5 Non-fallers: 2.5 (median), range: 0.0–6.5 **Disease duration:** Fallers: 14.2 (median), IQR: 8.2–22.8 Non-fallers: 7.7 (median), IQR: 3.1–15.9 | **MS patients (non-fallers)** n=44 (61% female) age: 48.2 (15.0) EDSS: 2.5 (median), range: 0.0–6.5 Disease duration: 7.7 (median), IQR: 3.1–15.9 | **Fitbit Flex (smartwatch)** accelerometer 3 axes 1 wearable(s) Position: wrist | **Physical activity** Association with other measure (ss) Group differences MS vs MS (ss) |
| **Block et al, 2022** [^10.3389/fneur.2022.860008^](https://doi.org/10.3389/fneur.2022.860008) | n=94 (62% female) age: 55.5 (13.7)  Type: RR: n=59, P: n=35 | **Severity:** EDSS: 4.0 (median), range: 0-6.5 **Disease duration:** 16.9 (11.9) | none | **Fitbit Flex (smartwatch)** accelerometer 3 axes 1 wearable(s) Position: wrist | **Physical activity** Association with MS severity (ns) Association with other measure (ns) |
| **Brenton et al, 2022** [^10.1212/WNL.0000000000201098^](https://doi.org/10.1212/WNL.0000000000201098) | n=45 (78% female) age: 16.9 (2.7)  Type: RR: n=45 | **Severity:** EDSS 1.5 (Q1 1.5/Q3 2.0) **Disease duration:** 2.8 (2.6) | **healthy** n=85 (65% female) age: 17.7 (3.3) | **ActiGraph GTX** accelerometer 3 axes 1 wearable(s) Position: waist | **Gait** Association with other measure (ss) Group differences MS vs HC (ss) |
| **Cederberg et al, 2022** [^10.1016/j.apmr.2021.12.022^](https://doi.org/10.1016/j.apmr.2021.12.022) | n=218 (82% female) age: 59.3 (10.1)  Type: RR: n=153, SP: n=38, PP: n=24, not reported: n=3 | **Severity:** PDDS: 3.0 (median), IQR: 4.0 **Disease duration:** 19.9 (9.3) | none | **ActiGraph GT3X+** accelerometer 3 axes 1 wearable(s) Position: waist | **Physical activity** Group differences MS vs MS (ss) |
| **Chikersal et al, 2022** [^10.2196/38495^](https://doi.org/10.2196/38495) | n=56 (86% female) age: 43.5 [IQR 37-52]  Type: not reported: n=56 | **Severity:** PDDS 1 (IQR 0-3) **Disease duration:** 13 (IQR 6.7-17.4) | none | **Bring-your-own-smartphone** AWARE app others (GPS) 1 wearable(s) Position: not reported  **Fitbit Inspire HR (smartwatch)** accelerometer, others (PPG) 3 axes Number of wearable: 1 Position: wrist | **Physical activity** (other type of result) |
| **Creagh et al, 2022** [^10.1109/OJEMB.2022.3221306^](https://doi.org/10.1109/OJEMB.2022.3221306) | n=73 (68% female) age: MS severity: moderate: 40.5 (6.9), mild: 39.3 (8.3)  Type: RR: n=66, PP: n=3, SP: n=4 | **Severity:** EDSS: moderate: 4.2 (0.7), mild: 1.7 (0.8) | **healthy** n=24 (75% female) age: 35.6 (8.9) | **Samsung Galaxy S7 (smartphone)** Floodlight app accelerometer, gyroscope 3 axes 1 wearable(s) Position: upper leg or waist | **Gait** Association with MS severity (s) Responsiveness to change (ss) |
| **Ganzetti et al, 2022** [^10.1007/s00415-022-11494-0^](https://doi.org/10.1007/s00415-022-11494-0) | n=62 (68% female) age: 39.7 (7.5)  Type: RR: n=55, PP: n=3, SP: n=4 | **Severity:** EDSS: 2.5 (1.4) **Disease duration:** 9.5 (6.6) | none | **Samsung Galaxy S7 (smartphone)** Floodlight app accelerometer, gyroscope, touchscreen 3 axes 1 wearable(s) Position: hand, upper leg or waist | **Gait, Balance, Dexterity/Tremor** Association with other measure (ss) |
| **Gervasoni et al, 2022** [^10.1016/j.msard.2022.103941^](https://doi.org/10.1016/j.msard.2022.103941) | n=58 (67% female) age: 39.1 (10.6)  Type: RR: n=58 | **Severity:** EDSS: 1.5 (median), range: 2.5 **Disease duration:** 2.0 (1.8) | **healthy** n=20 (45% female) age: 39.3 (8.9) | **Activinsights GENEActiv** accelerometer 3 axes 1 wearable(s) Position: wrist | **Physical activity** Association with MS severity (ss) Association with other measure (ss) Group differences MS vs HC (ss) Group differences MS vs MS (ss) |
| **Graves et al, 2022** [^10.1002/acn3.51705^](https://doi.org/10.1002/acn3.51705) | n=69 (68% female) age: 39.4 (7.8)  Type: RR: n=62 P: n=7 | **Severity:** EDSS 2.43 (1.36) **Disease duration:** 9.1 (6.5) | **healthy** n=18 (33% female) age: 35 (8.9) | **Samsung Galaxy S7 (smartphone)** Floodlight app touchscreen 1 wearable(s) Position: hand | **Dexterity/Tremor** Association with other measure (ss) Group differences MS vs HC (ss) Group differences MS vs MS (ss) |
| **Huynh et al, 2022** [^10.1016/j.dhjo.2022.101314^](https://doi.org/10.1016/j.dhjo.2022.101314) | n=208 (75% female) age: 50.2 (13.2)  Type: RR: n=184, PP: n=5, SP: n=13, not reported: n=6 | **Severity:** PDDS: 1 (3) **Disease duration:** 13.4 (9.6) | **MS patients (Black)** n=67 (82% female) age: 44.7 (12.1) PDDS: 1 (median), IQR: 3 Disease duration: 10.4 (7.0) | **ActiGraph GT3X+** accelerometer 3 axes 1 wearable(s) Position: waist | **Physical activity** Association with other measure (ss) Group differences MS vs MS (ss) |
| **Hvid et al, 2022** [^10.1007/s00415-022-11134-7^](https://doi.org/10.1007/s00415-022-11134-7) | n=17 (71% female) age: 35.8 (9.2)  Type: RR: n=17 | **Severity:** EDSS: 3.0 (1.2) **Disease duration:** 5.1 (4.1) | none | **Axivity AX3** accelerometer Number of axes: not reported 1 wearable(s) Position: upper leg | **Physical activity** Responsiveness to change (ns) Responsiveness to intervention (ns) |
| **Jeng et al, 2022** [^10.1016/j.msard.2021.103312^](https://doi.org/10.1016/j.msard.2021.103312) | n=210 (75% female) age: 49.6 (13.2)  Type: RR: n=186, P: n=18, not reported: n=6 | **Severity:** PDDS: 1 (median), IQR: 0-3 **Disease duration:** 12.8 (9.5) | **MS patients (fatigued)** n=134 (76% female) age: 50.2 (13.5) Severity: PDDS: 0 (median), IQR: 0-2 Disease duration: 12.8 (8.4) | **ActiGraph GT3X+** accelerometer 3 axes 1 wearable(s) Position: waist | **Physical activity** Association with other measure (ss) Group differences MS vs MS (ss) |
| **Jeng et al, 2022** [^10.3390/ijerph191912466^](https://doi.org/10.3390/ijerph191912466) | n=216 (75% female) age: 49.6 (13.3)  Type: RR: n=190, P: n=19, not reported: n=7 | **Severity:** PDDS: 1 (median), IQR: 3 **Disease duration:** 13.0 (9.6) | **MS patients** young: n=59 (76% female), age: 32.9 (4.7); middle-aged: n=93 (74% female), age: 49.3 (5.6); older: n=64 (77% female), age: 65.5 (4.4) | **ActiGraph GT3X+** accelerometer 3 axes 1 wearable(s) Position: waist | **Physical activity** Association with other measure (ss) Group differences MS vs MS (ss) |
| **Jones et al, 2022** [^10.1016/j.msard.2022.103889^](https://doi.org/10.1016/j.msard.2022.103889) | n=441 (88% female) age: 55.4 (12.6)  Type: RR: n=370, PP: n=39, SP: n=71, benign: n=5 | **Severity:** PDDS: 2.0 (median), IQR: 3 **Disease duration:** 17.1 (10.3) | **MS patients (Elevated Depression)** n=127 (75% female) age: 52.8 (12.7) PDSS: 3.0 (median), IQR: 4 Disease duration: 15.2 (9.4) | **ActiGraph GT3X+** accelerometer 3 axes 1 wearable(s) Position: waist | **Physical activity** Group differences MS vs MS (ss) |
| **Keller et al, 2022** [^10.1177/20556683211067362^](https://doi.org/10.1177/20556683211067362) | n=60 (72% female) age: 47.53 (11.73)  Type: RR: n=60 | **Severity:** EDSS Range 1-6.5 | none | **ActiGraph GTX-9** accelerometer 3 axes 1 wearable(s) Position: non-dominant wrist | **Physical activity** Association with MS severity (ss) Association with other measure (ss) |
| **Kim et al, 2022** [^10.1177/15459683221131787^](https://doi.org/10.1177/15459683221131787) | n=41 (68% female) age: 63.8 (6.4)  Type: RR: n=30, not reported: n=11 | **Severity:** EDSS: 4 (median), range: 1.5-6.5 | **healthy** n=79 (78% female) age: 59.7 (9.4) | **ActiGraph GT3X+** accelerometer 3 axes 1 wearable(s) Position: waist | **Physical activity** Association with other measure (s) Group differences MS vs MS (s) |
| **Kinnett-Hopkins et al, 2022** [^10.1016/j.dhjo.2022.101344^](https://doi.org/10.1016/j.dhjo.2022.101344) | n=208 (75% female) age: White: 52.8 (12.9) Black: 44.7 (12.1)  Type: RR: n=184, not reported: n=24 | **Severity:** PDDS: 1 (3) **Disease duration:** White: 14.8 (10.4) Black: 10.4 (7.0) | **MS patients (Black)** n=67 (82% female) age: 44.7 (12.1) PDDS: 1 (median), IQR: 3 Disease duration: 10.4 (7.0) | **ActiGraph GT3X+** accelerometer 3 axes 1 wearable(s) Position: waist | **Physical activity** Association with other measure (ss) Group differences MS vs MS (ss) |
| **Lam et al, 2022** [^10.2196/37614^](https://doi.org/10.2196/37614) | n=102 (74% female) age: 46.4 (10.4)  Type: RR: n=61, SP: n=30, PP: n=11 | **Severity:** EDSS: 3.5 (median), range: 1.5-7.0 **Disease duration:** 5.7 [3.0-13.1] (median) | none | **Bring-your-own-smartphone** Neurokeys app touchscreen Number of axes: not reported 1 wearable(s) Position: hand | **Dexterity/Tremor** Association with other measure (s) Responsiveness to change (NA) |
| **Lam et al, 2022** [^10.1007/978-981-19-8234-7_20^](https://doi.org/10.1007/978-981-19-8234-7_20) | n=1355 age: NA  Type: not reported: n=1355 |  | **healthy** n=1150 (female ratio not reported) age: not reported | **Bring-your-own-smartphone** Floodlight Open app accelerometer, gyroscope, touchscreen 3 axes 1 wearable(s) Position: hand, not reported | **Physical activity, Dexterity/Tremor, Gait** Association with other measure (ss) |
| **Lam et al, 2022** [^10.1111/ene.15162^](https://doi.org/10.1111/ene.15162) | n=94 (72% female) age: RR: 42.0 (9.8), PP: 52.5 (9.4), SP: 53.3 (6.5)  Type: RR: n=54, PP: n=11, SP: n=29 | **Severity:** EDSS: RR: 3.0 (median), IQR: 2.5-4.0, PP: 4.0 (median), IQR: 3.0-5.5, SP: 4.0 (median), IQR: 3.5-6.0 **Disease duration:** RR: 8.3 (median), IQR: 4.4-14.8, PP: 6.1 (median), IQR: 4.0-11.8, SP: 13.9 (median), IQR: 9.6-27.9 | none | **Bring-your-own-smartphone** Neurokeys app touchscreen 1 wearable(s) Position: hand | **Physical activity, Dexterity/Tremor** Association with MS severity (ns) Association with other measure (ns) Responsiveness to change (ns) |
| **Meyer et al, 2022** [^10.3390/s22186982^](https://doi.org/10.3390/s22186982) | n=22 (73% female) age: 51 (9)  Type: not reported: n=22 | **Severity:** PDDS: 0.88 (1.05) | none | **BioStamp nPoint** accelerometer, others (EMG) 3 axes 3 wearable(s) Position: upper leg, others (chest) | **Gait** Association with other measure (ss) Test-retest reliability (ns) |
| **Montalban et al, 2022** [^10.1177/13524585211028561^](https://doi.org/10.1177/13524585211028561) | n=76 (70% female) age: 39.5 (7.9)  Type: RR: n=69, PP: n=3, SP: n=4 | **Severity:** EDSS: 2.4 (1.4) | **healthy** n=25 (female ratio not reported) age: not reported | **Samsung Galaxy S7 (smartphone)** Floodlight app accelerometer, gyroscope, touchscreen 3 axes 1 wearable(s) Position: hand, upper leg or waist | **Gait, Balance, Dexterity/Tremor** Association with MS severity (ss) Association with other measure (ss) Test-retest reliability (ns) |
| **Motl et al, 2022** [^10.1016/j.msard.2022.103833^](https://doi.org/10.1016/j.msard.2022.103833) | n=60 (80% female) age: 49.5 (10.0)  Type: RR: n=48, P: n=11, not reported: n=1 | **Severity:** EDSS: 3.5 (2.0) **Disease duration:** 13.9 (8.6) | none | **ActiGraph GT3X+** accelerometer 3 axes 1 wearable(s) Position: waist | **Physical activity** Association with other measure (ss) |
| **Salomon et al, 2022** [^10.1016/j.msard.2022.104108^](https://doi.org/10.1016/j.msard.2022.104108) | n=132 (70% female) age: 47.13 (11.24)  Type: RR: n=132 | **Severity:** EDSS: 3 (median), IQR: 2-4 | **healthy** n=90 (48% female) age: 46.1 (11.3) | **Axivity AX3 or AX6** accelerometer 3 axes 1 wearable(s) Position: lower back | **Physical activity** Association with MS severity (ss) Association with other measure (ss) Group differences MS vs HC (ss) |
| **Sandroff et al, 2022** [^10.1177/13524585211048397^](https://doi.org/10.1177/13524585211048397) | n=240 (61% female) age: 52.4 (7.0)  Type: PP: n=64, SP: n=176 | **Severity:** EDSS: 6.0 (1.5–6.5) **Disease duration:** 14.7 (9.3) | none | **ActiGraph GT3X+** accelerometer 3 axes 1 wearable(s) Position: waist | **Physical activity** Association with other measure (ss) |
| **Stephens et al, 2022** [^10.1016/j.msard.2021.103467^](https://doi.org/10.1016/j.msard.2021.103467) | n=15 (87% female) age: 16.6 (1.2)  Type: not reported: n=15 | **Severity:** EDSS: 1.5 (median), IQR: 0.6 | none | **Fitbit Charge 2** accelerometer 3 axes 1 wearable(s) Position: wrist  **ActiGraph GT3X** accelerometer 3 axes 1 wearable(s) Position: not reported | **Physical activity** Responsiveness to intervention (ns) |
| **Sun et al, 2022** [^10.1016/j.cmpb.2022.107204^](https://doi.org/10.1016/j.cmpb.2022.107204) | n=337 (72% female) age: 46.3 (9.7)  Type: RR: n=227, SP: n=110 | **Severity:** EDSS: 3.4 (1.3) **Disease duration:** 13.9 (7.6) | none | **Fitbit Charge 2 / 3 (smartwatch)** accelerometer, others (PPG) 3 axes 1 wearable(s) Position: wrist | **Physical activity** Association with other measure (s) |
| **Tonning et al, 2022** [^10.3389/fspor.2022.1006422^](https://doi.org/10.3389/fspor.2022.1006422) | n=24 (79% female) age: 55.5 (7.8)  Type: not reported: n=24 | **Disease duration:** 10.5 (median), IQR: 6-18 | **mixed** PD: n=24, 38% female, age: 66.5 (7.8), disease duration: 7 (median), IQR: 4-9; RA: n=8, 88% female, age: 57.0 (16.1), disease duration: 11.5 (median), IQR: 8-16.5; Stroke: n=14, 50% female, age: 65.1 (12.7), disease duration: 8 (median), IQR: 2-13 | **Axivity AX3** accelerometer 3 axes 1 wearable(s) Position: upper leg | **Physical activity** Association with other measure (ns) Responsiveness to intervention (ns) |
| **Woelfle et al, 2022** [^10.1007/s00415-022-11306-5^](https://doi.org/10.1007/s00415-022-11306-5) | n=31 (68% female) age: 43.4 (12.0)  Type: RR: n=23, PP: n=4, SP: n=2, CIS: n=2 | **Severity:** EDSS: 3.0 (median), range: 1.0-6.0 | **healthy** n=31 (68% female) age: 42.8 (11.9) | **Bring-your-own-smartphone** dreaMS app accelerometer, gyroscope, magnetometer, touchscreen 3 axes 1 wearable(s) Position: hand, others (trouser pocket or belly bag), not reported  **Fitbit Versa 2** accelerometer 1 wearable(s) Position: wrist | **Physical activity, Gait, Balance, Dexterity/Tremor** Association with MS severity (ss) Association with other measure (ss) Test-retest reliability (ns) Group differences MS vs HC (ss) Subjective participant acceptability (ns) |
