# Supplementary material for: Wearable Sensor Technologies to Assess Motor Functions in People With Multiple Sclerosis: Systematic Scoping Review and Perspective
Source: J Med Internet Res. 2023 Jul 27;25:e44428. doi: 10.2196/44428 (PMC10415952; doi:10.2196/44428)
Supplement: Multimedia Appendix 6 [file jmir_v25i1e44428_app6.docx]

**Multimedia Appendix 6. Detailed information on studies focusing on wearables in a laboratory context. Time is expressed in years. Mean (SD) or median [IQR]. MS: multiple sclerosis, HC: healthy controls, RR: relapsing-remitting, PP: primary progressive, SP: secondary progressive, PDDS: patient determined disease steps, EDSS: expanded disability status scale, ns: non-significant, s: significant, ss: some significant, nt: not tested.**

Compare interactive web app: <https://lbourguignon.shinyapps.io/MS-Review/>

| **First author, year**  DOI | **MS Population of interest**  Sample size (% female)  Age  Type of MS | **Severity**  **Duration of disease** | **Comparator population type**  Sample size (% female)  Age | **Wearables**  Type of sensors  Number of axes Number of wearables  Positions | **Functional Domain**  Types or results reported (significance) |
| --- | --- | --- | --- | --- | --- |
| **Hale et al, 2007** [^10.1682/JRRD.2005.09.0155^](https://doi.org/10.1682/JRRD.2005.09.0155) | n=10 (90% female) age: 49 (9)  Type: RR: n=7, SP: n=3 | **Severity:** EDSS: 3.5 (median), range: 1.5-6.0 **Disease duration:** 16.3 (12.2) | **healthy** n=10 (70% female) age: 40 (6) | **TriTrac RT3** accelerometer 3 axes 1 wearable(s) Position: upper back | **Lab: Physical activity** Test-retest reliability (s) Group differences MS vs HC (ns) |
| **Kayes et al, 2009** [^10.1016/J.APMR.2008.10.012^](https://doi.org/10.1016/J.APMR.2008.10.012) | n=31 (68% female) age: 50 (median), range: 34-80  Type: RR: n=11, SP: n=5, P: n=12, benign: n=3 | **Severity:** used assistive devices: n=14 **Disease duration:** 7 (median), range: 1-40 | none | **Actical** accelerometer 3 axes 1 wearable(s) Position: waist  **Polar heart rate monitor** others (ECG) 1 wearable(s) Position: others (chest) | **Lab: Physical activity** Test-retest reliability (ns) Subjective participant acceptability (ns) |
| **Motl et al, 2009** [^10.1016/J.APMR.2009.03.020^](https://doi.org/10.1016/J.APMR.2009.03.020) | n=24 (83% female) age: 43.5 (12.2)  Type: RR: n=21, PP: n=1, benign: n=2 | **Severity:** PDDS: 1.5 (1.4) **Disease duration:** 9.1 (7.3) | **healthy** n=24 (83% female) age: 40.9 (11.4) | **ActiGraph** accelerometer 1 axis 1 wearable(s) Position: waist | **Lab: Physical activity** Association with other measure (ns) Group differences MS vs HC (ns) |
| **Motl et al, 2010** [^10.1016/J.MEDENGPHY.2010.08.015^](https://doi.org/10.1016/J.MEDENGPHY.2010.08.015) | n=24 (83% female) age: 43.0 (11.7)  Type: RR: n=24 | **Severity:** PDDS: 1 (median), range: 0-4 **Disease duration:** 11.1 (8.5) | none | **ActiGraph 7164** accelerometer 1 axis 2 wearable(s) Position: waist, ankle | **Lab: Physical activity, Lab: Gait** Association with MS severity (ns) Association with other measure (ss) |
| **Alaqtash et al, 2011** [^10.1016/J.ENGAPPAI.2011.04.010^](https://doi.org/10.1016/J.ENGAPPAI.2011.04.010) | n=4 (0% female) age: 43.5 (14.5), range: 29-63  Type: RR: n=4 |  | **healthy** n=10 (0% female) age: 26.2 (5.2) | **ADXL330 iMEMS** accelerometer 3 axes 8 wearable(s) Position: waist, upper leg, lower leg, foot | **Lab: Gait** Group differences MS vs HC (ns) |
| **Weikert et al, 2011** [^10.7224/1537-2073-13.4.170^](https://doi.org/10.7224/1537-2073-13.4.170) | n=24 (83% female) age: 42.0 (11.7)  Type: RR: n=24 | **Severity:** PDDS: 1 (median), range: 0-4, MSWS-12 without gait disability: 10.5 (11.1), MSWS-12 with gait disability: 37.8 (11.7) **Disease duration:** 11.1 (8.5) | none | **ActiGraph 7164** accelerometer 1 axis 1 wearable(s) Position: waist  **Cosmed K4b2** others (VO2) 1 wearable(s) Position: others (head) | **Lab: Physical activity** Association with other measure (ss) Group differences MS vs MS (ss) |
| **Schmidt et al, 2011** [^10.1519/JPT.0B013E31820AA921^](https://doi.org/10.1519/JPT.0B013E31820AA921) | n=9 (100% female) age: 55.9 (5.6)  Type: not reported: n=9 | **Severity:** EDSS: 5.2 (mean), 95% CI: 3.9-6.6 | **PD patients** n=11 (36% female) age: 66.8 (mean) Severity: -Modified Hoehn and Yahr Staging: mean 2.9, 95% CI 2.1-3.7 | **StepWatch Activity Monitor** accelerometer 2 axes 1 wearable(s) Position: ankle | **Lab: Physical activity** Group differences MS vs other diseases (ns) |
| **Coote et al, 2012** [^10.1016/J.APMR.2012.05.010^](https://doi.org/10.1016/J.APMR.2012.05.010) | n=30 (70% female) age: Group MS-A: 50.6 (mean), 95% CI: 46.5-54.8, Group MS-B: 56.1 (mean), 95% CI: 49.0-63.2  Type: RR: n=9, PP: n=1, SP: n=15, benign: n=5 | **Severity:** GNDS total score Group MS-A: 10.11 (5.87), Group MS-B: 11.18 (3.74) **Disease duration:** 2 MS groups: Group MS-A: 10.16 (8.07), Group MS-B: 15.36 (9.11) | **healthy** n=15 (73% female) age: 46.1 (mean) | **SenseWear Armband** accelerometer 2 axes 1 wearable(s) Position: upper arm  **activPal** accelerometer 1 axis 1 wearable(s) Position: upper leg  **Oxycon Mobile** others (VO2) 1 wearable(s) Position: others (head) | **Lab: Physical activity** Association with other measure (s) |
| **Motl et al, 2012** [^10.1016/J.GAITPOST.2011.09.005^](https://doi.org/10.1016/J.GAITPOST.2011.09.005) | n=51 (84% female) age: 53.1 (11.3)  Type: RR: n=45, not reported: n=6 | **Severity:** EDSS: 4.0 (median), range: 2.0-6.5 **Disease duration:** 13.4 (9.4) | none | **Actibelt** accelerometer 3 axes 1 wearable(s) Position: waist | **Lab: Physical activity** Association with other measure (s) |
| **Sandroff et al, 2012** [^10.1682/JRRD.2011.03.0063^](https://doi.org/10.1682/JRRD.2011.03.0063) | n=43 (88% female) age: 47.2 (9.1)  Type: RR: n=39, PP: n=2, SP: n=2 | **Severity:** PDDS: 1 (median), range: 0-5, MSWS-12: 18.6 (16.5) **Disease duration:** 10.8 (7.7) | **healthy** n=43 (88% female) age: 46.5 (10.0) | **ActiGraph 7164** accelerometer 1 axis 1 wearable(s) Position: waist  **ActiGraph GTX3** accelerometer 3 axes 1 wearable(s) Position: waist | **Lab: Physical activity** Association with other measure (ns) Group differences MS vs HC (ss) |
| **Spain et al, 2012** [^10.1016/J.GAITPOST.2011.11.026^](https://doi.org/10.1016/J.GAITPOST.2011.11.026) | n=31 (61% female) age: 39.8 (mean), range: 24-67  Type: RR: n=28, CIS: n=3 | **Severity:** EDSS: 3.0 (median), range: 0-5.0 **Disease duration:** 6.8 (median), range: 0.2-33 | **healthy** n=28 (67% female) age: 37.4 (mean) | **Xsens** accelerometer, gyroscope, magnetometer 3 axes 6 wearable(s) Position: sternum, lower back, wrist, ankle | **Lab: Physical activity** Group differences MS vs HC (s) |
| **Hilfiker et al, 2013** [^10.1186/1756-0500-6-260^](https://doi.org/10.1186/1756-0500-6-260) | n=18 (67% female) age: 54 (11), range: 37-72  Type: not reported: n=18 | **Severity:** EDSS: 5.11 (1.27), range: 3-6.5 | none | **Dynaport** accelerometer 3 axes 1 wearable(s) Position: lower back | **Lab: Physical activity** Responsiveness to intervention (s) |
| **Huisinga et al, 2013** [^10.1007/S10439-012-0697-Y^](https://doi.org/10.1007/S10439-012-0697-Y) | n=15 (73% female) age: 43.75 (11.9)  Type: not reported: n=15 | **Severity:** EDSS: 4.21 [1.0] | **healthy** n=15 (80% female) age: 42.2 (10.3) | **Xsens MTx** accelerometer, gyroscope, magnetometer 3 axes 6 wearable(s) Position: sternum, lower back, wrist, lower leg | **Lab: Physical activity** Association with MS severity (s) Association with other measure (s) Group differences MS vs HC (s) |
| **Morrison et al, 2013** [^10.1016/J.JNS.2012.10.007^](https://doi.org/10.1016/J.JNS.2012.10.007) | n=32 (69% female) age: 59.2 (12.4)  Type: not reported: n=32 |  | **healthy** n=12 (33% female) age: 64.3 (6.8) | **V94-41** accelerometer 1 axis 2 wearable(s) Position: hand | **Lab: Dexterity/Tremor** Group differences MS vs HC (s) |
| **Carpinella et al, 2014** [^10.1186/1743-0003-11-67^](https://doi.org/10.1186/1743-0003-11-67) | n=21 (43% female) age: 47.4 (9.0)  Type: RR: n=10, PP: n=4, SP: n=7 | **Severity:** EDSS: 7 (median), range: 2-8.5 **Disease duration:** 15 (median), range: 1-33 | **healthy** n=12 (42% female) age: 44.3 (9.5) | **Xsens MTx** accelerometer, gyroscope, magnetometer 3 axes 1 wearable(s) Position: lower arm | **Lab: Physical activity** Group differences MS vs HC (s) |
| **Huisinga et al, 2014** [^10.1016/J.APMR.2014.01.004^](https://doi.org/10.1016/J.APMR.2014.01.004) | n=40 (80% female) age: severity group: mild: 41.4 (10.5), moderate: 50.3 (11.8)  Type: not reported: n=40 | **Severity:** mild MS group: SR-EDSS: 3.9 (1.2) moderate MS group: SR-EDSS: 5.0 (1.3) | **healthy** n=20 (85% female) age: 41.8 (10.7) | **Xsens MTx** accelerometer, gyroscope, magnetometer 3 axes 6 wearable(s) Position: sternum, lower back, wrist, lower leg | **Lab: Physical activity, Lab: Balance** Group differences MS vs HC (ss) Group differences MS vs MS (ss) |
| **Sandroff et al, 2014** [^10.1016/J.JNS.2014.02.024^](https://doi.org/10.1016/J.JNS.2014.02.024) | n=54 (83% female) age: 50.9 (9.2)  Type: RR: n=42, PP: n=6, SP: n=5, not reported: n=1 | **Severity:** EDDS: 4.0 (median), range: 1.0-6.5 **Disease duration:** 11.9 (7.5) | none | **ActiGraph GT3X** accelerometer 3 axes 1 wearable(s) Position: waist  **Cosmed K4b2** others (VO2) 1 wearable(s) Position: others (head) | **Lab: Physical activity** Group differences MS vs MS (s) |
| **Ayache et al, 2015** [^10.1016/J.NEUCLI.2015.09.013^](https://doi.org/10.1016/J.NEUCLI.2015.09.013) | n=16 (75% female) age: 49.6 (10.7), range: 31-74  Type: not reported: n=16 | **Severity:** EDSS: 4.4 (2.4), range: 1.5-8.5 | **MS patients (without tremor)** n=10 (60% female) age: 47.9 (6.6) Severity: EDDS: 33.4 (1.6) Disease duration: not reported | **MiR TREM0000** accelerometer 1 axis 1 wearable(s) Position: hand | **Lab: Dexterity/Tremor** Group differences MS vs MS (s) |
| **Ayache et al, 2015** [^10.1016/J.JNS.2015.09.360^](https://doi.org/10.1016/J.JNS.2015.09.360) | n=18 (78% female) age: 47.7 (10.3)  Type: RR: n=10, P: n=8 | **Severity:** EDSS: 4.3 (2.4) **Disease duration:** 14.4 (11.7) | **MS patients (no visible tremor)** n=14 (64% female) age: 54 (0.4) Type: RR: n=8, P: n=6 Severity: EDSS: 3.5 (1.4) Disease duration: 11.4 (8.7) | **MiR TREM0000** accelerometer 1 axis 2 wearable(s) Position: upper arm, hand | **Lab: Dexterity/Tremor** Group differences MS vs MS (ss) |
| **Carpinella et al, 2015** [^10.1088/1741-2560/12/4/046011^](https://doi.org/10.1088/1741-2560/12/4/046011) | n=20 (40% female) age: 46.4 (8.5)  Type: RR: n=9, PP: n=4, SP: n=7 | **Severity:** EDSS: 6.2 (1.6), range: 2-8.5 **Disease duration:** 15 .4 (11.6), range: 1-33 | **healthy (cohabitants)** n=13 (38% female) age: 44.2 (9) | **Xsens MTx** accelerometer, gyroscope, magnetometer 3 axes 1 wearable(s) Position: hand | **Lab: Dexterity/Tremor** Association with other measure (ss) Group differences MS vs HC (ss) Group differences MS vs MS (ss) |
| **Gong et al, 2015** [^10.1109/BSN.2015.7299400^](https://doi.org/10.1109/BSN.2015.7299400) | n=28 (46% female) age: NA  Type: not reported: n=28 |  | **healthy** n=13 (female ratio not reported) age: not reported | **TEMPO Inertial sensors** accelerometer, gyroscope 3 axes 5 wearable(s) Position: lower back, wrist, ankle | **Lab: Physical activity** Group differences MS vs HC (s) |
| **Gong et al, 2015** [^10.4108/EAI.28-9-2015.2261504^](https://doi.org/10.4108/EAI.28-9-2015.2261504) | n=41 age: NA  Type: not reported: n=41 | **Severity:** inclusion criteria: no participants with severe walking disability (EDSS ≥ 4.5) | none | **TEMPO Inertial sensors** accelerometer, gyroscope 3 axes 5 wearable(s) Position: lower back, wrist, ankle | **Lab: Physical activity** Association with MS severity (s) Association with other measure (s) |
| **Moon et al, 2015** [^10.1155/2015/964790^](https://doi.org/10.1155/2015/964790) | n=17 (65% female) age: 62.8 (7.4)  Type: RR: n=10, PP: n=3, SP: n=4 | **Severity:** EDSS: 6.0 [4.75-6.0] **Disease duration:** 19.2 (9.0) | **healthy** n=17 (71% female) age: 62.8 (5.9) | **Xsens MTx** accelerometer, gyroscope, magnetometer 3 axes 2 wearable(s) Position: lower leg | **Lab: Gait** Association with other measure (ss) Group differences MS vs HC (s) |
| **Solomon et al, 2015** [^10.1186/S12984-015-0066-9^](https://doi.org/10.1186/S12984-015-0066-9) | n=20 (80% female) age: 40 (mean), 95% CI: 35-45  Type: not reported: n=20 | **Severity:** EDSS: 2.0 (median), range: 1.0-2.5 **Disease duration:** 4 (mean), 95% CI: 1-7 | **healthy** n=20 (80% female) age: not reported | **APDM Opal IMU** accelerometer, gyroscope, magnetometer 3 axes 6 wearable(s) Position: sternum, lower back, wrist, ankle | **Lab: Physical activity, Lab: Balance** Association with other measure (s) Group differences MS vs HC (s) |
| **Balto et al, 2016** [^10.1177/2055217316634754^](https://doi.org/10.1177/2055217316634754) | n=45 age: 46.7 (10.0), range: 23-62  Type: RR: n=43, not reported: n=2 | **Severity:** EDSS: 3.0 [1.5], range: 1.0-5.0 **Disease duration:** 11.4 (9.3), range: 0.0-35.0 | none | **Yamax SW-200** mechanical pedometer 1 axis 1 wearable(s) Position: waist  **Fitbit One & Jawbone UP Move** accelerometer 3 axes 2 wearable(s) Position: waist  **Fitbit Flex & Jawbone UP2** accelerometer 3 axes 2 wearable(s) Position: wrist  **Apple iPhone 5 (smartphone)** Apple Health app accelerometer 3 axes 1 wearable(s) Position: others (pocket) | **Lab: Physical activity** Association with MS severity (ns) Association with other measure (ns) |
| **Brodie et al, 2016** [^10.1080/10255842.2016.1140747^](https://doi.org/10.1080/10255842.2016.1140747) | n=5 (100% female) age: 56 (8)  Type: not reported: n=5 | **Severity:** EDSS: 4.3 (1.0) | **healthy** n=5 (100% female) age: 56 (8) | **APDM Opal IMU** accelerometer, gyroscope 3 axes 1 wearable(s) Position: lower back | **Lab: Physical activity** Group differences MS vs HC (s) |
| **Gong et al, 2016** [^10.1109/JBHI.2016.2589902^](https://doi.org/10.1109/JBHI.2016.2589902) | n=28 (75% female) age: 40.5 (9.4)  Type: not reported: n=28 | **Severity:** EDSS: 2.0 [0-4.0] **Disease duration:** 6.7 (5.9) | **healthy** n=13 (53% female) age: 39.3 (10.3) | **TEMPO Inertial sensors** accelerometer, gyroscope 3 axes 5 wearable(s) Position: lower back, wrist, ankle | **Lab: Physical activity** Group differences MS vs HC (s) Group differences MS vs MS (s) |
| **Engelhard et al, 2016** [^10.1016/J.GAITPOST.2016.07.184^](https://doi.org/10.1016/J.GAITPOST.2016.07.184) | n=89 (82% female) age: 46 [38-52], range: 19-61  Type: not reported: n=89 | **Severity:** EDSS: 2.5 [2-3.5], range: 0-7 | **healthy** n=29 (69% female) age: 40 (19-54) | **ActiGraph GT3X** accelerometer 3 axes 1 wearable(s) Position: waist | **Lab: Physical activity** Association with MS severity (s) Association with other measure (ss) |
| **Pau et al, 2016** [^10.1016/J.MSARD.2016.10.007^](https://doi.org/10.1016/J.MSARD.2016.10.007) | n=105 (70% female) age: severity: MS class 1 [EDSS=0-1.5]: 39.6 (8.3), MS class 2 [EDSS=2.0-4.0]: 43.6 (9.3), MS class 3 [EDSS=4.5-6.0]: 52.1 (10.2)  Type: not reported: n=105 | **Severity:** EDSS: MS class 1: 1.0 (0.2), MS class 2: 2.6 (0.6), MS class 3: 4.6 (1.1) | **healthy** n=47 (45% female) age: 39.4 (12.7) | **G-Sensor** accelerometer, gyroscope, magnetometer 3 axes 1 wearable(s) Position: lower back | **Lab: Physical activity** Association with MS severity (s) Association with other measure (s) Group differences MS vs HC (s) Group differences MS vs MS (s) |
| **Dandu et al, 2016** [^10.1109/BSN.2016.7516271^](https://doi.org/10.1109/BSN.2016.7516271) | n=86 age: range: 19-61  Type: not reported: n=86 | **Severity:** EDSS: range: 0-7 | **healthy** n=29 (female ratio not reported) age: range: 19-54 | **ActiGraph** accelerometer 3 axes 1 wearable(s) Position: waist | **Lab: Physical activity** Association with MS severity (s) Association with other measure (s) Group differences MS vs HC (s) |
| **Qureshi et al, 2017** [^10.1109/BSN.2017.7936025^](https://doi.org/10.1109/BSN.2017.7936025) | n=65 age: range: 18-64  Type: not reported: n=65 |  | none | **Sensor Nodes** accelerometer 3 axes 2 wearable(s) Position: ankle | **Lab: Gait** Association with MS severity (s) Association with other measure (s) |
| **Coulter et al, 2017** [^10.1016/J.MEDENGPHY.2017.03.008^](https://doi.org/10.1016/J.MEDENGPHY.2017.03.008) | n=20 (55% female) age: 53.7 (7.4)  Type: not reported: n=20 | **Severity:** EDSS: 5.85 (0.75), 6 [0.5], range: 4-6.5 | none | **activPAL 3** accelerometer 3 axes 1 wearable(s) Position: upper leg | **Lab: Physical activity** (other type of result) |
| **Craig et al, 2017** [^10.1186/S12984-017-0251-0^](https://doi.org/10.1186/S12984-017-0251-0) | n=15 (80% female) age: 48.2 (8.7)  Type: RR: n=15 | **Severity:** EDSS: 1.89 (0.98) **Disease duration:** 12.2 (5.9) | **healthy** n=15 (80% female) age: 47.8 (9.5) | **APDM Opal IMU** accelerometer, gyroscope, magnetometer 3 axes 6 wearable(s) Position: sternum, lower back, wrist, ankle | **Lab: Gait** Test-retest reliability (ss) |
| **Craig et al, 2017** [^10.1016/J.CLINBIOMECH.2017.07.011^](https://doi.org/10.1016/J.CLINBIOMECH.2017.07.011) | n=40 age: 40 (9)  Type: RR: n=40 | **Severity:** EDSS: 1.63 (0.7) | **healthy** n=40 (female ratio not reported) age: 44 (10) | **APDM Opal IMU** accelerometer 3 axes 2 wearable(s) Position: sternum, ankle | **Lab: Gait** Group differences MS vs HC (ss) |
| **El-Gohary et al, 2017** [^10.1016/J.APMR.2017.01.030^](https://doi.org/10.1016/J.APMR.2017.01.030) | n=52 (79% female) age: 49.5 (9.8)  Type: RR: n=33, PP: n=6, SP: n=13 | **Severity:** self-reported EDSS: 4.3 (0.9) **Disease duration:** 12.7 (10.6) | **healthy** n=21 (67% female) age: 49.9 (11.9) | **APDM Opal IMU** accelerometer, gyroscope, magnetometer 3 axes 3 wearable(s) Position: lower back, ankle, foot | **Lab: Balance** Group differences MS vs HC (ss) |
| **Lorefice et al, 2017** [^10.1007/S00415-017-8612-Y^](https://doi.org/10.1007/S00415-017-8612-Y) | n=60 (68% female) age: 41.5 (11.6)  Type: PP: n=4, not reported: n=56 | **Severity:** EDSS: 2.3 (1.2) **Disease duration:** 11.6 (7.5) | none | **G-Sensor** accelerometer 3 axes 1 wearable(s) Position: lower back | **Lab: Physical activity** Association with other measure (ss) |
| **McGinnis et al, 2017** [^10.1371/JOURNAL.PONE.0178366^](https://doi.org/10.1371/JOURNAL.PONE.0178366) | n=30 (70% female) age: range: 29-74  Type: not reported: n=30 | **Severity:** EDSS: range: 0-7 | **healthy** n=7 (43% female) age: range: 37-71 | **BioSTampRC Sensors** accelerometer 3 axes 5 wearable(s) Position: lower back, upper leg, lower leg | **Lab: Physical activity** Association with other measure (ss) Group differences MS vs MS (ns) |
| **Pau et al, 2017** [^10.1016/J.GAITPOST.2017.08.023^](https://doi.org/10.1016/J.GAITPOST.2017.08.023) | n=50 (42% female) age: 39.4 (12.8)  Type: not reported: n=50 | **Severity:** EDSS: 1.0 (mean) | **healthy** n=50 (42% female) age: 39.4 (12.8) | **G-Sensor** accelerometer 3 axes 1 wearable(s) Position: upper back | **Lab: Gait** Group differences MS vs HC (ss) |
| **Pau et al, 2017** [^10.1016/J.MSARD.2017.04.002^](https://doi.org/10.1016/J.MSARD.2017.04.002) | n=106 (69% female) age: severity: EDSS 0-1.5: 39.8 (8.2), EDSS 2-3.5 43.5 (9.5), EDSS 4-6.5 48.6 (10.1)  Type: RR: n=99, PP: n=1, SP: n=6 | **Severity:** EDSS 0-1.5: 1.0 (0.2), EDSS 2-3.5: 2.6 (0.6), EDSS 4-6.5: 5.2 (1.1) | **healthy** n=42 (28% female) age: 39.6 (13.5) | **G-Sensor** accelerometer 3 axes 1 wearable(s) Position: lower back | **Lab: Balance** Association with MS severity (ns) Group differences MS vs HC (s) |
| **Teufl et al, 2017** [^10.1177/0308022617726259^](https://doi.org/10.1177/0308022617726259) | n=12 (58% female) age: 51.5 (14.1)  Type: RR: n=7, SP: n=5 | **Disease duration:** 13.9 (9.1) | none | **Axivity AX3** accelerometer 3 axes 1 wearable(s) Position: wrist | **Lab: Dexterity/Tremor** Association with other measure (s) Group differences MS vs MS (ns) Responsiveness to change (ns) |
| **Carpinella et al, 2018** [^10.1109/TNSRE.2018.2881324^](https://doi.org/10.1109/TNSRE.2018.2881324) | n=10 (60% female) age: 51 [35-66]  Type: not reported: n=10 | **Disease duration:** 8.5 [7-17] | **mixed** healthy: n=20 (50%, female), age: 57 (mean), range: 51-7; stroke: n=10 (60% female), age: 59 (mean), range: 47-70; PD: n=20 (40% female), age: 73 (mean), range: 61-77 | **Xsens MTw** accelerometer, gyroscope, magnetometer 3 axes 1 wearable(s) Position: sternum | **Lab: Gait** Association with other measure (ss) Group differences MS vs HC (s) Group differences MS vs other diseases (ss) |
| **Findling et al, 2018** [^10.3389/FNEUR.2018.00686^](https://doi.org/10.3389/FNEUR.2018.00686) | n=33 (73% female) age: MS type: remitting: 43.7 (10.5), relapsing: 42.0 (12.7)  Type: RR: n=33 | **Severity:** EDSS: remitting: 2.45 (1.01), relapsing: 3.11 (0.96) **Disease duration:** MS type: remitting: 10.2 (4.5), relapsing: 9.8 (8.2) | **healthy** n=40 (63% female) age: 39.7 (12.6) | **SwayStar** gyroscope 2 axes 1 wearable(s) Position: lower back | **Lab: Balance** Group differences MS vs HC (s) Group differences MS vs MS (s) |
| **Huisinga et al, 2018** [^10.1016/J.HUMOV.2017.12.009^](https://doi.org/10.1016/J.HUMOV.2017.12.009) | n=36 (89% female) age: 45.6 (11.7)  Type: not reported: n=36 | **Severity:** self-reported EDSS: 4.3 (1.2) | **healthy** n=20 (85% female) age: 41.8 (10.7) | **Xsens MTx** accelerometer, gyroscope, magnetometer 3 axes 6 wearable(s) Position: sternum, lower back, wrist, lower leg | **Lab: Balance** Group differences MS vs HC (s) |
| **Pau et al, 2018** [^10.1016/J.MSARD.2017.11.021^](https://doi.org/10.1016/J.MSARD.2017.11.021) | n=45 (91% female) age: 40.5 (10.5)  Type: not reported: n=45 | **Severity:** EDSS: 2.4 (1.2), range: 1-5.5 | **healthy** n=40 (73% female) age: 41.3 (10.4) | **G-Sensor** accelerometer, gyroscope, magnetometer 3 axes 1 wearable(s) Position: lower back | **Lab: Gait** Association with other measure (s) Group differences MS vs HC (ss) |
| **Psarakis et al, 2018** [^10.1088/1361-6579/AAC0A3^](https://doi.org/10.1088/1361-6579/AAC0A3) | n=12 (75% female) age: 52 (9.1)  Type: not reported: n=12 | **Severity:** EDSS: 4.25 [1.1] | **healthy** n=12 (67% female) age: 55.8 (12.3) | **APDM Opal IMU** accelerometer, gyroscope, magnetometer 3 axes 2 wearable(s) Position: lower back, others (head) | **Lab: Gait** Association with MS severity (ss) Association with other measure (ss) Group differences MS vs HC (s) |
| **Dandu et al, 2018** [^10.1109/JBHI.2017.2773629^](https://doi.org/10.1109/JBHI.2017.2773629) | n=115 age: NA  Type: not reported: n=115 |  | **healthy** n=29 (female ratio not reported) age: not reported | **ActiGraph GT3X** accelerometer 3 axes 1 wearable(s) Position: waist  **TEMPO Inertial sensors** accelerometer, gyroscope 3 axes 6 wearable(s) Position: lower back, wrist, ankle | **Lab: Physical activity** Association with other measure (ss) |
| **Sirhan et al, 2018** [^10.1007/S00702-018-1939-4^](https://doi.org/10.1007/S00702-018-1939-4) | n=30 (50% female) age: 38.8 (5.7)  Type: RR: n=26, P: n=4 | **Severity:** EDSS: 3.0 (median), range: 2.0-5.0 **Disease duration:** 11.8 (6.8) | **healthy** n=15 (53% female) age: 37.4 (6.3) | **Axial** accelerometer 3 axes 3 wearable(s) Position: lower back, foot | **Lab: Gait** Association with other measure (ss) Group differences MS vs HC (ss) |
| **Sun et al, 2018** [^10.1159/000485958^](https://doi.org/10.1159/000485958) | n=39 (74% female) age: MS severity: mild: 55.9 (11.3), severe: 60.1 (8.0)  Type: RR: n=30, PP: n=2, SP: n=7 | **Severity:** EDSS: mild: 3.2 (0.6), severe: 6.2 (0.3) **Disease duration:** MS severity: mild: 17.5 (8.5), severe: 20.1 (10.2) | **healthy** n=15 (67% female) age: 57.9 (12.9) | **BioStamp** accelerometer, gyroscope 3 axes 1 wearable(s) Position: lower back  **Xsens MTx** accelerometer, gyroscope, magnetometer 3 axes 1 wearable(s) Position: lower back | **Lab: Balance** Association with other measure (s) Group differences MS vs HC (s) Group differences MS vs MS (s) |
| **Witchel et al, 2018** [^10.3389/FNEUR.2018.00684^](https://doi.org/10.3389/FNEUR.2018.00684) | n=17 (76% female) age: 53.06 (11.06)  Type: not reported: n=17 | **Severity:** self-reported EDSS: 4.0 (1.8) | **healthy** n=23 (61% female) age: 46.13 (11.12) | **x-IMU** accelerometer, gyroscope, magnetometer 3 axes 1 wearable(s) Position: lower back | **Lab: Physical activity** Group differences MS vs HC (ss) |
| **Anastasi et al, 2019** [^10.1002/PMRJ.12137^](https://doi.org/10.1002/PMRJ.12137) | n=9 (44% female) age: 49.6 (16.4)  Type: not reported: n=9 | **Disease duration:** 15.1 (6.8) | **healthy** n=20 (50% female) age: 58 (14.5) | **Xsens MTw** accelerometer, gyroscope, magnetometer 3 axes 1 wearable(s) Position: sternum | **Lab: Gait** Association with other measure (s) Group differences MS vs HC (s) Group differences MS vs other diseases (s) |
| **Angelini et al, 2019** [^10.3390/S20010079^](https://doi.org/10.3390/S20010079) | n=13 (77% female) age: 51 (median), range: 35-63  Type: RR: n=13 | **Severity:** EDSS: 4.5 (median), range: 2.0-6.5 | **MS patients** n=13 (77% female) age: 57 (mean), range: 34-64 Type: SP: n=13 Severity: EDSS: 4.5 [2.5-6.5] Disease duration: not reported | **Xsens MTw** accelerometer, gyroscope, magnetometer 3 axes 3 wearable(s) Position: lower back, ankle  **APDM Opal IMU** accelerometer, gyroscope, magnetometer 3 axes 3 wearable(s) Position: lower back, ankle | **Lab: Gait** Association with other measure (ss) |
| **Flachenecker et al, 2019** [^10.1016/J.MSARD.2019.101903^](https://doi.org/10.1016/J.MSARD.2019.101903) | n=102 (68% female) age: 43.0 (11.6)  Type: RR: n=68, PP: n=12, SP: n=22 | **Severity:** EDSS: 4.0 (median), range: 1.0-7.0 **Disease duration:** 10.1 (10.5) | **healthy** n=22 (46% female) age: 34.2 (15.5) | **Shimmer IMU** accelerometer, gyroscope, magnetometer 3 axes 2 wearable(s) Position: foot | **Lab: Gait** Association with MS severity (s) Association with other measure (s) Test-retest reliability (s) Group differences MS vs HC (s) Group differences MS vs MS (s) |
| **Grinberg et al, 2019** [^10.1016/J.GAITPOST.2019.02.022^](https://doi.org/10.1016/J.GAITPOST.2019.02.022) | n=25 (68% female) age: 35.2 (8.6)  Type: not reported: n=25 |  | **healthy** n=25 (72% female) age: 34.3 (6.1) | **APDM IMU** accelerometer, gyroscope, magnetometer 3 axes 3 wearable(s) Position: lower back, foot | **Lab: Gait** Group differences MS vs HC (ss) |
| **Shema-Shiratzky et al, 2019** [^10.1007/S00415-019-09500-Z^](https://doi.org/10.1007/S00415-019-09500-Z) | n=58 (71% female) age: MS severity: mild: 49 (11.2), moderate 48.9 (8.0)  Type: RR: n=58 | **Severity:** EDSS: mild: 2.5 [2-3], moderate: 5.25 [4-6] **Disease duration:** MS severity: mild: 13.8 (10.9), moderate: 13.6 (7.5) | none | **APDM Opal IMU** accelerometer, gyroscope, magnetometer 3 axes 3 wearable(s) Position: lower back, ankle | **Lab: Balance** Association with other measure (ss) Group differences MS vs MS (ss) |
| **Ader et al, 2020** [^10.3390/BIOS10090128^](https://doi.org/10.3390/BIOS10090128) | n=37 (62% female) age: 45.1 (9.9)  Type: RR: n=37 | **Severity:** EDSS: 1.0 (median), range: 0-4.5 **Disease duration:** 7.4 (7.7) | none | **Kinesis Gait IMU** accelerometer, gyroscope 3 axes 2 wearable(s) Position: lower leg | **Lab: Gait** Test-retest reliability (ns) |
| **Akhbardeh et al, 2020** [^10.1002/ACN3.50988^](https://doi.org/10.1002/ACN3.50988) | n=117 (75% female) age: 47 (12.4)  Type: not reported: n=117 | **Severity:** EDSS: 2.5 (median), range: 1-7 **Disease duration:** 10 (median), range: 0.4-44 | **healthy** n=30 (50% female) age: 39.7 (10.7) | **MYO-band** accelerometer, gyroscope, others (sEMG) 3 axes 1 wearable(s) Position: lower arm, lower leg | **Lab: Balance, Lab: Dexterity/Tremor** Association with MS severity (s) Association with other measure (s) Test-retest reliability (ns) Group differences MS vs HC (s) |
| **Angelini et al, 2020** [^10.1007/S00415-020-09928-8^](https://doi.org/10.1007/S00415-020-09928-8) | n=57 (67% female) age: 49.8 (8.4)  Type: SP: n=57 | **Severity:** EDSS: 5.5 (median), range: 3.0-6.5 | **healthy** n=24 (67% female) age: 49.8 (8.4) | **APDM Opal IMU** accelerometer, gyroscope, magnetometer 3 axes 3 wearable(s) Position: lower back, lower leg | **Lab: Gait** Association with MS severity (s) Test-retest reliability (ns) Group differences MS vs HC (ss) |
| **Brull et al, 2020** [^10.3390/S20154329^](https://doi.org/10.3390/S20154329) | n=3 age: NA  Type: not reported: n=3 | **Severity:** EDSS: 6.5 (median), range: 4.5-7.5 | none | **Xsens MTi-1** accelerometer, gyroscope, magnetometer 3 axes 1 wearable(s) Position: others (tip of cane or crutch)  **HBM C9C** others (force sensor) 1 wearable(s) Position: others (tip of cane or crutch)  **Bosch BMP280** others (barometer) 1 wearable(s) Position: others (tip of cane or crutch) | **Lab: Gait** (other type of result) |
| **Cofré et al, 2020** [^10.1016/J.GAITPOST.2020.02.006^](https://doi.org/10.1016/J.GAITPOST.2020.02.006) | n=30 (83% female) age: 42.5 (9.2)  Type: RR: n=30 | **Severity:** EDSS: 1.2 (0.9), range: 0-2.5 **Disease duration:** <15 | **healthy** n=15 (60% female) age: 36.8 (7.0) | **Cometa accelerometer** accelerometer 3 axes 1 wearable(s) Position: lower back | **Lab: Gait** Group differences MS vs HC (ss) |
| **Cohen et al, 2020** [^10.1007/S00415-020-10276-W^](https://doi.org/10.1007/S00415-020-10276-W) | n=21 (67% female) age: 44 (mean), range: 25-66  Type: RIS: n=21 |  | **healthy** n=32 (72% female) age: 42.4 (mean), range: 20-58 | **Apple iPhone X (smartphone)** MS Screen Test app touchscreen 1 wearable(s) Position: hand | **Lab: Dexterity/Tremor** Association with other measure (ss) Test-retest reliability (s) Group differences MS vs HC (s) |
| **Craig et al, 2020** [^10.1016/J.CLINBIOMECH.2020.105100^](https://doi.org/10.1016/J.CLINBIOMECH.2020.105100) | n=15 (53% female) age: 48 (9.6)  Type: not reported: n=15 | **Severity:** EDSS: 4.4 (1.1) | **MS patients (non-fallers)** n=25 (80% female) age: 44 (9.9) Severity: EDSS: 3.3 (1.9) | **APDM Opal IMU** accelerometer, gyroscope, magnetometer 3 axes 2 wearable(s) Position: lower back, foot | **Lab: Gait** Association with MS severity (ss) Association with other measure (ss) Group differences MS vs MS (s) |
| **Huang et al, 2020** [^10.3390/S20216160^](https://doi.org/10.3390/S20216160) | n=40 (50% female) age: 50.9 (9.8)  Type: P: n=40 | **Severity:** EDSS: 5.5 (1.1) | **healthy** n=15 (60% female) age: 52.7 (4.4) | **BTS Bioengineering G-Walk** accelerometer 3 axes 1 wearable(s) Position: lower back, waist  **BTS Bioengineering FREEEMG-1000** others (sEMG) 8 wearable(s) Position: upper leg, lower leg | **Lab: Gait** Association with MS severity (ss) Association with other measure (ss) Group differences MS vs HC (s) Group differences MS vs MS (ss) |
| **Ibrahim et al, 2020** [^10.1186/S12984-020-00798-9^](https://doi.org/10.1186/S12984-020-00798-9) | n=49 (65% female) age: 41.6 (10.4)  Type: not reported: n=49 | **Severity:** EDSS: 3.7 (median), range: 1-6.5 | none | **Shimmer IMU** accelerometer, gyroscope, magnetometer 3 axes 2 wearable(s) Position: foot | **Lab: Gait** Association with other measure (s) |
| **Daunoraviciene et al, 2020** [^10.1109/MSM49833.2020.9201642^](https://doi.org/10.1109/MSM49833.2020.9201642) | n=31 (61% female) age: sex: women: 40 (12.3) men: 34.4 (11.3)  Type: not reported: n=31 | **Severity:** EDSS: 3.71 (1.43), range: 2.0-6.0 | **healthy** n=23 (74% female) age: women: 28 (4.0), men: 31.14 (5.7) | **Shimmer IMU** accelerometer, gyroscope, magnetometer 3 axes 6 wearable(s) Position: upper leg, lower leg, foot | **Lab: Dexterity/Tremor** Group differences MS vs HC (ss) Group differences MS vs MS (ss) |
| **Daunoraviciene et al, 2020** [^10.3233/THC-208003^](https://doi.org/10.3233/THC-208003) | n=28 (57% female) age: 38 (11.9)  Type: not reported: n=28 | **Severity:** EDSS: range: 2.0-6.5 | **healthy** n=23 (70% female) age: 29.1 (4.6) | **Shimmer IMU** accelerometer, gyroscope, magnetometer 3 axes 6 wearable(s) Position: upper arm, lower arm, hand, upper leg, lower leg, foot | **Lab: Dexterity/Tremor** Group differences MS vs HC (ss) Group differences MS vs MS (ss) |
| **Maillart et al, 2020** [^10.1111/ENE.14091^](https://doi.org/10.1111/ENE.14091) | n=116 (61% female) age: 46 (10)  Type: RR: n=86, P: n=29, not reported: n=1 | **Severity:** EDSS: 3.6 (1.6) **Disease duration:** 12 (7) | **healthy** n=69 (65% female) age: 39 (11) | **Smartphone** MSCopilot app accelerometer, gyroscope, magnetometer, touchscreen 3 axes 1 wearable(s) Position: hand | **Lab: Gait, Lab: Dexterity/Tremor** Association with other measure (s) Test-retest reliability (s) Group differences MS vs HC (s) Subjective participant acceptability (ns) |
| **Meyer et al, 2020** [^10.1109/JBHI.2020.3025049^](https://doi.org/10.1109/JBHI.2020.3025049) | n=37 (70% female) age: 51 (12)  Type: not reported: n=37 | **Severity:** EDSS: 2.99 (1.47) | none | **Biostamp MC10** accelerometer 3 axes 2 wearable(s) Position: sternum, upper leg  **APDM Opal IMU** accelerometer, gyroscope, magnetometer 3 axes 4 wearable(s) Position: sternum, lower back, lower leg | **Lab: Gait, Lab: Balance** Group differences MS vs MS (s) |
| **Pilloni et al, 2020** [^10.1002/ACN3.51224^](https://doi.org/10.1002/ACN3.51224) | n=9 (67% female) age: 52.1 (12.8)  Type: RR: n=2, SP: n=7 | **Severity:** EDSS: 5.3 (1.1) | **MS patients (sham tDCS)** n=6 (83% female) age: 53.5 (9.8) Type: RR: n=3, SP: n=3 Severity: EDSS: 4.5 (1.7) Disease duration: not reported | **BTS Bioengineering G-Walk** accelerometer, gyroscope, magnetometer 3 axes 1 wearable(s) Position: lower back | **Lab: Gait** Group differences MS vs MS (ss) Responsiveness to intervention (ss) |
| **Sato et al, 2020** [^10.1016/J.MSARD.2020.102031^](https://doi.org/10.1016/J.MSARD.2020.102031) | n=29 (90% female) age: 52.03 (9.94)  Type: RR: n=29 | **Severity:** EDSS: 3.26 (2.19) **Disease duration:** 12.75 (8.73) | **MS patients (PMS)** n=29 (66% female) age: 60.38 (8.3) Type: P: n=29 Severity: EDSS: 6.0 (1.7) Disease duration: 21.85 (12.35) | **APDM Opal IMU** accelerometer, gyroscope, magnetometer 3 axes 2 wearable(s) Position: hand, foot | **Lab: Dexterity/Tremor** Group differences MS vs HC (ss) Group differences MS vs MS (ss) |
| **Shamlmoni et al, 2020** [^10.1007/S00702-020-02190-2^](https://doi.org/10.1007/S00702-020-02190-2) | n=26 (62% female) age: 47.9 (9.1)  Type: RR: n=17, P: n=9 | **Severity:** EDSS: 4.5 (median), range: 3.0-5.5 **Disease duration:** 9.3 (7.6) | none | **APDM Opal IMU** accelerometer, gyroscope, magnetomter 3 axes 3 wearable(s) Position: lower back, foot | **Lab: Gait, Lab: Balance** Responsiveness to intervention (ns) |
| **Tulipani et al, 2020** [^10.1016/J.GAITPOST.2020.06.014^](https://doi.org/10.1016/J.GAITPOST.2020.06.014) | n=21 age: 55.3 (8.6)  Type: not reported: n=21 | **Severity:** EDSS: 3.4 (1.2) | **MS patients (non-fallers)** n=17 (female ratio not reported) age: 4.8 (13.5) Severity: EDSS: 2.3 (1.2) Disease duration: not reported | **Biostamp MC10** accelerometer 3 axes 2 wearable(s) Position: sternum, upper leg | **Lab: Balance** Association with MS severity (s) Association with other measure (s) Group differences MS vs MS (s) |
| **Vienne-Jumeau et al, 2020** [^10.3389/FNEUR.2020.00261^](https://doi.org/10.3389/FNEUR.2020.00261) | n=22 (59% female) age: walking aid: yes: 57 (9), no: 59 (13)  Type: P: n=22 | **Severity:** EDSS: walking aid: 6.0 [6.0-6.5], no aid: 3.5 [3.0-6.0] | **healthy** n=10 (60% female) age: 26 (1) | **Xsens IMU** accelerometer, gyroscope, magnetometer 3 axes 2 wearable(s) Position: foot | **Lab: Gait** Association with MS severity (s) Association with other measure (ns) Group differences MS vs MS (ss) Responsiveness to change (s) |
| **Adam et al, 2021** [^10.23919/AE51540.2021.9542904^](https://doi.org/10.23919/AE51540.2021.9542904) | n=24 (71% female) age: 49.6 (12.5)  Type: not reported: n=24 |  | **healthy** n=28 (67% female) age: 41.8 (18.5) | **TDK MPU-6050** accelerometer, gyroscope 3 axes 1 wearable(s) Position: lower arm | **Lab: Dexterity/Tremor** Group differences MS vs HC (ss) |
| **Allum et al, 2021** [^10.1016/j.jns.2021.117432^](https://doi.org/10.1016/j.jns.2021.117432) | n=16 (69% female) age: 54.3 (7.1)  Type: RR: n=13 P: n=3 | **Severity:** EDSS: 3 (median), range 2.5-4.0 **Disease duration:** 12.7 (9.1) | none | **Balance International Innovations GmbH SwayStar** gyroscope 2 axes 1 wearable(s) Position: waist | **Lab: Balance** Responsiveness to intervention (ss) |
| **Angelini et al, 2021** [^10.1109/TBME.2021.3061998^](https://doi.org/10.1109/TBME.2021.3061998) | n=114 (62% female) age: MS severity: mild: 43.6 (10.5), noderate: 43.6 (10.5), severe: 43.6 (10.5)  Type: RR: n=41, PP: n=3, SP: n=70 | **Severity:** EDSS: mild: <=3.5, moderate: range: 4.0-5.5, severe: >=6.0 | **healthy** n=24 (67% female) age: 49.9 (8.3) | **APDM Opal IMU** accelerometer, gyroscope, magnetometer 3 axes 3 wearable(s) Position: lower back, lower leg | **Lab: Gait** Group differences MS vs HC (ss) Group differences MS vs MS (ss) |
| **Delahaye et al, 2021** [^10.3390/s21093189^](https://doi.org/10.3390/s21093189) | n=12 (92% female) age: 55 (48-61)  Type: not reported: n=12 | **Severity:** EDSS 4 and below: n=4 4.5 n=4 5.0 n=1 6.0 n=3 | none | **GlobalSat DG100** others (GPS) Number of axes: n/a 1 wearable(s) Position: upper arm (antenna on right shoulder) | **Lab: Gait** Association with other measure (ss) |
| **Di Giovanni et al, 2021** [^10.1016/j.msard.2021.103036^](https://doi.org/10.1016/j.msard.2021.103036) | n=60 (65% female) age: 50.60 (12.66)  Type: RR: n=37, PP: n=8, SP: n=15 | **Severity:** EDSS: 5.05 (1.80) **Disease duration:** 14.92 (10.32) | **healthy** n=43 (51% female) age: 50.7 (16.8) | **Yost Lab 3-Space Sensor** accelerometer, gyroscope, magnetometer 3 axes 2 wearable(s) Position: wrist | **Lab: Dexterity/Tremor** Association with MS severity (ss) Group differences MS vs HC (s) Group differences MS vs MS (ss) |
| **Gulde et al, 2021** [^10.1155/2021/5589562^](https://doi.org/10.1155/2021/5589562) | n=76 (66% female) age: 50.8 (10.3)  Type: RR: n=40, PP: n=13, SP: n=20, not reported: n=3 | **Severity:** EDSS: 3.9 (1.7) **Disease duration:** 13.3 (8.7) | none | **Microsoft Lumia 550 (smartphone)** accelerometer 3 axes 1 wearable(s) Position: sternum | **Lab: Gait** Association with other measure (ss) Responsiveness to intervention (ss) |
| **Hsieh et al, 2021** [^10.1016/J.GAITPOST.2020.11.011^](https://doi.org/10.1016/J.GAITPOST.2020.11.011) | n=12 (75% female) age: 62.1 (8.1)  Type: RR: n=7, PP: n=1, SP: n=4 | **Severity:** EDSS: 6 [5.3-6.4] **Disease duration:** 18.6 (10.1) | **MS patients (non-assisted device users)** n=15 (87% female) age: 46.5 (12.2) Type: RR: n=14, not reported: n=1 Severity: EDSS: 2.5 [2.5-3.5] Disease duration: 12.9 (11.6) | **Samsung Galaxy S6 (smartphone)** accelerometer 3 axes 1 wearable(s) Position: sternum  **APDM Opal IMU** accelerometer, gyroscope, magnetometer 3 axes 1 wearable(s) Position: attached to smartphone | **Lab: Balance** Association with other measure (ss) Group differences MS vs MS (s) |
| **Hsieh et al, 2021** [^10.2196/25604^](https://doi.org/10.2196/25604) | n=10 (70% female) age: Iteration 1: 53.2 (13.1) Iteration 2: 54.6 (8.7)  Type: RR: n=8, PP: n=1, SP: n=1 | **Severity:** EDSS: Iteration 1: 3 (median), IQR: 2.5-6 Iteration 2: 2.5 (median), IQR: 2.5-6 **Disease duration:** Iteration 1: 14 (5.9) Iteration 2: 16.2 (9.2) | none | **Samsung Galaxy S6 (smartphone)** Steady-MS app accelerometer 3 axes 1 wearable(s) Position: others (chest) | **Lab: Balance** Subjective participant acceptability (ns) |
| **Krysko et al, 2021** [^10.1002/ACN3.51187^](https://doi.org/10.1002/ACN3.51187) | n=68 (72% female) age: 48.3 (12.1)  Type: RR: n=53, PP: n=9, SP: n=6 | **Severity:** EDSS: 2.5 (median), range: 0.0-7.0 **Disease duration:** 10.5 (median), range: 0.1-44.0 | none | **MYO-band** accelerometer, gyroscope, others (sEMG) 3 axes 1 wearable(s) Position: lower arm, lower leg | **Lab: Dexterity/Tremor** Association with MS severity (s) Association with other measure (s) Group differences MS vs MS (s) Responsiveness to change (ss) |
| **Muller et al, 2021** [^10.1186/s12883-021-02361-y^](https://doi.org/10.1186/s12883-021-02361-y) | n=88 (73% female) age: EDSS 0-1.0: 37.9 (10.4) EDSS 1.5-2: 38.3 (11.2) EDSS 2.5-5: 47.9 (9.7)  Type: RR: n=81, PP: n=1, SP: n=5, CIS: n=1 | **Severity:** EDSS: EDSS 0-1.0: 0.8 (0.4) EDSS 1.5-2: 1.9 (0.2) EDSS 2.5-5: 3.1 (0.6) | **healthy** n=31 (71% female) age: 34.6 (8.8) | **Xsens MTw2** accelerometer, gyroscope, magnetometer 3 axes 1 wearable(s) Position: foot | **Lab: Gait** Association with MS severity (ss) Association with other measure (ss) Group differences MS vs HC (ss) Group differences MS vs MS (ss) |
| **Nagasubramony et al, 2021** [^10.1145/3452853.3452862^](https://doi.org/10.1145/3452853.3452862) | n=12 age: NA  Type: not reported: n=12 | **Severity:** EDSS: range: 1-5.5 | **healthy** n=11 (female ratio not reported) age: not reported | **x-io NGIMU** accelerometer, gyroscope, magnetometer 3 axes 3 wearable(s) Position: upper leg, sternum | **Lab: Balance** Association with other measure (s) Group differences MS vs HC (s) |
| **Prochazka et al, 2021** [^10.1109/TNSRE.2021.3051093^](https://doi.org/10.1109/TNSRE.2021.3051093) | n=16 age: 38.6 (16.1)  Type: not reported: n=16 |  | **healthy** n=19 (female ratio not reported) age: 39.6 (9.7) | **Noitom Perception Neuron** accelerometer 3 axes 31 wearable(s) Position: not reported | **Lab: Gait, Lab: Balance** Group differences MS vs HC (ns) |
| **Swanson et al, 2021** [^10.1016/j.msard.2021.102924^](https://doi.org/10.1016/j.msard.2021.102924) | n=30 (67% female) age: 47.8 (12.08)  Type: RR: n=30 | **Severity:** EDSS: 4.0 (median), range: 0-6.5 **Disease duration:** 12.9 (10.67) | **healthy middle-age adults and older adults (two groups)** n=19 (79% female) and n=21 (48% female) age: 47.1 (10.5) and 70.3 (5.9) | **APDM Opal V2** accelerometer, gyroscope, magnetometer, others (barometer) 3 axes 6 wearable(s) Position: foot, lower back, wrist, sternum, waist | **Lab: Gait, Lab: Balance** Group differences MS vs HC (ss) |
| **Tanoh et al, 2021** [^10.1016/j.msard.2021.103164^](https://doi.org/10.1016/j.msard.2021.103164) | n=116 (61% female) age: 46 (10)  Type: RR: n=86, P: n=30 | **Severity:** EDSS: 3.6 (1.6) **Disease duration:** 12 (7) | **healthy** n=69 (65% female) age: 39 (11) | **Bring-your-own-smartphone** MSCopilot app accelerometer, gyroscope, touchscreen 3 axes 1 wearable(s) Position: not reported | **Lab: Gait, Lab: Dexterity/Tremor** Association with MS severity (ss) Group differences MS vs HC (ns) Group differences MS vs MS (s) |
| **Teufl et al, 2021** [^10.1177/2055668320966955^](https://doi.org/10.1177/2055668320966955) | n=5 (60% female) age: 57.6 (15.3)  Type: SP: n=5 | **Disease duration:** 13.5 (3.1) | **healthy** n=10 (50% female) age: 42.4 (10.9) | **Axivity AX3** accelerometer 3 axes 1 wearable(s) Position: wrist | **Lab: Dexterity/Tremor** Group differences MS vs HC (ns) |
| **Trentzsch et al, 2021** [^10.3390/brainsci11111507^](https://doi.org/10.3390/brainsci11111507) | n=562 (70% female) age: 43.15 (12.31)  Type: RR: n=490, PP: n=55, SP: n=13, not reported: n=4 | **Severity:** EDSS: 2.5 (median), IQR: 1.5–3.5 **Disease duration:** 8.57 (7.51) | none | **APDM Opal IMU** accelerometer, gyroscope, magnetometer 3 axes 6 wearable(s) Position: sternum, lower back, wrist, ankle | **Lab: Gait** Association with MS severity (ss) Association with other measure (ss) |
| **van et al, 2021** [^10.3390/sym13091560^](https://doi.org/10.3390/sym13091560) | n=20 (30% female) age: 45 (14)  Type: not reported: n=20 | **Severity:** EDSS 3.7 (1.6) | **mixed** healthy: n=24 (67% female), age: 50 (8); PD: n=20 (30% female); age: 59 (7) | **APDM Opal IMU** accelerometer, gyroscope, magnetometer 3 axes 3 wearable(s) Position: lower back, both ankles | **Lab: Gait** Group differences MS vs HC (s) Group differences MS vs other diseases (ns) |
| **Weed et al, 2021** [^10.3390/s21175806^](https://doi.org/10.3390/s21175806) | n=12 (67% female) age: 61.4 (8.7)  Type: RR: n=6 P: n=6 | **Severity:** PDDS: (mild 1-2) n=2 (moderate 2-4) n=10 **Disease duration:** (years post diagnosis 22.6 (15.7)) | **healthy** n=11 (73% female) age: 59.9 (8.7) | **APDM Opal IMU** accelerometer, gyroscope, magnetometer 3 axes 4 wearable(s) Position: sternum, lower back, ankle | **Lab: Balance, Lab: Gait** Group differences MS vs HC (ss) |
| **Afzal et al, 2022** [^10.1109/TBME.2022.3166705^](https://doi.org/10.1109/TBME.2022.3166705) | n=10 (80% female) age: 54.3 (12.4)  Type: RR: n=5, PP: n=3, SP: n=2 | **Severity:** EDSS: 6.5 (0.4) **Disease duration:** 15.0 (7.1) | none | **Motion Lab MA300-XVI** others (sEMG) 1 wearable(s) Position: lower leg, upper leg  **Force sensor** others (force sensor) 1 wearable(s) Position: foot | **Lab: Gait** Responsiveness to intervention (ss) |
| **Alexander et al, 2022** [^10.1177/13524585221124043^](https://doi.org/10.1177/13524585221124043) | n=125 (73% female) age: Cohort 1: 53 (median), IQR: 49-58 Cohort 3: 53.5 (median), IQR: 47.8-58.0  Type: P: n=110, not reported: n=15 | **Severity:** EDSS: Cohort 1: 6.0 (median), range: 4.0-6.5 Cohort 3: 6.0 (median), range: 1.0-7.5 | **healthy** n=20 (40% female) age: 31.5 (median), IQR: 30.3-34 | **Apple iPhone 6s (smartphone)** mSteps app accelerometer, others (GPS) 3 axes 1 wearable(s) Position: not reported | **Lab: Gait** Association with other measure (ns) Group differences MS vs HC (ns) |
| **Berg-Hansen et al, 2022** [^10.1007/s00415-022-10998-z^](https://doi.org/10.1007/s00415-022-10998-z) | n=46 (63% female) age: 50.2 (8.3)  Type: RR: n=40, PP: n=6 | **Severity:** EDSS: 4.0 (median), range: 1-6 **Disease duration:** 10.5 (median), range: 0-30 | **healthy** n=20 (80% female) age: 47.7 (12.3) | **GaitUp Physiolog 5** accelerometer, gyroscope 3 axes 2 wearable(s) Position: foot | **Lab: Gait** Association with MS severity (ss) Test-retest reliability (ns) Group differences MS vs HC (s) Group differences MS vs MS (ss) |
| **Bois et al, 2022** [^10.1371/journal.pone.0268475^](https://doi.org/10.1371/journal.pone.0268475) | n=22 (59% female) age: 58 (11)  Type: P: n=22 | **Severity:** EDSS 5.0 Range 3.5-6) | **healthy** n=10 (60% female) age: 26 (1) | **Xsens** accelerometer, gyroscope, magnetometer 3 axes 4 wearable(s) Position: head, lower back, foot | **Lab: Gait** Association with MS severity (ns) Group differences MS vs HC (ns) |
| **Carpinella et al, 2022** [^10.3390/s22239558^](https://doi.org/10.3390/s22239558) | n=81 (65% female) age: 39 (median), 5th-95th percentile: 25-56  Type: RR: n=80, PP: n=1 | **Severity:** EDSS: 1.5 (median), 5th-95th percentile. 0-2.5 **Disease duration:** 2 (median), 5th-95th percentile: 0-5 | **healthy** n=38 (58% female) age: 34 (median), 5th-95th percentile: 24-58 | **Xsens MTw** accelerometer, gyroscope, magnetometer 3 axes 1 wearable(s) Position: sternum | **Lab: Balance** Association with MS severity (ss) Association with other measure (ss) Group differences MS vs HC (ss) Group differences MS vs MS (s) |
| **Carpinella et al, 2022** [^10.3389/fneur.2021.821640^](https://doi.org/10.3389/fneur.2021.821640) | n=59 (53% female) age: 37 (median), 5th-95th percentile: 25-53  Type: RR: n=59 | **Severity:** EDSS: 1.5 (median), 5th-95th percentile: 0-2.5 **Disease duration:** 2 (median), 5th-95th percentile: 0-5 | **healthy** n=40 (70% female) age: 37.5 (median), 5th-95th percentile: 24.5-57 | **Xsens MTw** accelerometer, gyroscope, magnetometer 3 axes 3 wearable(s) Position: lower leg, sternum | **Lab: Gait** Association with MS severity (ss) Association with other measure (ss) Group differences MS vs HC (ss) Group differences MS vs MS (ss) |
| **Drouin et al, 2022** [^10.1002/sim.9625^](https://doi.org/10.1002/sim.9625) | n=27 age: NA  Type: not reported: n=27 | **Severity:** EDSS Range 0-6 | none | **Mbientlab MetaMotion R** accelerometer, gyroscope, magnetometer 3 axes 1 wearable(s) Position: waist (belt clip on the right hip) | **Lab: Gait** (other type of result) |
| **Frechette et al, 2022** [^10.2196/32453^](https://doi.org/10.2196/32453) | n=6 age: NA  Type: not reported: n=6 |  | none | **Samsung Galaxy S6 (smartphone)** Steady-Wheels app accelerometer 3 axes 1 wearable(s) Position: others (chest) | **Lab: Balance** Subjective participant acceptability (ns) |
| **Gervasoni et al, 2022** [^10.3389/fimmu.2022.842269^](https://doi.org/10.3389/fimmu.2022.842269) | n=14 (43% female) age: 48.3 (10.3)  Type: RR: n=14 | **Severity:** EDSS: 4.7 (1.2) **Disease duration:** 13.0 (10.2) | **healthy** n=14 (43% female) age: 48.4 (14.6) | **Tcore** others (thermometer) 1 wearable(s) Position: others (head)  **Faros 180** others (ECG) 1 wearable(s) Position: others (chest) | **Lab: Gait** Group differences MS vs HC (ss) |
| **Hossen et al, 2022** [^10.1016/j.ebiom.2022.104152^](https://doi.org/10.1016/j.ebiom.2022.104152) | n=40 (57% female) age: Train: 52.5, range: 35-84 Test: 55.7, range: 43-79  Type: RR: n=40 |  | **mixed** PD: n=39 (41% female), age: Train 64.5 / Test: 68.2; Essential Tremor: n=41 (44% female), age: Train 63.2 / Test 64.5 | **Unspecified accelerometer** accelerometer Number of axes: not reported 1 wearable(s) Position: not reported | **Lab: Dexterity/Tremor** Association with other measure (ns) Group differences MS vs other diseases (ns) |
| **Huang et al, 2022** [^10.3390/brainsci12020258^](https://doi.org/10.3390/brainsci12020258) | n=40 (50% female) age: 50.9 (9.8)  Type: PP: n=7, SP: n=33 | **Severity:** EDSS (pre treatment): 6.0 (3.5–6.5) EDSS (post treatment): 6.0 (3.5–6.5) **Disease duration:** 18.6 (10.1) | none | **BTS Bioengineering G-Walk** accelerometer, gyroscope 3 axes 2 wearable(s) Position: upper leg | **Lab: Gait** Association with other measure (ss) Responsiveness to change (ss) Responsiveness to intervention (ss) |
| **Ibrahim et al, 2022** [^10.1016/j.msard.2022.103519^](https://doi.org/10.1016/j.msard.2022.103519) | n=65 (69% female) age: 41.9 (11.2)  Type: RR: n=46, PP: n=8, SP: n=11 | **Severity:** EDSS: 3.5 (average), range: 1.0-6.5 | none | **Shimmer 3 sensor** accelerometer, gyroscope 3 axes 2 wearable(s) Position: foot | **Lab: Gait** Association with other measure (ss) |
| **Leblanc et al, 2022** [^10.23736/S0393-3660.20.04442-3^](https://doi.org/10.23736/S0393-3660.20.04442-3) | n=28 (57% female) age: 55.6 (8.0)  Type: not reported: n=28 | **Severity:** EDSS: 4.25 (median), IQR: 4-5.5 | **healthy** n=20 (75% female) age: 33.2 (7.6) | **ActiGraph wGT3X** accelerometer 3 axes 2 wearable(s) Position: waist, wrist | **Lab: Physical activity** Association with MS severity (ns) Test-retest reliability (ns) Group differences MS vs HC (ns) Group differences MS vs MS (ns) |
| **Marotta et al, 2022** [^10.3390/jcm11123505^](https://doi.org/10.3390/jcm11123505) | n=17 (71% female) age: 40.6 (14.4)  Type: RR: n=17 | **Severity:** EDSS: 3.0 (median), IQR: 1 | **MS patients** n=8 (75% female) age: 39.75 (8.39) EDSS: 3 (median), IQR: 0 Disease duration: not reported | **G-Sensor** accelerometer, gyroscope, magnetometer 3 axes 1 wearable(s) Position: lower back | **Lab: Gait, Lab: Balance** Group differences MS vs MS (ss) Responsiveness to intervention (ss) |
| **Sato et al, 2022** [^10.1016/j.clinbiomech.2022.105818^](https://doi.org/10.1016/j.clinbiomech.2022.105818) | n=31 (74% female) age: RR: 52.5 (10.9) SP: 60.7 (6.3) PP: 57.3 (9.0)  Type: RR: n=18 SP: n=9 PP: n=4 | **Severity:** EDSS: RR: 2.2 (1.7) SP: 4.4 (1.7) PP: 4.9 (1.7) | **healthy** n=27 (81% female) age: 55.9 (12.3) | **APDM Opal Version 2** accelerometer, gyroscope 3 axes 6 wearable(s) Position: sternum, lower back, wrist, foot | **Lab: Gait** Group differences MS vs HC (ss) Group differences MS vs MS (ns) |
| **Shah et al, 2022** [^10.3390/s22031077^](https://doi.org/10.3390/s22031077) | n=29 age: NA  Type: not reported: n=29 |  | **healthy (impaired fasting glucose)** n=108 (female ratio not reported) age: 71.2 (5.1) | **APDM Opal IMU** accelerometer, gyroscope, magnetometer 3 axes 6 wearable(s) Position: foot, wrist, sternum, lower back | **Lab: Physical activity** Association with other measure (ss) |
| **Warmerdam et al, 2022** [^10.3390/data7100136^](https://doi.org/10.3390/data7100136) | n=21 (62% female) age: 39 (13)  Type: not reported: n=21 |  | **mixed** healthy (young): n=43 (49% female), age: 29 (8); healthy (over 60): n=24 (50% female), age: 72 (6); PD: n=34 (38% female), age: 65 (11); Stroke: n=23 (26% female), age: 68 (16); Chronic low back pain: n=10 (30% female), age: 64 (15); Other: n=12 (25% female), age: 66 (17) | **Noraxon myoMOTION** accelerometer, gyroscope, magnetometer 3 axes 16 wearable(s) Position: head, sternum, upper arm, lower arm, lower back, upper leg, lower leg, ankle, foot, others (pocket) | **Lab: Gait** (other type of result) |
