# Supplementary material for: Wearable Sensor Technologies to Assess Motor Functions in People With Multiple Sclerosis: Systematic Scoping Review and Perspective
Source: J Med Internet Res. 2023 Jul 27;25:e44428. doi: 10.2196/44428 (PMC10415952; doi:10.2196/44428)
Supplement: Multimedia Appendix 7 [file jmir_v25i1e44428_app7.docx]

**Multimedia Appendix 7. Detailed information on studies focusing on wearables in a mixed context (i.e., combination of laboratory and real-world context). Time is expressed in years. Mean (SD) and median [IQR]. MS: multiple sclerosis, HC: healthy controls, RR: remitting relapsing, PP: primary progressive, SP: secondary progressive, PDDS: patient determined disease steps, EDSS: expanded disability status scale, RW: real-world, ns: non-significant, s: significant, ss: some significant, nt: not tested.**

Compare interactive web app: <https://lbourguignon.shinyapps.io/MS-Review/>

| **First author, year**  DOI | **MS Population of interest**  Sample size (% female)  Age  Type of MS | **Severity**  **Duration of disease** | **Comparator population type**  Sample size (% female)  Age | **Wearables**  Type of sensors  Number of axes Number of wearables  Positions | **Functional Domain**  Types or results reported (significance) |
| --- | --- | --- | --- | --- | --- |
| **Pilutti et al, 2012** [^10.1155/2012/868256^](https://doi.org/10.1155/2012/868256) | n=168 (25% female) age: 48.5 (11.6)  Type: RR: n=134, PP: n=11, SP: n=19, not reported: n=4 | **Severity:** PDDS: 3.0 [3.0] **Disease duration:** 13.8 (9.6) | **MS patients** Overweight: n=61 (77% female), age: 51.5 (9.5), Type: RR: n=45, SP: 7, PP: 4, not reported: n=2, severity: PDDS: 3.0 [3.0]; Disease duration: 10.9 (7.8); Obese: n=55 (43% female), age: 52.5 (10.6), Type: RR: n=46, SP: 4, PP: 4, not reported: n=1, severity: PDDS: 3.0 [3.0]; Disease duration: 11.2 (10.3) | **ActiGraph GT3X** accelerometer 3 axes 1 wearable(s) Position: waist | **RW: Physical activity, Lab: Physical activity** Group differences MS vs MS (ns) |
| **Lamers et al, 2013** [^10.1177/1352458513475832^](https://doi.org/10.1177/1352458513475832) | n=30 (50% female) age: 58.2 (10.9)  Type: PP: n=5, SP: n=25 | **Severity:** EDSS: 7.5 (median), range: 7.0-8.0 **Disease duration:** 21.8 (11) | **healthy** n=30 (33% female) age: 57.9 (10.9) | **Motionlogger** accelerometer 3 axes 2 wearable(s) Position: wrist | **RW: Physical activity, Lab: Physical activity** Association with other measure (s) Group differences MS vs HC (s) |
| **Sandroff et al, 2013** [^10.3109/09638288.2012.707745^](https://doi.org/10.3109/09638288.2012.707745) | n=41 (88% female) age: 47.4 (8.8)  Type: RR: n=37, not reported: n=4 | **Severity:** PDDS: 1.0 (median), range: 0-4 **Disease duration:** 11.0 (7.9) | **healthy** n=41 (88% female) age: 47.2 (9.1) | **ActiGraph 7164** accelerometer 1 axis 1 wearable(s) Position: waist  **ActiGraph GTX3** accelerometer 3 axes 1 wearable(s) Position: waist | **RW: Physical activity, Lab: Physical activity** (other type of result) |
| **Motl et al, 2014** [^10.1159/000356116^](https://doi.org/10.1159/000356116) | n=82 (80% female) age: 49.2 (9.0)  Type: RR: n=67, not reported: n=15 | **Severity:** PDDS: 3.0 [3] **Disease duration:** 11.8 (8.2) | none | **ActiGraph GT3X** accelerometer 3 axes 1 wearable(s) Position: waist | **RW: Physical activity, Lab: Physical activity** Test-retest reliability (s) |
| **Sandroff et al, 2014** [^10.1016/J.MSARD.2013.04.003^](https://doi.org/10.1016/J.MSARD.2013.04.003) | n=212 (80% female) age: 50.0 (10.3)  Type: RR: n=173, PP: n=12, SP: n=22, not reported: n=5 | **Severity:** PASAT: 42.6 (12.2), SDMT: 46.3 (11.6) **Disease duration:** 11.4 (8.8) | none | **ActiGraph GT3X** accelerometer 3 axes 1 wearable(s) Position: waist | **RW: Physical activity** Association with other measure (s) |
| **Spain et al, 2014** [^10.1016/J.GAITPOST.2013.12.010^](https://doi.org/10.1016/J.GAITPOST.2013.12.010) | n=27 (67% female) age: 41 (mean), range: 24-67  Type: RR: n=27 | **Severity:** SR-EDSS: 3.3 (mean), 4.0 (median), range: 0-5.5 **Disease duration:** 10 (mean), 5 (median), range: 0-46 | **healthy** n=18 (78% female) age: 34 (mean), range: 27-60 | **Xsens** accelerometer, gyroscope 3 axes 6 wearable(s) Position: waist | **RW: Physical activity, Lab: Physical activity, Lab: Gait** Group differences MS vs HC (s) Group differences MS vs MS (s) Responsiveness to change (ss) |
| **Kasser et al, 2015** [^10.3109/09638288.2015.1019008^](https://doi.org/10.3109/09638288.2015.1019008) | n=10 (80% female) age: 52 (mean), 95% CI: 45-59  Type: RR: n=7, PP: n=2, SP: n=1 | **Severity:** EDSS: 3.1 (1.73), range: 1-6 | none | **ActiGraph GT1M** accelerometer 2 axes 7 wearable(s) Position: sternum, waist, wrist, ankle, not reported  **APDM Opal IMU** accelerometer, gyroscope, magnetometer 3 axes 6 wearable(s) Position: wrist, ankle, waist, others (chest) | **RW: Physical activity, Lab: Gait, Lab: Balance** Association with MS severity (ss) Association with other measure (ss) Responsiveness to intervention (ss) |
| **Stellmann et al, 2015** [^10.1371/JOURNAL.PONE.0123822^](https://doi.org/10.1371/JOURNAL.PONE.0123822) | n=28 (64% female) age: 45 [38-51], range: 27-68  Type: RR: n=14, PP: n=5, SP: n=8, not reported: n=1 | **Severity:** EDSS: 3.2 [2.5-4.1], range: 1-6.5 **Disease duration:** 9 [4-17], range: 1-24 | none | **Actibelt** accelerometer 3 axes 1 wearable(s) Position: waist | **RW: Physical activity, Lab: Gait** Association with other measure (ss) |
| **Stellmann et al, 2016** [^10.1016/J.JNS.2016.07.051^](https://doi.org/10.1016/J.JNS.2016.07.051) | n=28 (61% female) age: 49 (8.6)  Type: RR: n=3, PP: n=7, SP: n=18 | **Severity:** EDSS: 4.75 [4-6.5] **Disease duration:** 11.0 (8.4) | none | **Actibelt** accelerometer 3 axes 1 wearable(s) Position: waist | **RW: Physical activity, Lab: Physical activity** Responsiveness to intervention (s) |
| **Boukhvalova et al, 2018** [^10.3389/FNEUR.2018.00740^](https://doi.org/10.3389/FNEUR.2018.00740) | n=76 (57% female) age: 56.54 (mean)  Type: RR: n=23, PP: n=35, SP: n=18 | **Severity:** EDSS: 4.91 (mean) **Disease duration:** 16.71 (mean) | **healthy (caregivers)** n=19 (53% female) age: not reported | **Google Pixel XL 2017 (smartphone)** Custom app touchscreen 1 wearable(s) Position: hand | **Lab: Dexterity/Tremor, RW: Dexterity/Tremor** Association with other measure (s) Group differences MS vs HC (s) |
| **Storm et al, 2018** [^10.1371/JOURNAL.PONE.0196463^](https://doi.org/10.1371/JOURNAL.PONE.0196463) | n=14 (50% female) age: 54.8 (11)  Type: not reported: n=14 | **Severity:** EDSS: range 5-6.5 | none | **Mc Roberts PAM Move Monitor** accelerometer 3 axes 1 wearable(s) Position: lower back  **APDM Opal IMU** accelerometer, gyroscope, magnetometer 3 axes 2 wearable(s) Position: ankle | **RW: Physical activity, Lab: Gait** Group differences MS vs MS (ss) |
| **Boukhvalova et al, 2019** [^10.3389/FNEUR.2019.00358^](https://doi.org/10.3389/FNEUR.2019.00358) | n=93 (54% female) age: MS type: RR: 50 (10.2), SP: 60 (8.2), PP: 58.8 (7.8)  Type: RR: n=35, PP: n=38, SP: n=19, not reported: n=1 | **Severity:** EDSS: RR: 3.3 (1.6), range: 1.0-6.5 SP: 6.0 (1.1), range: 3.5-7.5 PP: 5.7 (1.3), range: 2.5-8.0 **Disease duration:** MS type: RR: 12.4 (10.1), SP: 26.8 (11.2), PP: 15.7 (8.3) | **healthy** n=15 (53% female) age: not reported | **Google Pixel XL 2017 (smartphone)** MS Test Suite app accelerometer 3 axes 1 wearable(s) Position: hand | **RW: Dexterity/Tremor, Lab: Dexterity/Tremor** Association with MS severity (ss) Association with other measure (ss) Group differences MS vs HC (s) |
| **Chitnis et al, 2019** [^10.1038/S41746-019-0197-7^](https://doi.org/10.1038/S41746-019-0197-7) | n=25 (92% female) age: 46.5 (7.4)  Type: not reported: n=25 | **Severity:** EDSS: 3.4 (mean), range: 1.0-6.5 **Disease duration:** 16 (5) | none | **Cardiac and Activity Monitor (CAM)** accelerometer, others (ECG, PPG, skin impedance, temperature, light exposure, barometer) 3 axes 3 wearable(s) (real-world), 9 (lab setting) Position: sternum, lower back, waist, wrist, upper leg, ankle | **RW: Gait, Lab: Gait** Association with MS severity (ss) Association with other measure (ss) |
| **Ehling et al, 2019** [^10.1371/JOURNAL.PONE.0220613^](https://doi.org/10.1371/JOURNAL.PONE.0220613) | n=76 age: 47.9 (8.3)  Type: RR: n=46, PP: n=17, SP: n=13 | **Severity:** EDSS: 3.0 [2-5.5] **Disease duration:** 11.9 (8.8) | none | **ActiGraph GT3X** accelerometer 3 axes 1 wearable(s) Position: not reported | **RW: Physical activity, Lab: Physical activity** Responsiveness to change (ss) |
| **Karle et al, 2020** [^10.3390/IJERPH17239044^](https://doi.org/10.3390/IJERPH17239044) | n=20 (75% female) age: 44.2 (12.2)  Type: RR: n=14, PP: n=2, SP: n=4 | **Severity:** EDSS: 3.1 (1.4), range: 1-6 **Disease duration:** 9.1 (7.7) | none | **ActiGraph GT3X** accelerometer 3 axes 1 wearable(s) Position: waist | **RW: Physical activity, Lab: Physical activity** Association with other measure (ns) |
| **Shah et al, 2020** [^10.1186/S12984-020-00781-4^](https://doi.org/10.1186/S12984-020-00781-4) | n=15 age: 49 (10)  Type: RR: n=15 | **Severity:** inclusion criteria: EDSS <6.0 | **healthy** n=16 (female ratio not reported) age: 45 (11) | **APDM Opal IMU** accelerometer, gyroscope, magnetometer 3 axes 3 wearable(s) Position: lower back, foot | **RW: Gait, Lab: Gait** Group differences MS vs HC (ss) Group differences MS vs MS (ss) |
| **Shema-Shiratzky et al, 2020** [^10.1007/S00415-020-09759-7^](https://doi.org/10.1007/S00415-020-09759-7) | n=44 (73% female) age: 49.2 (10.7)  Type: RR: n=44 | **Severity:** EDSS: 3.5 [2.5-5.0] **Disease duration:** 13.3 (9.3) | **healthy** n=60 (51% female) age: 52.1 (7.1) | **APDM Opal IMU** accelerometer, gyroscope, magnetometer 3 axes 1 wearable(s) Position: lower back | **RW: Gait, Lab: Gait** Group differences MS vs HC (ss) |
| **Atrsaei et al, 2021** [^10.1109/JBHI.2021.3076707^](https://doi.org/10.1109/JBHI.2021.3076707) | n=35 (66% female) age: 49.7 (13.0)  Type: not reported: n=35 | **Severity:** EDSS: 4.7 (1.0) | none | **Gait Up Physilog 5 IMU** accelerometer, gyroscope 3 axes 3 wearable(s) Position: waist, foot | **RW: Gait, Lab: Gait** Association with other measure (ns) |
| **Cederberg et al, 2021** [^10.1016/j.sleep.2021.06.005^](https://doi.org/10.1016/j.sleep.2021.06.005) | n=20 (75% female) age: 51.2 (12.3)  Type: RR: n=14 P: n=6 | **Severity:** EDSS: 3.5 (median), IQR : 2.875 **Disease duration:** 14.4 (9.3) | **MS patients** n=20 (75% female) age: 49.8 (11.9) EDSS: 4.0 (median), IQR: 2.5 Disease duration: 13.7 (8.0) | **ActiGraph GT3X+** accelerometer 3 axes 1 wearable(s) Position: foot  **CamNtech Motionwatch 8** accelorometer 3 axes 1 wearable(s) Position: foot | **Lab: Dexterity/Tremor** Association with other measure (ss) Test-retest reliability (ss) Group differences MS vs MS (s) |
| **Gulde et al, 2021** [^10.3390/jcm10102177^](https://doi.org/10.3390/jcm10102177) | n=28 (54% female) age: 46.3 (11.2)  Type: RR: n=19, P: n=9 | **Severity:** EDSS: 3.3 (1.4), range: 1.0-6.5 **Disease duration:** 13.9 (11.7) | none | **Microsoft Lumia 550 (smartphone)** accelerometer, touchscreen 3 axes 1 wearable(s) Position: hand, sternum  **ActiGraph wGT3X-BTz** accelerometer 3 axes 1 wearable(s) Position: wrist | **RW: Physical activity, Lab: Gait** Association with MS severity (ss) Responsiveness to intervention (ss) |
| **Pau et al, 2021** [^10.1097/MD.0000000000024931^](https://doi.org/10.1097/MD.0000000000024931) | n=31 (55% female) age: 52.5 (11.3)  Type: RR: n=31 | **Severity:** EDSS: 3.1 (1.7), range: 1.0-6.0 | none | **ActiGraph GT3X** accelerometer 3 axes 1 wearable(s) Position: wrist  **BTS Bioengineering G-Sensor** accelerometer, gyroscope, magnetometer 3 axes 1 wearable(s) Position: lower back | **RW: Physical activity, Lab: Gait** Association with other measure (ss) |
| **van Oirschot, et al, 2021** [^10.2196/29128^](https://doi.org/10.2196/29128) | n=25 (92% female) age: 40 (8)  Type: RR: n=25 | **Severity:** EDSS: 3.1 (1.4) **Disease duration:** 6 (4.4) | **healthy (matched)** n=21 (81% female) age: 37 (8)  **healthy (normative)** n=58 (50% female) age: 34 (8) | **Bring-your-own-smartphone** MS sherpa app others (GPS) 1 wearable(s) Position: trouser pocket | **Lab: Physical activity, RW: Physical activity** Association with MS severity (ns) Association with other measure (ss) Test-retest reliability (ns) Group differences MS vs HC (s) Subjective participant acceptability (ns) |
| **Scott et al, 2022** [^10.1186/s12984-022-01116-1^](https://doi.org/10.1186/s12984-022-01116-1) | n=20 (45% female) age: 48.7 (9.7)  Type: not reported: n=20 | **Severity:** EDSS: 3.5 (1.7) | **mixed** healthy: n=20 (45% female), age: 71.7 (5.8); PD: n=20 (20% female), age: 69.8 (7.2); Proximal femure fracture: n=19 (58% female), age: 80.0 (8.5); Chronic obstructive pulmonary disease: n=17 (47% female), age: 69.4 (9.1); Congestive heart failure: n=12 (33% female), age: 69.1 (11.7) | **INDIP system with IMUs** accelerometer, gyroscope, others (pressure insoles, infrared time-of-flight distance sensors) 3 axes 7 wearable(s) Position: foot, ankle, lower back | **Lab: Gait, RW: Gait** (other type of result) |
| **Tulipani et al, 2022** [^10.1016/j.gaitpost.2022.02.016^](https://doi.org/10.1016/j.gaitpost.2022.02.016) | n=37 age: 50.6 (12.3)  Type: not reported: n=37 | **Severity:** EDSS: 2.7 (1.3) | **MS patients (non-fallers)** n=16 (female ratio not reported) age: 44.5 (12.8) Severity: EDSS: 2.0 (0.8) Disease duration: not reported | **Biostamp MC10** accelerometer 3 axes 1 wearable(s) Position: right upper leg | **Lab: Balance, RW: Balance** Group differences MS vs MS (ss) |
| **Tulipani et al, 2022** [^10.1109/TNSRE.2022.3169962^](https://doi.org/10.1109/TNSRE.2022.3169962) | n=37 age: 50.6 (12.3)  Type: not reported: n=37 | **Severity:** EDSS: 2.6 (1.4) | **MS patients** non-fallers: n=16, age: 44.7 (12.4); fallers: n=21, age: 55.2 (10.3); no pyramidal impairment: n=23, age: 46.6 (12.4); pyramidal impairment: n=14, age: 57.3 (8.9); no sensory impairment: n=13, age: 45.8 (12.9); sensory impairment: n=24, age: 53.3 (11.3) | **Biostamp MC10** accelerometer 3 axes 2 wearable(s) Position: sternum, upper leg | **Lab: Balance, RW: Balance** Group differences MS vs MS (ss) |
