# Supplementary material for: Wearable Sensor Technologies to Assess Motor Functions in People With Multiple Sclerosis: Systematic Scoping Review and Perspective
Source: J Med Internet Res. 2023 Jul 27;25:e44428. doi: 10.2196/44428 (PMC10415952; doi:10.2196/44428)
Supplement: Multimedia Appendix 12 [file jmir_v25i1e44428_app12.docx]

**Multimedia Appendix 12: Reporting quality assessment of included studies**

1. Does the study have a clearly defined research objective (including an outcome)?
2. Does the study adequately describe the inclusion/exclusion criteria?
3. Does the study report on the population parameters/demographics (at least age, sex)?
4. Does the study report details on assessment of MS (severity [EDSS or PDSS], type)?
5. Does the study provide sufficient details on the wearables used (type, positioning of wearable, context, recording frequency)?
6. Does the study apply proper statistical analysis? Correction for multiple comparisons?
7. Does the study adequately report the strength of the results (e.g., ways of calculating effect sizes, reporting confidence intervals/standard deviation)?
8. Does the study make the data and/or code publicly available?
9. Do the authors report on the limitations of their study?

| **First author and year** | **DOI** | **1. Research objective** | **2. Inclusion / exclusion criteria** | **3. Population parameters** | **4. Details on MS assessments** | **5. Details on wearables** | **6. Appropriate statistical analysis** | **7. Reporting of the strength of the results** | **8. Public data and/or code** | **9. Limitations** |
| --- | --- | --- | --- | --- | --- | --- | --- | --- | --- | --- |
| Ng et al, 1997 | 10.1097/00005768-199704000-00014 | yes | yes | yes | yes | yes | yes | yes | no | yes |
| Motl et al, 2006 | 10.1080/09638280600551476 | yes | no | yes | yes | yes | yes | yes | no | yes |
| Motl et al, 2006 | 10.1002/NUR.20161 | yes | yes | yes | partially | partially | partially | yes | no | yes |
| Motl et al, 2006 | 10.1207/S15324796ABM3202_13 | yes | yes | yes | partially | partially | partially | yes | no | yes |
| Gosney et al, 2007 | 10.1097/01.FCH.0000264411.20766.0C | yes | no | yes | partially | yes | yes | yes | no | no |
| Hale et al, 2007 | 10.1682/JRRD.2005.09.0155 | yes | yes | yes | yes | partially | yes | yes | no | yes |
| Kayes et al, 2007 | 10.1177/0269215507075516 | yes | partially | yes | no | yes | partially | yes | no | yes |
| Kos et al, 2007 | 10.1080/07420520701282364 | yes | yes | yes | partially | yes | partially | yes | no | yes |
| Motl et al, 2007 | 10.1037/0090-5550.52.2.143 | yes | yes | yes | partially | partially | partially | yes | no | yes |
| Motl et al, 2007 | 10.1037/0090-5550.52.4.463 | yes | no | yes | partially | partially | partially | yes | no | yes |
| Motl et al, 2007 | 10.1123/APAQ.24.3.245 | yes | yes | yes | partially | yes | yes | yes | no | yes |
| Hale et al, 2008 | 10.1016/J.APMR.2008.02.027 | yes | yes | yes | no | yes | partially | yes | no | yes |
| Klassen et al, 2007 | 10.1177/0269215507082740 | yes | yes | yes | partially | yes | yes | yes | no | yes |
| Motl et al, 2008 | 10.1007/S12160-008-9049-4 | yes | yes | yes | partially | partially | partially | yes | no | yes |
| Motl et al, 2008 | 10.1016/J.JNS.2007.11.003 | yes | no | yes | yes | partially | partially | yes | no | yes |
| Motl et al, 2008 | 10.1097/NMD.0B013E318177351B | yes | no | yes | yes | partially | partially | yes | no | yes |
| Snook et al, 2008 | 10.1016/J.JPAINSYMMAN.2007.09.007 | yes | partially | yes | yes | yes | partially | yes | no | yes |
| Kayes et al, 2009 | 10.1016/J.APMR.2008.10.012 | yes | yes | yes | partially | yes | partially | yes | no | yes |
| Motl et al, 2009 | 10.1037/A0015770 | yes | yes | yes | partially | yes | partially | yes | no | yes |
| Motl et al, 2009 | 10.1037/A0015985 | yes | yes | yes | yes | partially | yes | yes | no | yes |
| Motl et al, 2009 | 10.1016/J.JPAINSYMMAN.2008.08.004 | yes | yes | yes | partially | partially | yes | yes | no | yes |
| Motl et al, 2009 | 10.1097/MRR.0B013E328325A5ED | yes | yes |  | partially | partially | yes | yes | no | yes |
| Motl et al, 2009 | 10.1080/13548500802241902 | yes | yes | yes | partially | partially | yes | yes | no | yes |
| Motl et al, 2009 | 10.1016/J.JNS.2009.06.015 | yes | partially | no | yes | partially | partially | yes | no | no |
| Motl et al, 2009 | 10.1016/J.APMR.2009.03.020 | yes | yes | yes | yes | yes | partially | yes | no | yes |
| Snook et al, 2009 | 10.1177/0269215508101757 | yes | yes | yes | yes | yes | partially | yes | no | yes |
| Gijbels et al, 2010 | 10.1177/1352458510361357 | yes | yes | yes | yes | yes | yes | yes | no | yes |
| Motl et al, 2010 | 10.1016/J.APMR.2010.08.011 | yes | yes | yes | yes | yes | yes | yes | no | yes |
| Motl et al, 2010 | 10.1097/NMD.0B013E3181D14131 | yes | yes | yes | yes | yes | partially | yes | no | yes |
| Motl et al, 2010 | 10.1016/J.MEDENGPHY.2010.08.015 | yes | yes | yes | yes | yes | yes | yes | no | yes |
| Rietberg et al, 2010 | 10.1016/J.APMR.2010.07.018 | yes | yes | yes | yes | yes | yes | yes | no | yes |
| Sosnoff et al, 2010 | 10.1177/1352458510373111 | yes | yes | yes | yes | yes | partially | yes | no | yes |
| Suh et al, 2010 | 10.1016/J.DHJO.2009.09.002 | yes | no | yes | partially | partially | partially | partially | no | yes |
| Weikert et al, 2010 | 10.1016/J.JNS.2009.12.021 | yes | yes | yes | yes | yes | partially | yes | no | yes |
| Alaqtash et al, 2011 | 10.1016/J.ENGAPPAI.2011.04.010 | yes | no | yes | no | yes | no | yes | no | no |
| Grčić et al, 2011 | 10.12659/MSM.882130 | yes | yes | yes | yes | yes | partially | yes | no | yes |
| Weikert et al, 2011 | 10.7224/1537-2073-13.4.170 | yes | yes | yes | yes | yes | partially | yes | no | yes |
| Motl et al, 2011 | 10.1080/08964289.2011.636769 | yes | yes | yes | yes | yes | partially | yes | no | yes |
| Schlesinger et al, 2011 | 10.1055/S-0031-1271750 | yes | partially | yes | no | yes | partially | yes | no | no |
| Schmidt et al, 2011 | 10.1519/JPT.0B013E31820AA921 | yes | yes | yes | partially | yes | partially | partially | no | yes |
| Coote et al, 2012 | 10.1016/J.APMR.2012.05.010 | yes | yes | yes | yes | partially | partially | yes | no | yes |
| Motl et al, 2012 | 10.1037/A0025965 | yes | yes | yes | partially | partially | partially | yes | no | yes |
| Motl et al, 2012 | 10.1016/J.GAITPOST.2011.09.005 | yes | yes | yes | yes | yes | partially | yes | no | yes |
| Pilutti et al, 2012 | 10.1155/2012/868256 | yes | yes | yes | yes | partially | partially | no | no | yes |
| Ranadive et al, 2012 | 10.1249/MSS.0B013E31822D7997 | yes | yes | yes | yes | partially | partially | yes | no | yes |
| Sandroff et al, 2012 | 10.1111/J.1600-0404.2011.01634.X | yes | yes | yes | yes | partially | yes | yes | no | yes |
| Sandroff et al, 2012 | 10.1682/JRRD.2011.03.0063 | yes | yes | yes | partially | yes | partially | yes | no | yes |
| Sosnoff et al, 2012 | 10.1155/2012/315620 | yes | yes | yes | yes | partially | partially | yes | no | yes |
| Sosnoff et al, 2012 | 10.1682/JRRD.2011.11.0218 | yes | no | yes | yes | partially | partially | yes | no | yes |
| Spain et al, 2012 | 10.1016/J.GAITPOST.2011.11.026 | yes | yes | yes | yes | yes | no | yes | no | no |
| Weikert et al, 2012 | 10.1016/J.MEDENGPHY.2011.09.005 | yes | yes | yes | yes | yes | partially | yes | no | yes |
| Yu et al, 2012 | 10.1088/0967-3334/33/12/2033 | yes | yes | no | no | yes | partially | yes | no | yes |
| Dlugonski et al, 2013 | 10.1016/J.APMR.2012.12.014 | yes | yes | yes | yes | yes | partially | yes | no | yes |
| Grčić et al, 2013 | 10.1007/S13760-013-0187-5 | yes | yes | yes | partially | partially | partially | yes | no | yes |
| Hilfiker et al, 2013 | 10.1186/1756-0500-6-260 | yes | yes | yes | partially | yes | yes | yes | no | yes |
| Huisinga et al, 2013 | 10.1007/S10439-012-0697-Y | yes | yes | yes | partially | yes | partially | yes | no | yes |
| Lamers et al, 2013 | 10.1177/1352458513475832 | yes | yes | yes | yes | partially | partially | yes | no | yes |
| Learmonth et al, 2013 | 10.1177/1352458513483890 | yes | yes | yes | yes | partially | yes | yes | no | yes |
| Learmonth et al, 2013 | 10.1186/1471-2377-13-37 | yes | yes | yes | yes | partially | partially | yes | no | yes |
| Morrison et al, 2013 | 10.1016/J.JNS.2012.10.007 | yes | yes | yes | partially | yes | partially | yes | no | yes |
| Motl et al, 2013 | 10.2522/PTJ.20120479 | yes | yes | yes | yes | partially | partially | yes | no | yes |
| Motl et al, 2013 | 10.1111/ANE.12036 | yes | yes | yes | yes | partially | partially | yes | no | yes |
| Motl et al, 2013 | 10.1371/JOURNAL.PONE.0073247 | yes | yes | yes | yes | partially | yes | yes | no | yes |
| Sandroff et al, 2013 | 10.3109/09638288.2012.707745 | yes | yes | yes | yes | yes | partially | yes | no | yes |
| Sandroff et al, 2013 | 10.1016/J.MHPA.2013.08.001 | partially | yes | yes | yes | yes | partially | yes | no | yes |
| Balantrapu et al, 2014 | 10.1155/2014/649390 | partially | yes | yes | yes | partially | partially | yes | no | yes |
| Carpinella et al, 2014 | 10.1186/1743-0003-11-67 | yes | yes | yes | yes | yes | yes | yes | no | yes |
| Huisinga et al, 2014 | 10.1016/J.APMR.2014.01.004 | yes | yes | yes | yes | partially | no | partially | no | yes |
| Ickmans et al, 2014 | 10.1016/J.CLINEURO.2014.04.021 | yes | yes | yes | partially | partially | partially | yes | no | yes |
| Motl et al, 2014 | 10.1159/000356116 | yes | yes | yes | yes | partially | yes | yes | no | yes |
| Motl et al, 2014 | 10.1080/08964289.2013.821966 | yes | no | yes | yes | partially | partially | yes | no | yes |
| Sandroff et al, 2014 | 10.1016/J.MSARD.2013.04.003 | yes | yes | yes | yes | yes | partially | yes | no | yes |
| Sandroff et al, 2014 | 10.1016/J.MSARD.2013.06.014 | yes | yes | yes | yes | partially | partially | yes | no | yes |
| Sandroff et al, 2014 | 10.1371/JOURNAL.PONE.0093511 | yes | yes | yes | yes | partially | partially | yes | no | yes |
| Sandroff et al, 2014 | 10.1016/J.JNS.2014.02.024 | yes | yes | yes | yes | partially | partially | yes | no | yes |
| Schwartzt et al, 2014 | 10.1016/J.JNS.2014.10.021 | yes | yes | yes | yes | partially | partially | yes | no | no |
| Shammas et al, 2014 | 10.1186/1475-925X-13-10 | yes | yes | yes | yes | partially | partially | yes | no | yes |
| Spain et al, 2014 | 10.1016/J.GAITPOST.2013.12.010 | no | yes | yes | yes | yes | yes | yes | no | yes |
| Suh et al, 2014 | 10.1007/S12529-013-9382-2 | yes | yes | yes | yes | partially | yes | yes | no | yes |
| Ayache et al, 2015 | 10.1016/J.NEUCLI.2015.09.013 | yes | yes | yes | partially | partially | yes | yes | no | yes |
| Ayache et al, 2015 | 10.1016/J.JNS.2015.09.360 | yes | yes | yes | yes | yes | yes | partially | no | yes |
| Blikman et al, 2015 | 10.1016/J.APMR.2014.08.023 | yes | yes | yes | yes | yes | yes | yes | no | yes |
| Bove et al, 2015 | 10.1212/NXI.0000000000000162 | yes | no | yes | yes | partially | partially | yes | no | yes |
| Fjeldstad et al, 2015 | 10.7224/1537-2073.2014-037 | yes | yes | partially | yes | partially | partially | yes | no | yes |
| Carpinella et al, 2015 | 10.1088/1741-2560/12/4/046011 | yes | yes | yes | yes | yes | yes | yes | no | yes |
| Ezeugwu et al, 2015 | 10.1016/J.PMEDR.2015.03.007 | yes | yes | yes | yes | yes | partially | yes | no | yes |
| Hubbard et al, 2015 | 10.1139/APNM-2014-0271 | yes | yes | yes | yes | yes | partially | yes | no | yes |
| Gong et al, 2015 | 10.1109/BSN.2015.7299400 | yes | no | no | no | yes | yes | yes | no | no |
| Gong et al, 2015 | 10.4108/EAI.28-9-2015.2261504 | yes | no | no | no | yes | yes | yes | no | partially |
| Kahraman et al, 2015 | 10.1016/J.CLINEURO.2015.07.018 | yes | yes | yes | yes | partially | yes | yes | no | yes |
| Kasser et al, 2015 | 10.3109/09638288.2015.1019008 | yes | yes | yes | yes | yes | partially | no | no | yes |
| Klaren et al, 2015 | 10.1155/2015/482536 | yes | yes | yes | yes | partially | partially | yes | no | yes |
| Moon et al, 2015 | 10.1155/2015/964790 | yes | yes | yes | yes | yes | yes | yes | no | yes |
| Motl et al, 2015 | 10.7224/1537-2073.2014-016 | yes | yes | yes | yes | partially | yes | yes | no | yes |
| Rice et al, 2015 | 10.1016/J.APMR.2015.06.011 | yes | yes | yes | yes | yes | yes | yes | no | yes |
| Sandroff et al, 2015 | 10.1097/NPT.0000000000000087 | yes | yes | yes | yes | yes | partially | yes | no | yes |
| Sandroff et al, 2015 | 10.1016/J.GAITPOST.2014.10.011 | yes | yes | yes | yes | partially | partially | yes | no | yes |
| Sola-Valls et al, 2015 | 10.1007/S00415-015-7764-X | yes | yes | yes | yes | partially | partially | yes | no | yes |
| Solomon et al, 2015 | 10.1186/S12984-015-0066-9 | yes | yes | yes | partially | yes | yes | yes | no | no |
| Stellmann et al, 2015 | 10.1371/JOURNAL.PONE.0123822 | yes | no | yes | yes | yes | partially | yes | no | yes |
| Balto et al, 2016 | 10.1177/2055217316634754 | partially | yes | yes | yes | partially | partially | yes | no | yes |
| Brodie et al, 2016 | 10.1080/10255842.2016.1140747 | yes | no | yes | partially | partially | yes | yes | no | yes |
| Grover et al, 2016 | 10.1016/J.JPEDS.2016.08.104 | yes | yes | yes | partially | partially | partially | yes | no | yes |
| Gong et al, 2016 | 10.1109/JBHI.2016.2589902 | partially | no | yes | partially | yes | yes | yes | no | yes |
| Kinnett-Hopkins et al, 2016 | 10.1016/J.MSARD.2016.08.010 | yes | yes | yes | yes | partially | yes | yes | no | yes |
| Klaren et al, 2016 | 10.1123/APAQ.2015-0007 | yes | no | yes | yes | yes | yes | yes | no | yes |
| Klaren et al, 2016 | 10.14336/AD.2015.1025 | yes | yes | yes | yes | partially | partially | yes | no | yes |
| Engelhard et al, 2016 | 10.1016/J.GAITPOST.2016.07.184 | yes | partially | yes | yes | yes | yes | yes | no | no |
| Pau et al, 2016 | 10.1016/J.MSARD.2016.10.007 | partially | yes | yes | partially | partially | yes | yes | no | yes |
| Dandu et al, 2016 | 10.1109/BSN.2016.7516271 | yes | no | partially | partially | yes | yes | yes | no | no |
| Stellmann et al, 2016 | 10.1016/J.JNS.2016.07.051 | yes | yes | yes | yes | partially | partially | yes | no | yes |
| Brown et al, 2016 | 10.7224/1537-2073.2015-035 | partially | yes | yes | yes | partially | yes | yes | no | yes |
| Zoerner et al, 2016 | 10.1177/1352458515622695 | yes | yes | yes | yes | yes | yes | yes | no | no |
| Qureshi et al, 2017 | 10.1109/BSN.2017.7936025 | no | no | no | no | no | yes | partially | no | no |
| Aburub et al, 2017 | 10.1016/J.MSARD.2016.12.010 | yes | yes | partially | yes | yes | yes | partially | no | yes |
| Coulter et al, 2017 | 10.1016/J.MEDENGPHY.2017.03.008 | yes | yes | yes | partially | yes | yes | yes | no | yes |
| Craig et al, 2017 | 10.1186/S12984-017-0251-0 | yes | yes | yes | yes | yes | yes | yes | no | yes |
| Craig et al, 2017 | 10.1016/J.CLINBIOMECH.2017.07.011 | yes | yes | partially | yes | yes | partially | partially | no | yes |
| Dalla-Costa et al, 2017 | 10.1016/J.JNS.2017.10.043 | yes | partially | yes | partially | partially | yes | yes | no | yes |
| El-Gohary et al, 2017 | 10.1016/J.APMR.2017.01.030 | yes | yes | yes | yes | yes | yes | yes | no | yes |
| Klaren et al, 2017 | 10.2217/NMT-2016-0036 | yes | yes | yes | yes | partially | partially | partially | no | yes |
| Klaren et al, 2017 | 10.1123/JPAH.2016-0335 | yes | yes | yes | yes | yes | yes | partially | no | yes |
| Krueger et al, 2017 | 10.1186/S12883-016-0783-0 | yes | yes | yes | yes | partially | partially | partially | no | partially |
| Lorefice et al, 2017 | 10.1007/S00415-017-8612-Y | yes | yes | yes | yes | partially | yes | yes | no | no |
| McGinnis et al, 2017 | 10.1371/JOURNAL.PONE.0178366 | yes | yes | yes | partially | yes | yes | yes | no | yes |
| Motl et al, 2017 | 10.1016/J.JNS.2016.11.070 | yes | yes | yes | partially | partially | yes | partially | no | yes |
| Norris et al, 2017 | 10.1016/J.GAITPOST.2017.02.005 | yes | yes | yes | partially | partially | yes | yes | no | yes |
| Pau et al, 2017 | 10.1016/J.GAITPOST.2017.08.023 | yes | yes | yes | partially | yes | partially | yes | no | partially |
| Pau et al, 2017 | 10.1016/J.MSARD.2017.04.002 | yes | yes | yes | yes | no | yes | yes | no | yes |
| Sebastião et al, 2017 | 10.1097/PHM.0000000000000581 | yes | yes | yes | yes | yes | yes | yes | no | yes |
| Sebastião et al, 2017 | 10.3233/NRE-161401 | yes | yes | yes | yes | partially | yes | partially | no | yes |
| Teufl et al, 2017 | 10.1177/0308022617726259 | yes | yes | yes | partially | yes | yes | partially | no | yes |
| Bernhard et al, 2018 | 10.1186/S12883-018-1111-7 | yes | yes | no | no | yes | yes | yes | no | yes |
| Boukhvalova et al, 2018 | 10.3389/FNEUR.2018.00740 | yes | yes | yes | yes | yes | yes | yes | no | no |
| Carpinella et al, 2018 | 10.1109/TNSRE.2018.2881324 | yes | yes | yes | no | yes | yes | yes | no | yes |
| Cederberg, 2018 | 10.1123/JAPA.2016-0358 | yes | yes | yes | yes | partially | yes | yes | no | yes |
| Dasmahapatra et al, 2018 | 10.1159/000488040 | no | partially | yes | yes | yes | yes | yes | no | yes |
| Engelhard et al, 2018 | 10.1016/J.GAITPOST.2017.10.015 | yes | yes | yes | yes | partially | yes | yes | no | yes |
| Fakolade et al, 2018 | 10.3138/PTC.2017-36.EP | yes | yes | yes | no | partially | yes | yes | no | yes |
| Findling et al, 2018 | 10.3389/FNEUR.2018.00686 | yes | yes | yes | yes | partially | yes | yes | no | yes |
| Huisinga et al, 2018 | 10.1016/J.HUMOV.2017.12.009 | yes | yes | partially | yes | yes | yes | yes | no | no |
| Ketelhut et al, 2018 | 10.1080/09638288.2017.1336647 | yes | yes | yes | no | yes | yes | yes | no | yes |
| Motl et al, 2018 | 10.1037/REP0000162 | yes | yes | yes | yes | partially | yes | yes | no | yes |
| Neven et al, 2018 | 10.1177/0361198118772952 | yes | no | yes | no | partially | yes | yes | no | no |
| Pau et al, 2018 | 10.1016/J.MSARD.2017.11.021 | yes | yes | yes | yes | yes | yes | yes | no | yes |
| Psarakis et al, 2018 | 10.1088/1361-6579/AAC0A3 | yes | yes | yes | yes | yes | yes | yes | no | yes |
| Dandu et al, 2018 | 10.1109/JBHI.2017.2773629 | yes | partially | partially | partially | partially | yes | yes | no | yes |
| Sirhan et al, 2018 | 10.1007/S00702-018-1939-4 | yes | yes | yes | yes | partially | yes | yes | no | yes |
| Storm et al, 2018 | 10.1371/JOURNAL.PONE.0196463 | partially | yes | yes | yes | yes | yes | yes | yes | no |
| Sun et al, 2018 | 10.1159/000485958 | yes | yes | yes | yes | yes | yes | yes | no | yes |
| Supratak et al, 2018 | 10.3389/FNEUR.2018.00561 | yes | yes | yes | yes | partially | yes | yes | no | yes |
| Witchel et al, 2018 | 10.3389/FNEUR.2018.00684 | yes | yes | yes | yes | yes | yes | yes | no | yes |
| Anastasi et al, 2019 | 10.1002/PMRJ.12137 | yes | yes | yes | no | yes | yes | yes | no | yes |
| Angelini et al, 2019 | 10.3390/S20010079 | yes | yes | yes | yes | yes | yes | yes | yes | yes |
| Baird et al, 2019 | 10.1016/J.MSARD.2019.07.003 | yes | yes | yes | yes | yes | yes | yes | no | yes |
| Block et al, 2019 | 10.1001/JAMANETWORKOPEN.2019.0570 | yes | yes | yes | yes | no | yes | yes | no | yes |
| Block et al, 2019 | 10.1177/2055217319888660 | yes | yes | yes | yes | yes | yes | yes | no | yes |
| Bollaert et al, 2019 | 10.1519/JPT.0000000000000163 | yes | yes | yes | yes | yes | yes | yes | no | no |
| Boukhvalova et al, 2019 | 10.3389/FNEUR.2019.00358 | yes | yes | yes | yes | partially | yes | yes | partially | no |
| Braakhuis et al, 2019 | 10.1186/S12984-019-0573-1 | yes | yes | yes | yes | yes | yes | yes | no | yes |
| Cederberg et al, 2019 | 10.1016/J.JNS.2019.116531 | yes | yes | yes | yes | yes | yes | yes | no | yes |
| Chitnis et al, 2019 | 10.1038/S41746-019-0197-7 | yes | yes | yes | yes | yes | yes | yes | no | yes |
| Ehling et al, 2019 | 10.1371/JOURNAL.PONE.0220613 | yes | yes | yes | yes | yes | yes | yes | no | yes |
| Flachenecker et al, 2019 | 10.1016/J.MSARD.2019.101903 | yes | yes | yes | yes | yes | yes | partially | no | yes |
| Grinberg et al, 2019 | 10.1016/J.GAITPOST.2019.02.022 | yes | yes | yes | yes | yes | yes | yes | no | yes |
| Kratz et al, 2019 | 10.1093/ABM/KAY018 | yes | yes | yes | yes | partially | yes | yes | no | yes |
| Midaglia et al, 2019 | 10.2196/14863 | yes | yes | yes | yes | no | yes | yes | no | yes |
| Motl et al, 2019 | 10.1016/J.DHJO.2019.05.002 | yes | yes | yes | no | partially | yes | yes | no | yes |
| Motl et al, 2019 | 10.1037/REP0000280 | yes | yes | yes | no | no | yes | yes | no | yes |
| Bollaert et al, 2019 | 10.7224/1537-2073.2018-001 | yes | yes | yes | yes | partially | yes | yes | no | yes |
| Rooney et al, 2019 | 10.1080/09638288.2019.1634768 | yes | yes | yes | yes | yes | yes | yes | no | yes |
| Sasaki et al, 2019 | 10.1080/02640414.2018.1554614 | yes | yes | partially | no | partially | yes | yes | no | yes |
| Shema-Shiratzky et al, 2019 | 10.1007/S00415-019-09500-Z | yes | yes | yes | yes | yes | yes | yes | no | yes |
| Silveira et al, 2019 | 10.1016/J.CONCTC.2019.100366 | yes | yes | yes | yes | partially | yes | yes | no | yes |
| Ader et al, 2020 | 10.3390/BIOS10090128 | yes | yes | yes | yes | yes | yes | yes | no | yes |
| Akhbardeh et al, 2020 | 10.1002/ACN3.50988 | yes | yes | yes | partially | partially | partially | yes | no | yes |
| Angelini et al, 2020 | 10.1007/S00415-020-09928-8 | yes | partially | yes | yes | yes | yes | yes | no | yes |
| Bourke et al, 2020 | 10.3390/S20205906 | yes | yes | yes | yes | yes | partially | yes | no | yes |
| Brull et al, 2020 | 10.3390/S20154329 | yes | partially | no | partially | yes | yes | yes | no | yes |
| Cofré et al, 2020 | 10.1016/J.GAITPOST.2020.02.006 | yes | yes | yes | yes | yes | yes | yes | no | yes |
| Cohen et al, 2020 | 10.1007/S00415-020-10276-W | yes | partially | yes | yes | yes | partially | partially | no | yes |
| Craig et al, 2020 | 10.1016/J.CLINBIOMECH.2020.105100 | yes | yes | yes | partially | yes | yes | partially | no | yes |
| Creagh et al, 2020 | 10.1088/1361-6579/AB8771 | yes | yes | yes | yes | partially | yes | yes | no | yes |
| Hibner et al, 2020 | 10.1016/J.MSARD.2020.101941 | yes | yes | yes | yes | partially | yes | partially | no | yes |
| Huang et al, 2020 | 10.3390/S20216160 | yes | yes | yes | yes | yes | yes | partially | no | yes |
| Ibrahim et al, 2020 | 10.1186/S12984-020-00798-9 | yes | yes | yes | yes | yes | yes | partially | no | yes |
| Daunoraviciene et al, 2020 | 10.1109/MSM49833.2020.9201642 | yes | yes | yes | partially | yes | partially | yes | no | yes |
| Daunoraviciene et al, 2020 | 10.3233/THC-208003 | yes | yes | yes | partially | yes | yes | partially | no | no |
| Mate et al, 2020 | 10.7224/1537-2073.2019-047 | yes | yes | yes | partially | yes | yes | partially | no | yes |
| Karle et al, 2020 | 10.3390/IJERPH17239044 | yes | yes | yes | yes | yes | yes | partially | no | yes |
| Lam et al, 2020 | 10.1177/1352458520968797 | yes | yes | yes | yes | yes | yes | yes | no | yes |
| Maillart et al, 2020 | 10.1111/ENE.14091 | yes | yes | yes | yes | partially | yes | partially | no | yes |
| Meyer et al, 2020 | 10.1109/JBHI.2020.3025049 | yes | yes | yes | partially | yes | partially | partially | no | partially |
| Mosquera-lopez et al, 2020 | 10.1109/JBHI.2020.3041035 | yes | yes | yes | yes | yes | yes | partially | partially | partially |
| Naess-Schmidt et al, 2020 | 10.1080/2331205X.2020.1713280 | partially | yes | yes | no | yes | partially | partially | no | yes |
| Nasseri et al, 2020 | 10.7717/PEERJ.9303 | yes | yes | yes | yes | no | yes | partially | no | yes |
| Neal et al, 2020 | 10.1177/1545968320916159 | yes | yes | yes | yes | yes | partially | partially | no | yes |
| Pau et al, 2020 | 10.3390/IJERPH17238848 | yes | yes | yes | yes | yes | yes | partially | no | yes |
| Pilloni et al, 2020 | 10.1002/ACN3.51224 | yes | yes | yes | yes | yes | yes | yes | no | yes |
| Pratap et al, 2020 | 10.2196/22108 | partially | yes | yes | yes | partially | partially | yes | yes | yes |
| Sandroff et al, 2020 | 10.1017/S1355617720000284 | yes | yes | yes | yes | yes | yes | partially | no | yes |
| Sato et al, 2020 | 10.1016/J.MSARD.2020.102031 | yes | yes | yes | yes | yes | yes | yes | no | yes |
| Schwab et al, 2020 | 10.1109/JBHI.2020.3021143 | yes | partially | partially | no | no | partially | partially | yes | yes |
| Shah et al, 2020 | 10.1186/S12984-020-00781-4 | yes | yes | yes | partially | yes | yes | partially | no | yes |
| Shamlmoni et al, 2020 | 10.1007/S00702-020-02190-2 | yes | yes | yes | yes | partially | yes | yes | no | yes |
| Shema-Shiratzky et al, 2020 | 10.1007/S00415-020-09759-7 | yes | yes | yes | yes | no | yes | yes | no | yes |
| Stuart et al, 2020 | 10.1177/2055217320975185 | yes | yes | yes | yes | yes | yes | partially | no | yes |
| Tulipani et al, 2020 | 10.1016/J.GAITPOST.2020.06.014 | yes | yes | partially | partially | yes | partially | partially | yes | partially |
| Twose et al, 2020 | 10.1063/5.0022031 | yes | yes | no | no | yes | yes | yes | no | yes |
| Shah et al, 2020 | 10.1007/S00415-020-09696-5 | yes | yes | yes | partially | yes | yes | partially | no | yes |
| Vienne-Jumeau et al, 2020 | 10.3389/FNEUR.2020.00261 | yes | yes | yes | yes | yes | yes | yes | no | yes |
| Zhai et al, 2020 | 10.3389/FNEUR.2020.00688 | yes | yes | yes | yes | yes | yes | partially | no | yes |
| Abbadessa et al, 2021 | 10.3390/JCM10061160 | yes | yes | yes | yes | partially | yes | partially | no | yes |
| Abonie et al, 2021 | 10.3390/IJERPH18010017 | yes | yes | yes | yes | yes | partially | yes | no | yes |
| Abonie et al, 2021 | 10.1177/02692155211024135 | yes | yes | yes | yes | yes | partially | partially | no | yes |
| Adam et al, 2021 | 10.23919/AE51540.2021.9542904 | yes | partially | yes | no | yes | partially | yes | no | no |
| Allum et al, 2021 | 10.1016/j.jns.2021.117432 | yes | yes | yes | yes | no | yes | yes | no | yes |
| Anens et al, 2021 | 10.1080/09593985.2021.1996498 | yes | yes | yes | yes | yes | yes | partially | no | yes |
| Angelini et al, 2021 | 10.1109/TBME.2021.3061998 | yes | partially | yes | yes | yes | yes | yes | no | yes |
| Atrsaei et al, 2021 | 10.1109/JBHI.2021.3076707 | yes | yes | yes | yes | yes | yes | yes | no | yes |
| Barrios et al, 2021 | 10.1145/3478098 | yes | partially | yes | yes | partially | yes | yes | no | yes |
| Cederberg et al, 2021 | 10.1016/j.dhjo.2021.101133 | yes | yes | yes | yes | yes | yes | partially | no | yes |
| Cederberg et al, 2021 | 10.1016/j.sleep.2021.06.005 | yes | yes | yes | yes | yes | yes | yes | no | yes |
| Cheng et al, 2021 | 10.1016/J.GAITPOST.2020.11.025 | yes | yes | yes | yes | yes | yes | yes | no | yes |
| Creagh et al, 2021 | 10.1109/JBHI.2020.2998187 | yes | partially | yes | partially | yes | yes | yes | yes | yes |
| Creagh et al, 2021 | 10.1038/s41598-021-92776-x | yes | partially | yes | yes | yes | yes | yes | yes | yes |
| Delahaye et al, 2021 | 10.3390/s21093189 | yes | yes | yes | yes | yes | yes | yes | yes | yes |
| Di Giovanni et al, 2021 | 10.1016/j.msard.2021.103036 | yes | yes | yes | yes | yes | partially | partially | no | partially |
| Eldemir et al, 2021 | 10.1080/21641846.2021.1923995 | yes | yes | yes | yes | yes | yes | yes | no | yes |
| Gulde et al, 2021 | 10.3390/jcm10102177 | yes | yes | yes | yes | yes | yes | yes | no | yes |
| Gulde et al, 2021 | 10.1155/2021/5589562 | yes | yes | yes | yes | yes | yes | yes | no | yes |
| Guo et al, 2021 | 10.1145/3494970 | yes | yes | yes | partially | yes | yes | partially | no | yes |
| Hildebrand et al, 2021 | 10.1016/j.msard.2021.103270 | yes | yes | yes | yes | yes | yes | partially | no | yes |
| Hsieh et al, 2021 | 10.1016/J.GAITPOST.2020.11.011 | yes | yes | yes | yes | yes | partially | yes | no | yes |
| Hsieh et al, 2021 | 10.2196/25604 | yes | yes | yes | yes | partially | yes | yes | no | yes |
| Jeng et al, 2021 | 10.1080/09638288.2019.1614683 | yes | yes | yes | yes | yes | partially | yes | no | yes |
| Khalil et al, 2021 | 10.3233/NRE-210188 | yes | yes | yes | yes | yes | yes | yes | no | yes |
| Krysko et al, 2021 | 10.1002/ACN3.51187 | yes | yes | yes | yes | partially | partially | yes | no | yes |
| Pau et al, 2021 | 10.1097/MD.0000000000024931 | yes | yes | yes | yes | yes | partially | partially | no | yes |
| Motl et al, 2021 | 10.1177/20552173211057514 | yes | yes | yes | yes | yes | yes | partially | no | yes |
| Muller et al, 2021 | 10.1186/s12883-021-02361-y | yes | yes | yes | yes | yes | partially | partially | no | yes |
| Nagasubramony et al, 2021 | 10.1145/3452853.3452862 | yes | partially | no | no | yes | partially | yes | no | no |
| Negaresh et al, 2021 | 10.1111/jon.12869 | yes | yes | yes | yes | yes | yes | yes | no | yes |
| Pau et al, 2021 | 10.1016/j.msard.2021.103081 | yes | yes | yes | yes | yes | yes | partially | no | yes |
| Prochazka et al, 2021 | 10.1109/TNSRE.2021.3051093 | yes | partially | partially | no | partially | yes | yes | no | no |
| Sagawa et al, 2021 | 10.3390/s21113617 | yes | yes | yes | yes | yes | partially | partially | no | yes |
| Shah et al, 2021 | 10.1016/J.GAITPOST.2020.11.024 | yes | yes | partially | partially | yes | partially | partially | no | yes |
| Silveira et al, 2021 | 10.1016/j.dhjo.2021.101163 | yes | yes | yes | yes | yes | yes | yes | no | yes |
| Silveira et al, 2021 | 10.1016/j.dhjo.2020.100966 | yes | yes | yes | yes | no | yes | yes | no | yes |
| Stephens et al, 2021 | 10.1177/1352458520974360 | yes | yes | yes | partially | partially | yes | yes | no | yes |
| Swanson et al, 2021 | 10.1016/j.msard.2021.102924 | yes | partially | yes | yes | yes | yes | yes | no | yes |
| Tanoh et al, 2021 | 10.1016/j.msard.2021.103164 | yes | partially | yes | yes | partially | yes | partially | no | partially |
| Teufl et al, 2021 | 10.1177/2055668320966955 | yes | yes | yes | partially | yes | yes | partially | no | yes |
| Trentzsch et al, 2021 | 10.3390/brainsci11111507 | yes | yes | yes | yes | yes | yes | yes | no | yes |
| van et al, 2021 | 10.3390/sym13091560 | yes | no | partially | partially | partially | yes | yes | partially | yes |
| van Oirschot, et al, 2021 | 10.2196/29128 | yes | yes | yes | yes | yes | yes | yes | no | yes |
| Weed et al, 2021 | 10.3390/s21175806 | yes | yes | yes | yes | yes | yes | yes | no | yes |
| Woelfle et al, 2021 | 10.2196/30394 | yes | yes | yes | no | partially | yes | yes | yes | yes |
| Afzal et al, 2022 | 10.1109/TBME.2022.3166705 | yes | yes | yes | yes | partially | yes | partially | no | yes |
| Alexander et al, 2022 | 10.1177/13524585221124043 | yes | yes | yes | yes | partially | yes | yes | no | yes |
| Arpan et al, 2022 | 10.3390/s22165940 | yes | yes | yes | yes | yes | yes | yes | no | yes |
| Berg-Hansen et al, 2022 | 10.1007/s00415-022-10998-z | yes | yes | yes | yes | yes | yes | yes | no | yes |
| Block et al, 2022 | 10.1007/s00415-021-10743-y | yes | yes | yes | yes | yes | yes | yes | no | yes |
| Block et al, 2022 | 10.3389/fneur.2022.860008 | yes | yes | yes | yes | yes | yes | yes | no | yes |
| Bois et al, 2022 | 10.1371/journal.pone.0268475 | yes | partially | yes | yes | yes | partially | partially | no | yes |
| Brenton et al, 2022 | 10.1212/WNL.0000000000201098 | yes | yes | yes | yes | no | yes | yes | no | yes |
| Carpinella et al, 2022 | 10.3390/s22239558 | yes | yes | yes | yes | yes | yes | yes | no | yes |
| Carpinella et al, 2022 | 10.3389/fneur.2021.821640 | yes | yes | yes | yes | yes | yes | yes | no | yes |
| Cederberg et al, 2022 | 10.1016/j.apmr.2021.12.022 | yes | yes | yes | yes | yes | yes | yes | no | yes |
| Chikersal et al, 2022 | 10.2196/38495 | yes | yes | yes | yes | partially | yes | yes | no | yes |
| Creagh et al, 2022 | 10.1109/OJEMB.2022.3221306 | yes | partially | yes | yes | partially | yes | partially | no | yes |
| Drouin et al, 2022 | 10.1002/sim.9625 | yes | no | no | partially | yes | yes | yes | no | yes |
| Frechette et al, 2022 | 10.2196/32453 | yes | yes | partially | no | partially | partially | no | no | yes |
| Ganzetti et al, 2022 | 10.1007/s00415-022-11494-0 | yes | yes | yes | yes | yes | yes | yes | no | yes |
| Gervasoni et al, 2022 | 10.1016/j.msard.2022.103941 | yes | yes | yes | yes | yes | partially | partially | no | yes |
| Gervasoni et al, 2022 | 10.3389/fimmu.2022.842269 | yes | yes | yes | yes | yes | yes | yes | yes | yes |
| Graves et al, 2022 | 10.1002/acn3.51705 | yes | yes | yes | yes | yes | yes | yes | no | yes |
| Hossen et al, 2022 | 10.1016/j.ebiom.2022.104152 | yes | partially | yes | no | partially | yes | partially | no | yes |
| Huang et al, 2022 | 10.3390/brainsci12020258 | yes | yes | yes | yes | yes | yes | partially | no | yes |
| Huynh et al, 2022 | 10.1016/j.dhjo.2022.101314 | yes | yes | yes | yes | yes | yes | partially | no | yes |
| Hvid et al, 2022 | 10.1007/s00415-022-11134-7 | yes | yes | yes | yes | yes | yes | yes | no | yes |
| Ibrahim et al, 2022 | 10.1016/j.msard.2022.103519 | yes | yes | yes | yes | yes | yes | partially | no | yes |
| Jeng et al, 2022 | 10.1016/j.msard.2021.103312 | yes | yes | yes | yes | yes | partially | partially | no | yes |
| Jeng et al, 2022 | 10.3390/ijerph191912466 | yes | yes | yes | yes | yes | yes | partially | no | yes |
| Jones et al, 2022 | 10.1016/j.msard.2022.103889 | yes | yes | yes | yes | yes | yes | partially | no | yes |
| Keller et al, 2022 | 10.1177/20556683211067362 | yes | yes | yes | yes | yes | yes | yes | no | yes |
| Kim et al, 2022 | 10.1177/15459683221131787 | yes | partially | yes | yes | yes | yes | partially | no | yes |
| Kinnett-Hopkins et al, 2022 | 10.1016/j.dhjo.2022.101344 | yes | yes | yes | yes | yes | yes | partially | no | yes |
| Lam et al, 2022 | 10.2196/37614 | yes | yes | yes | yes | yes | yes | yes | no | yes |
| Lam et al, 2022 | 10.1007/978-981-19-8234-7_20 | yes | yes | no | no | partially | yes | partially | partially | yes |
| Lam et al, 2022 | 10.1111/ene.15162 | yes | yes | yes | yes | yes | yes | yes | no | yes |
| Leblanc et al, 2022 | 10.23736/S0393-3660.20.04442-3 | yes | yes | yes | partially | yes | yes | partially | no | yes |
| Marotta et al, 2022 | 10.3390/jcm11123505 | partially | yes | yes | yes | yes | partially | partially | no | yes |
| Meyer et al, 2022 | 10.3390/s22186982 | yes | yes | yes | partially | yes | yes | partially | no | yes |
| Montalban et al, 2022 | 10.1177/13524585211028561 | yes | yes | yes | yes | yes | yes | partially | no | yes |
| Motl et al, 2022 | 10.1016/j.msard.2022.103833 | yes | no | yes | yes | yes | yes | yes | no | yes |
| Salomon et al, 2022 | 10.1016/j.msard.2022.104108 | yes | yes | yes | yes | yes | yes | partially | no | yes |
| Sandroff et al, 2022 | 10.1177/13524585211048397 | yes | yes | yes | yes | yes | yes | partially | no | yes |
| Sato et al, 2022 | 10.1016/j.clinbiomech.2022.105818 | yes | yes | yes | yes | yes | yes | yes | no | yes |
| Scott et al, 2022 | 10.1186/s12984-022-01116-1 | yes | yes | yes | yes | partially | yes | yes | no | yes |
| Shah et al, 2022 | 10.3390/s22031077 | yes | partially | partially | no | yes | yes | yes | no | yes |
| Stephens et al, 2022 | 10.1016/j.msard.2021.103467 | partially | yes | yes | yes | partially | yes | yes | no | yes |
| Sun et al, 2022 | 10.1016/j.cmpb.2022.107204 | yes | partially | yes | yes | partially | yes | partially | no | yes |
| Tonning et al, 2022 | 10.3389/fspor.2022.1006422 | yes | yes | yes | no | yes | yes | yes | no | yes |
| Tulipani et al, 2022 | 10.1016/j.gaitpost.2022.02.016 | yes | yes | partially | yes | yes | yes | yes | yes | yes |
| Tulipani et al, 2022 | 10.1109/TNSRE.2022.3169962 | yes | yes | partially | yes | yes | yes | partially | no | yes |
| Warmerdam et al, 2022 | 10.3390/data7100136 | no | partially | yes | no | yes | no | no | partially | no |
| Woelfle et al, 2022 | 10.1007/s00415-022-11306-5 | yes | yes | yes | yes | partially | yes | yes | no | yes |
